# Glucose deprivation triggers DCAF1-mediated inactivation of Rheb-mTORC1 and promotes cancer cell survival

**DOI:** 10.1038/s41419-024-06808-1

**Published:** 2024-06-11

**Authors:** Miaomiao Li, Wenjing Huang, Yuan Zhang, Yue Du, Shan Zhao, Longhao Wang, Yaxin Sun, Beibei Sha, Jie Yan, Yangcheng Ma, Jinlu Tang, Jianxiang Shi, Pei Li, Lijun Jia, Tao Hu, Ping Chen

**Affiliations:** 1 Academy of Medical Sciences, School of Basic Medical Sciences, Zhengzhou University, Zhengzhou, 450001 China; 2 The First Affiliated Hospital of Zhengzhou University, Zhengzhou University, Zhengzhou, 450052 China; 3 Sanquan College of Xinxiang Medical University, Xinxiang, 453003 China; 4 The Second Affiliated Hospital of Zhengzhou University, Zhengzhou University, Zhengzhou, 450014 China; 5 Precision Medicine Center, Henan Institute of Medical and Pharmaceutical Sciences & BGI College, Zhengzhou University, Zhengzhou, 450052 China; 6 Cancer Institute, Longhua Hospital, Shanghai University of Traditional Chinese Medicine, Shanghai, 200032 China

**Keywords:** Cancer metabolism, Growth factor signalling

## Abstract

Low glucose is a common microenvironment for rapidly growing solid tumors, which has developed multiple approaches to survive under glucose deprivation. However, the specific regulatory mechanism remains largely elusive. In this study, we demonstrate that glucose deprivation, while not amino acid or serum starvation, transactivates the expression of DCAF1. This enhances the K48-linked polyubiquitination and proteasome-dependent degradation of Rheb, inhibits mTORC1 activity, induces autophagy, and facilitates cancer cell survival under glucose deprivation conditions. This study identified DCAF1 as a new cellular glucose sensor and uncovered new insights into mechanism of DCAF1-mediated inactivation of Rheb-mTORC1 pathway for promoting cancer cell survival in response to glucose deprivation.

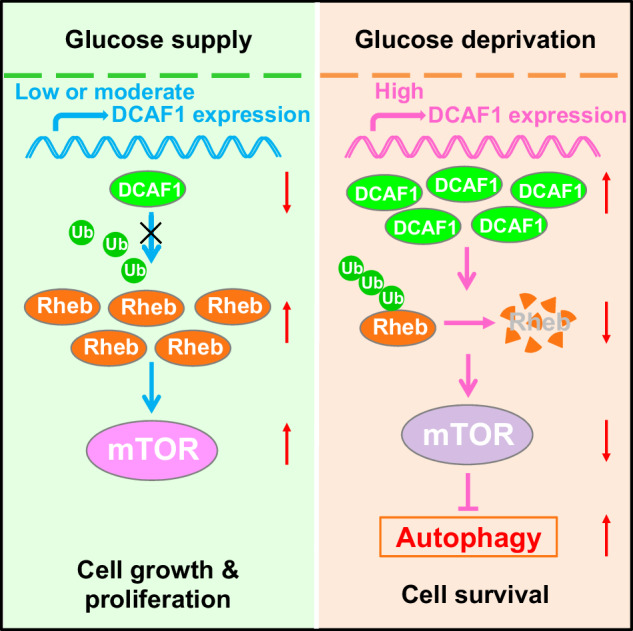

## Introduction

Glucose deficiency is a distinguishing feature of solid tumors, which have developed various mechanisms to survive under low glucose conditions [1]. The mammalian/mechanistic target of rapamycin (mTOR) kinase, a highly conserved serine/threonine protein kinase, serves as a key hub in these mechanisms. mTOR acts as the catalytic subunit of mTORC1 and mTORC2 complexes [2, 3], with mTORC1 mainly modulated by hypoxia, growth factors, nutrients, and energy, et al., and playing an important role in cell proliferation, autophagy, and cell survival [2–5]. Under normal glucose conditions, mTORC1 is activated and promotes cell growth and proliferation. However, under low glucose conditions, mTORC1 activity is suppressed and autophagy is induced to promote cell survival [4, 6–8]. Although mTORC1 signaling is highly sensitive to environmental glucose levels, the glucose sensor for mTORC1 regulation remains largely elusive. Exploring how glucose regulates mTORC1 activity will broaden our insight into the biological function of mTORC1 and enhance our understanding of the survival mechanism of cancer cells characterized by restricted glucose availability.

Accumulating evidence reveals that Ras homologue enriched in brain (Rheb) is an important mTORC1 activator induced by nearly all signals [2, 3], promoting cell proliferation/growth by positively regulating mTORC1 activity [5]. Overexpression of Rheb can restore mTORC1 activity in vitro and in vivo, which is observed when mTORC1 activity is inhibited by amino-acid withdrawal [9] or glucose deprivation [10]. The well-established Rheb regulator is the TSC protein complex, comprising TSC1, TSC2, and TBC1D7 [2, 10–12]. Several upstream kinases, such as Akt, MK2, AMP-activated protein kinase (AMPK), ERK, p90 ribosomal S6 kinase, and GSK-3 β, control Rheb activity through direct modulation of TSC1 and TSC2 [2, 13]. TSC deficiency renders cells more sensitive to glucose deprivation due to uncontrolled activation of Rheb-mTORC1 signaling [2]. However, in TSC2 knockout mouse embryonic fibroblasts, glucose deprivation still inhibits the activity of mTORC1 [14], suggesting that other regulatory mechanisms for Rheb-mTORC1 exist. For example, in response to a decrease in glucose levels, GAPDH interacts with Rheb and prevents it from binding to and activating mTORC1 in TSC or AMPK-deficient cells [5, 14]. Zheng et al. also reported that Rheb could be directly phosphorylated by PRAK, and Rheb-mediated mTORC1 activation was inhibited independent of AMPK-TSC2 and AMPK-Raptor under energy depletion [15]. Recently, several groups reported that Rheb could be directly regulated by ubiquitination and that this manipulation of Rheb affected mTORC1 activity [16–18]. Amino acids were found to enhance the polyubiquitination of Rheb, promoting amino-acid-induced mTORC1 activation by inhibiting lysosomal ATXN3 deubiquitinase activity [16]. Lysosome-anchored E3 ligase RNF152 and deubiquitinase USP4 were shown to regulate growth factor-induced mTORC1 activation by catalyzing the mono-ubiquitination of Rheb [17]. Additionally, Rheb could be degraded by the E3 ubiquitin ligase Siah1, inhibiting the mTOR pathway in response to nitric oxide [18]. These results highlight the distinct regulation modes of Rheb under different types of stress. However, the fate of Rheb and the underlying mechanisms during glucose deprivation remain unclear.

DDB1-CUL4-associated factor 1 (DCAF1), also known as VprBP (Viral protein R binding protein), is a major receptor for the DDB1-CUL4 E3 ubiquitin ligase (CRL4), and HECT type EDD/UBR5 E3 ligase [19, 20]. Previous reports have revealed that DCAF1 is involved in regulating the cell cycle, cell growth, cell division, and cell proliferation by promoting the ubiquitination and degradation of its physiological substrates [20–22]. Initially, DCAF1 was reported to participate in HIV-1 Vpr-induced cell cycle arrest [23, 24]. Subsequently, DCAF1 was found to play a critical role in DNA replication and embryonic development. Silencing DCAF1 resulted in defective progression through S phase, inhibited cell proliferation, triggered apoptosis, and led to early embryonic lethality [25]. Additionally, several reports have revealed that DCAF1 is involved in the development of tumors and other diseases [22, 26–30]. Most recently, DCAF1 was further developed as an attractive target for the development of targeted protein degraders [31–33]. Despite the identification of several DCAF1 substrates, its essential function in cell proliferation or survival during nutrient or energy stress remains unclear. Recently, it was discovered that DCAF1 served as a new-type ubiquitin E3 ligase for nuclear factor erythroid 2-related factor 2 (NRF2) and promoted NRF2 ubiquitination and degradation to regulate NRF2 signaling [34, 35]. Previous reports have also revealed that NRF2 is an important upstream regulator of mTOR [36, 37], suggesting a potential connection between DCAF1 and mTORC1.

Our study demonstrates that glucose deprivation, rather than amino acid or serum withdrawal, induces degradation of Rheb. DCAF1 mediates this process by interacting with Rheb and catalyzing its polyubiquitination and degradation. In response to glucose deprivation, DCAF1 is transactivated and initiates autophagy to promote cancer cell survival by inhibiting Rheb-mTORC1 axis. These findings enhance our understanding of the biological function of the Rheb-mTORC1 pathway under conditions of glucose deprivation, and reveal a novel role for DCAF1 as a glucose sensor that promotes cancer cell survival in low glucose environments.

## Results

### Glucose deprivation induces the ubiquitination and proteasome-dependent degradation of Rheb

**Fig. 1:**
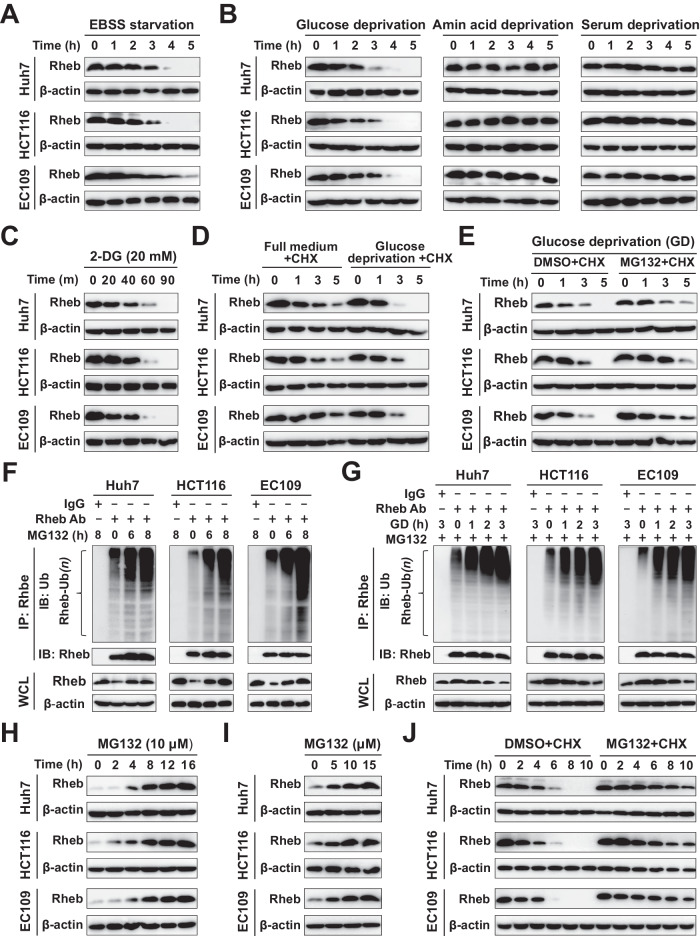
Glucose deprivation induces ubiquitin-mediated degradation of Rheb. **A**–**C** Time-dependent downregulation of Rheb protein level in response to glucose deprivation, but not to amino acids or serum deprivation. Huh7, HCT116, and EC109 cells were grown in EBSS (**A**), glucose-free, amino acids-free, serum-free medium (**B**) or treated with 2-DG (20 mM) (**C**) for the indicated time and subjected to immunoblotting (IB) analysis. **D** Decreased stability of Rheb during glucose deprivation. Cells were grown in full medium (DMEM high-glucose medium) and glucose-free medium containing 100 µg/ml CHX, and the protein level of Rheb was analyzed at the indicated time points. **E** Slowed degradation of Rheb during glucose deprivation by proteasome inhibitor MG132. Cells were grown in glucose-free medium containing CHX (100 µg/ml) + DMSO (0.1%) or 10 µM MG132, and protein levels of Rheb were analyzed at the indicated time points for IB analysis. **F** Increased levels of ubiquitinated Rheb upon MG132 treatment. Cells were treated with 10 µM MG132, and immunoprecipitation (IP) was performed using IgG or Rheb antibody (Ab). The polyubiquitination of Rheb (Rheb-Ub) was detected with Ub Ab. **G** Promotion of polyubiquitination of Rheb under glucose deprivation. Cells were cultured with or without glucose-free medium with 10 µM MG132, and IP was performed using IgG or Rheb Ab. **H**–**J** Enhanced protein stability of Rheb by MG132. Cells were treated with 10 µM MG132 and collected at the indicated time (H) or treated with MG132 at different concentrations (**I**) for IB analysis. **J** Cells were treated with 100 µg/ml CHX + DMSO or 10 µM MG132 and harvested at the indicated time for IB analysis. WCL, whole cell lysate. All data were representative of at least three independent experiments (*n* = 3).

To investigate the effect of nutrient deficiency on Rheb, we assessed the change in Rheb protein levels under nutrient-free medium. We cultured Huh7, HCT116, and EC109 cells in full medium (DMEM high-glucose medium) or nutrient-free medium by culturing cells in Earle’s balanced salt solution (EBSS). The protein levels of Rheb were significantly reduced in a time-dependent manner in response to nutrition deprivation after cells were cultured in EBSS (Fig. 1A), while there was little change when cells were cultured in full medium (Supplementary Fig. 1A). We exposed cells to several stimuli, including glucose, amino acid, and serum withdrawal, to determine the exact upstream nutrient signal that regulate Rheb protein levels. Our results showed that only glucose deprivation effectively reduced Rheb protein levels, while neither amino acid nor serum deprivation had such an effect during the same time period (Fig. 1B). We confirmed these findings with 2-Deoxy glucose (2-DG) treatment, a glycolysis inhibitor, which is often used to mimic the situation of glucose starvation [8] (Fig. 1C). These data suggest a specific regulation of Rheb by glucose deprivation.

Next, to investigate the molecular mechanism underlying the effect of glucose deprivation on Rheb, we examined the half-life of Rheb protein after glucose deprivation, using cycloheximide (CHX) chase assays. Results showed that glucose deprivation decreased the Rheb protein stability, as reflected by a shortened half-life of Rheb (Fig. 1D and Supplementary Fig. 1D). Additionally, MG132, a proteasome inhibitor, enhanced the protein stability of Rheb during glucose deprivation (Fig. 1E and Supplementary Fig. 1E). At the same time, we found that the mRNA level of Rheb exhibited little change after cells were cultured in EBSS or glucose-free medium, or when treated with 2-DG (Supplementary Fig. 1B), implying that Rheb undergone proteasome-mediated degradation in response to glucose deprivation. Moreover, MG132 treatment caused substantially elevated levels of polyubiquitinated Rheb (Rheb-Ub) in a time-dependent manner (Fig. 1F), which was enhanced by glucose deprivation (Fig. 1G). Furthermore, we observed that MG132, which blocked the catalytic activity of the proteasome, significantly increased the protein abundance of Rheb in a time- and dose-dependent manner (Fig. 1H, I) and enhanced the Rheb protein stability (Fig. 1J and Supplementary Fig. 1F). Conversely, bafilomycin A1 (BafA1), a classical lysosomal inhibitor, had no effect on the protein abundance of Rheb, although the protein abundance of p62 and LC3 were significantly accumulated under the same condition (Supplementary Fig. 1C). Altogether, these findings indicated that glucose deprivation specifically downregulated Rheb *via* polyubiquitination and proteasome-dependent degradation, which was observed in all the tested cell lines, implying that this mechanism may be a universal phenomenon.

### DCAF1 interacts with Rheb and regulates its ubiquitination

**Fig. 2:**
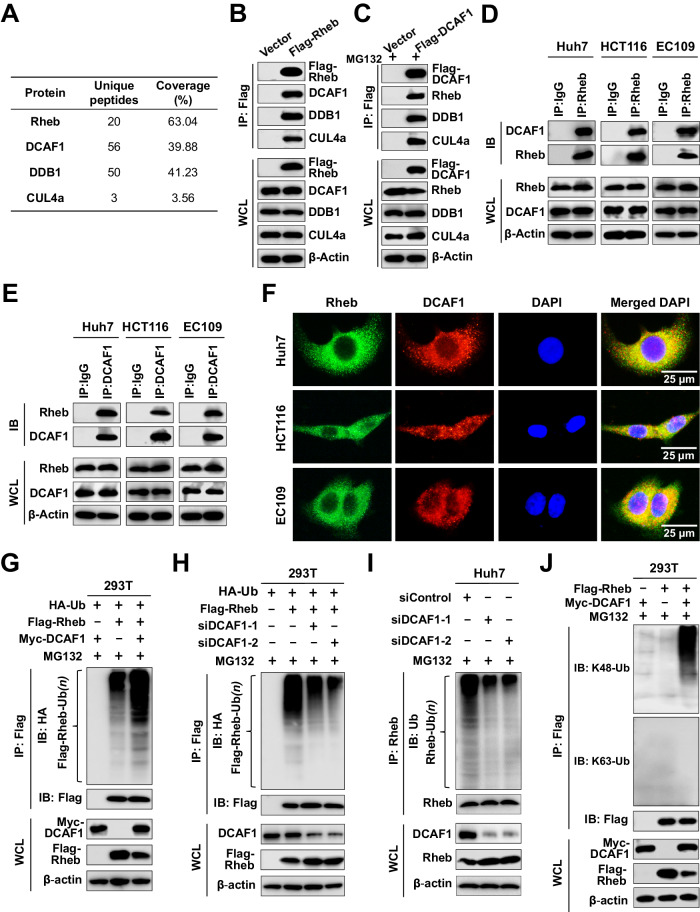
DCAF1 mediates K48-linked polyubiquitination of Rheb. **A** Partial result of mass spectrometry analysis of the Rheb-associated immunoprecipitated complex. **B**, **C** Co-IP of Flag-Rheb and endogenous DCAF or Flag-DCAF1 and endogenous Rheb. 293 T cells transfected with an empty vector or Flag-Rheb (**B**) or Flag-DCAF1 (**C**), followed by IP analysis with anti-Flag and IB analysis as indicated. **D**, **E** IP analysis of endogenous DCAF1 and endogenous Rheb. IB analysis of co-immunoprecipitated endogenous DCAF1 and Rheb proteins using anti-Rheb (**D**) or anti-DCAF1 (**E**) antibody in Huh7, HCT116, and EC109 cells. An unrelated IgG was used as negative control. **F** Co-localization analysis of DCAF1 and Rheb. Huh7, HCT116, and EC109 cells were fixed and immunostained with antibodies against the indicated proteins. Representative images are shown. Scale bars: 25 μm. **G** DCAF1 promotes Rheb ubiquitination. 293T cells were transfected with recombinant plasmids as indicated and treated with 10 µM MG132, and subjected to IP with anti-Flag, followed by IB with anti-HA or anti-Flag. **H**, **I** Knockdown of DCAF1 attenuates Rheb polyubiquitination. **H** 293T cells were transfected with siDCAF1, Flag-Rheb, and HA-Ub as indicated, and subjected to IP with anti-Flag, followed by IB with anti-HA. **I** Huh7 cells were transfected with siDCAF1, and Rheb polyubiquitination was analyzed by IP with anti-Rheb, followed by IB with anti-Ub. **J** DCAF1 elevates the K48-linked, but not the K63-linked, polyubiquitination of Rheb. 293T cells were transfected with recombinant plasmids as indicated, treated with MG132, and subjected to IP with anti-Flag, followed by IB with anti-K48 Ub or anti- K63 Ub. All data were representative of at least three independent experiments (*n* = 3).

To investigate the mechanism underlying the glucose deprivation-induced ubiquitination and degradation of Rheb, mass spectrometry analysis was performed on the Rheb-associated immunoprecipitated complex, which revealed that Rheb was present in association with DCAF1, CUL4a, and DDB1 (Fig. 2A). Reciprocal co-immunoprecipitation (IP) assays in HEK293T (293T) cells confirmed the strong interaction between Rheb, DCAF1, CUL4a, and DDB1 (Fig. 2B, C). The interaction between Rheb and DCAF1 was further validated by IP assays with anti-Rheb antibody (Ab) and immunoblotting (IB) with anti-DCAF1 Ab at endogenous levels in cells (Fig. 2D), which was confirmed by reversing IP with anti-DCAF1 Ab and IB with anti-Rheb Ab (Fig. 2E). Subsequently, the cellular location of DCAF1 and Rheb was determined, with DCAF1 mainly localizing in the cytoplasm and slightly in the nucleus (Supplementary Fig. 2A–D), and Rheb primarily expressed in the cytoplasm (Supplementary Fig. S2E), which was consistent with observations of previous studies [21, 22, 38]. Co-localization analysis revealed that DCAF1 and Rheb co-localized in the cytoplasm (Fig. 2F). These results suggest that DCAF1 interacts with Rheb.

To further examine whether DCAF1 promotes the ubiquitination of Rheb, in vivo ubiquitination experiments were performed. 293T cells were transfected with HA-ubiquitin (Ub), Flag-Rheb, and Myc-DCAF1, and treated with MG132 to prevent protein degradation before performing the ubiquitination assay. Anti-HA IB revealed that overexpressed DCAF1 led to a large increase of polyubiquitinated Rheb (Fig. 2G). Conversely, silencing DCAF1 significantly decreased the polyubiquitination of exogenous and endogenous Rheb (Fig. 2H, I and Supplementary Fig. 2F), indicating that DCAF1 was participated in the polyubiquitination of Rheb. Furthermore, we found that overexpression of DCAF1 elevated the K48-linked, but not K63-linked, polyubiquitination of Rheb (Fig. 2J). DCAF1-mediated Rheb polyubiquitination could be reduced by the transfection of Ub-K48R, Ub mutant with lysine 48 substituted with an arginine, while Ub-K63R not (Supplementary Fig. 2G, H). Collectively, these results prove that DCAF1 interacts with Rheb and regulates K48-linked polyubiquitination of Rheb.

### DCAF1 promotes the ubiquitination and proteasome-dependent degradation of Rheb

**Fig. 3:**
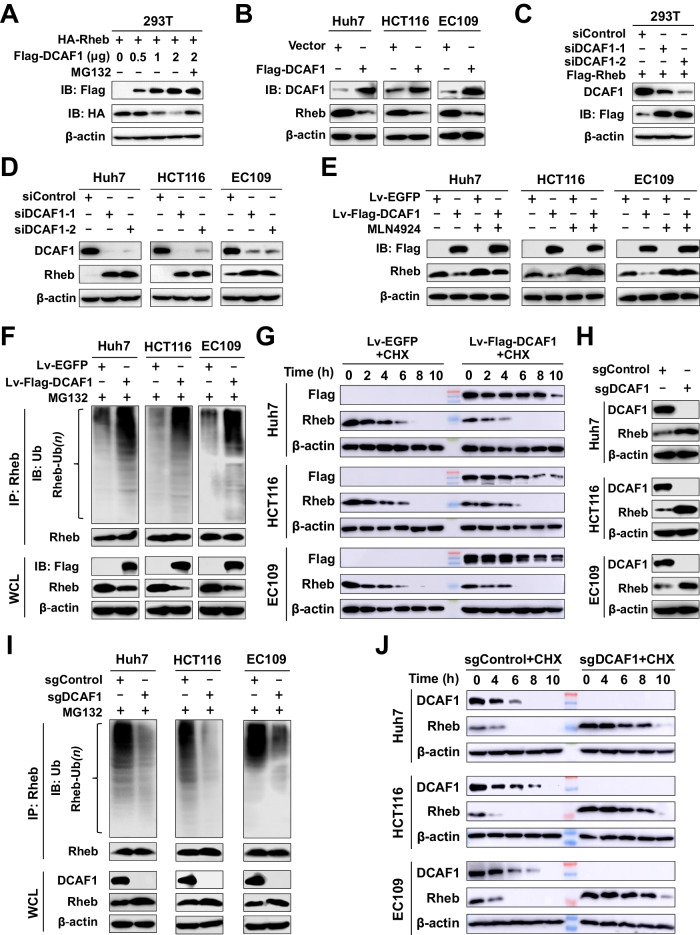
DCAF1 regulates the degradation of Rheb. **A**, **B** Overexpression of DCAF1 reduces the protein level of Rheb. 293T cells were transfected with different amounts of Flag-DCAF1 and same amount of HA-Rheb (**A**) or Huh7, HCT116, and EC109 cells were transfected with vector or Flag-DCAF1 (**B**). **C**, **D** Cells were collected and analyzed by IB as indicated. Knockdown of DCAF1 enhances the protein level of Rheb. 293T cells (**C**) and Huh7, HCT116, and EC109 cells (**D**) were transfected with siDCAF1 with (**C**) or without (**D**) Flag-Rheb, and analyzed by IB as indicated. **E** Treatment with MLN4924 restores the expression of Rheb in cell lines stably overexpressing DCAF1. Cells with stable overexpression of DCAF1 (Fv-Flag-DCAF1) or its counterpart (Lv-EGFP) were treated with MLN4924 or DMSO, and the protein levels of DCAF1 and Rheb were detected by IB using Flag Ab or Rheb Ab. **F** DCAF1 promotes Rheb ubiquitination. Cells with stable overexpression of DCAF1 or its counterpart were treated with 10 µM MG132. Rheb polyubiquitination was detected by IP using Rheb Ab and IB with Ub Ab. **G** Overexpression of DCAF1 decreases Rheb protein stability. Huh7, HCT116, and EC109 cells with stable overexpression of DCAF1 or its counterparts were treated with 100 µg/ml CHX. Cells were harvested at the indicated time points for IB analysis. **H** Silencing DCAF1 using CRISPR-Cas9 system (sgDCAF1) increases the protein level of Rheb. Cell proteins from sgDCAF1 and sgControl were collected and analyzed using IB. **I** DCAF1 depletion attenuates Rheb polyubiquitination. sgDCAF1 and sgControl cells were treated with 10 µM MG132, harvested, and subjected to IP with anti-Rheb, followed by IB with anti-Ub. **J** Silencing DCAF1 increases Rheb protein stability. Huh7, HCT116, and EC109 cells with stable silencing of DCAF1(sgDCAF1) and its counterpart (sgControl) were treated with 100 µg/ml CHX and harvested at the indicated time points for IB analysis. All data were representative of at least three independent experiments (*n* = 3).

The above results showed that DCAF1 could ubiquitinate Rheb. We next tested whether DCAF1 could regulate Rheb protein stability and degradation. For this, 293T cells were co-transfected with the expression plasmids encoding DCAF1 and Rheb. Overexpression of DCAF1 resulted in the downregulation of both endogenous and exogenous Rheb in 293T cells (Fig. 3A and Supplementary Fig. 3A). Preincubation with the proteasome inhibitor MG132 restored DCAF1-induced Rheb reduction (Fig. 3A and Supplementary Fig. 3A), implying that DCAF1 promoted proteasome-dependent degradation of Rheb. Additionally, upregulation of DCAF1 by transfecting Flag-DCAF1 significantly decreased endogenous Rheb protein levels (Fig. 3B). Conversely, knockdown of DCAF1 by siRNA increased the expression of exogenous Rheb in 293T cells (Fig. 3C) and endogenous Rheb in Huh7, HCT116, and EC109 cells (Fig. 3D). Previous reports have revealed that MLN4924, an inhibitor of neddylation, could suppress the activity of Cullin E3 ubiquitin ligase, including Cullin 4 (Cul4) [39]. Therefore, we treated cells with MLN4924 to test the effect of blocking the Cul4a-DDB1-DCAF1 E3 ligase activity on Rheb expression. Results showed that MLN4924 treatment enriched the protein abundance of Rheb in a time- and dose-dependent manner (Supplementary Fig. 3B, C) and enhanced the Rheb protein stability (Supplementary Fig. 3D). Meanwhile, MLN4924 restored DCAF1-induced Rheb reduction (Fig. 3E). Taken together, these findings indicate that DCAF1 decreases Rheb protein level, which is dependent on its ubiquitinase activity.

To confirm the effect of DCAF1 on the ubiquitination and protein stability of Rheb, we generated stable Huh7, HCT116, and EC109 cell lines containing overexpressed DCAF1 (Lv-Flag-DCAF1) using lentiviral systems and cell lines stably silencing DCAF1 (sgDCAF1), using CRISPR-Cas9 genome editing technology. Notably, stable overexpression of DCAF1 decreased the protein level of Rheb (Fig. 3E and Supplementary Fig. 3E), increased the polyubiquitination of endogenous Rheb (Fig. 3F) and shortened the half-life of Rheb protein (Fig. 3G and Supplementary Fig. 3F). Conversely, stable silencing of DCAF1 increased the protein level of Rheb (Fig. 3H), reduced the polyubiquitination of Rheb (Fig. 3I), and significantly prolonged the half-life of Rheb (Fig. 3J and Supplementary Fig. 3G). Therefore, these results suggest that DCAF1 negatively regulates Rheb protein stability by promoting its polyubiquitination and proteasome-dependent degradation.

### DCAF1 negatively regulates mTORC1 activity through Rheb

**Fig. 4:**
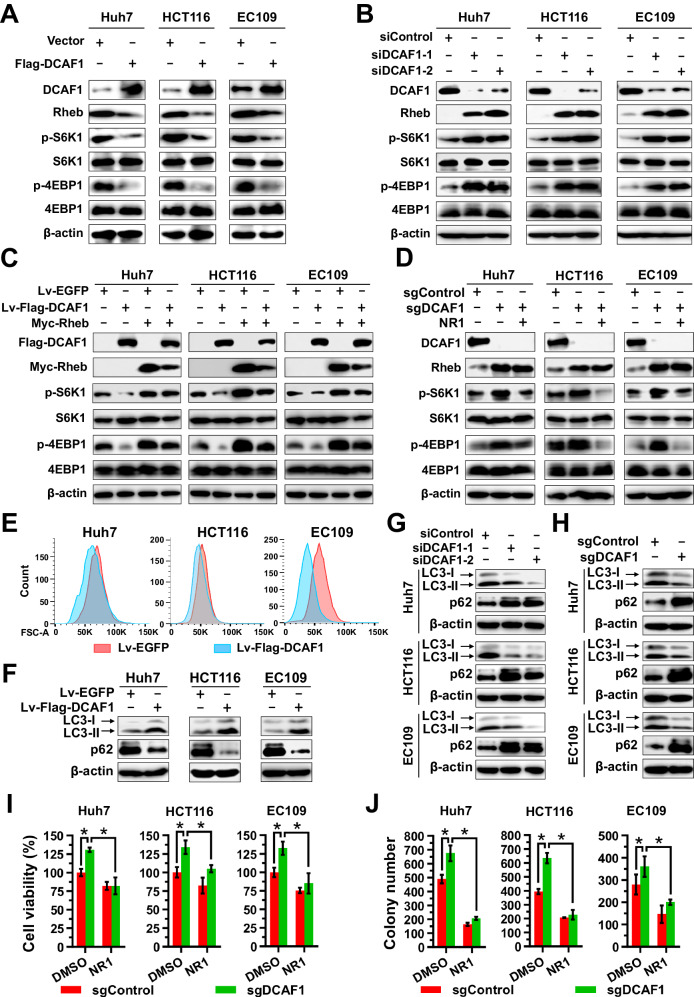
DCAF1 regulates mTORC1 activity through Rheb. **A**, **B** DCAF1 serves as a negative regulator of mTORC1 activity. Huh7, HCT116, and EC109 cells were transfected with Flag-DCAF1 (**A**) or siDCAF1 (**B**) and harvested for IB analysis as indicated. **C**, **D** DCAF1 regulates mTORC1 activity through Rheb. **C** Huh7, HCT116, and EC109 cells stable overexpression of DCAF1 or its counterparts were transfected with Myc-Rheb or left untransfected, and mTORC1 activity was evaluated by IB analysis. **D** Huh7, HCT116, and EC109 cells stable silencing of DCAF1 (sgDCAF1) and its counterparts were treated with NR1, an Rheb inhibitor, and mTORC1 activity was evaluated by IB analysis. **E** Overexpression of DCAF1 reduces cell size. **F**–**H** The expression of DCAF1 affects autophagy. Cell proteins collected from Huh7, HCT116, and EC109 cells with stable overexpression of DCAF1 (**F**), transfected with siDCAF1 (**G**), or stably silencing DCAF1 (**H**) was analyzed by IB as indicated. **I**, **J** DCAF1 affects cell growth through Rheb. Huh7, HCT116, and EC109 cells with stable silencing of DCAF1 (sgDCAF1) and its counterpart (sgControl) were treated with DMSO or NR1 (Rheb inhibitor) for 24 h, and cell viability was detected (**I**). For long term growth, cells were treated with DMSO or NR1 for 10 days, and the number of colonies was counted (**J**). Data are represented as mean ± SEM. *p < 0.05 by Student’s *t* test (**I** and **J**). All data were representative of at least three independent experiments (*n* = 3).

As Rheb is an important positive regulator of mTORC1 pathway [2, 3], we examined the effect of DCAF1 on mTORC1 activity and function. Overexpression of DCAF1 in Huh7, HCT116, and EC109 cells, resulted in decreased levels of Rheb protein and inhibited the mTORC1 activity, as evidenced by reduced phosphorylation of p70 S6 kinase (S6K1) and eIF4E binding protein 1 (4EBP1) (Fig. 4A and Supplementary Fig. 4A). Conversely, DCAF1 knockdown using siRNA or CRISPR-Cas9 system increased Rheb protein levels and upregulated mTORC1 activity (Fig. 4B and Supplementary Fig. 4B). Transfecting Myc-Rheb overexpressing plasmid into the stably overexpressing DCAF1 cells significantly restored the mTORC1 activity (Fig. 4C). Stable silencing of DCAF1 (sgDCAF1) led to upregulation of Rheb and activation of mTORC1, which was effectively suppressed by the knockdown of Rheb (Supplementary Fig. 4C). The activation of mTORC1 by silencing DCAF1 was also attenuated by NR1, a small-molecule inhibitor of Rheb that directly binds Rheb in the switch II domain and inhibits its ability to activate mTORC1 [40] (Fig. 4D). These results indicate that DCAF1 negatively regulates mTORC1 activity through Rheb.

mTORC1 is of great importance for various cellular processes, including cell growth and proliferation, cell survival, and autophagy [4]. Activation of mTORC1 promotes cell growth characterized by an increase in both cell size and cell number [41]. Therefore, we examined the biological significance of DCAF1 in the regulation of cell size, cell growth, and autophagy. Overexpression of DCAF1 reduced cell size (Fig. 4E), which was a phenotypic character of mTORC1 inactivation [42]. Additionally, overexpression of DCAF1 elevated basal autophagy as evidenced by increased LC3II with concomitant suppression of p62, a protein degraded by autophagy (Fig. 4F), while silencing DCAF1 reduced basal autophagy (Fig. 4G, H), which was restored by Rheb inhibitor NR1 (Supplementary Fig. 4D). Moreover, silencing DCAF1 could promote cell viability and colony expansion (Fig. 4I, J and Supplementary Fig. 4E–G), which was abolished by Rheb inhibitor NR1 (Fig. 4I, J and Supplementary Fig. 4H). These data collectively indicate that DCAF1 negatively controls mTORC1 activity and its function through Rheb.

### Glucose deprivation promotes Rheb degradation and inhibits mTORC1 activity *via* transactivating DCAF1

**Fig. 5:**
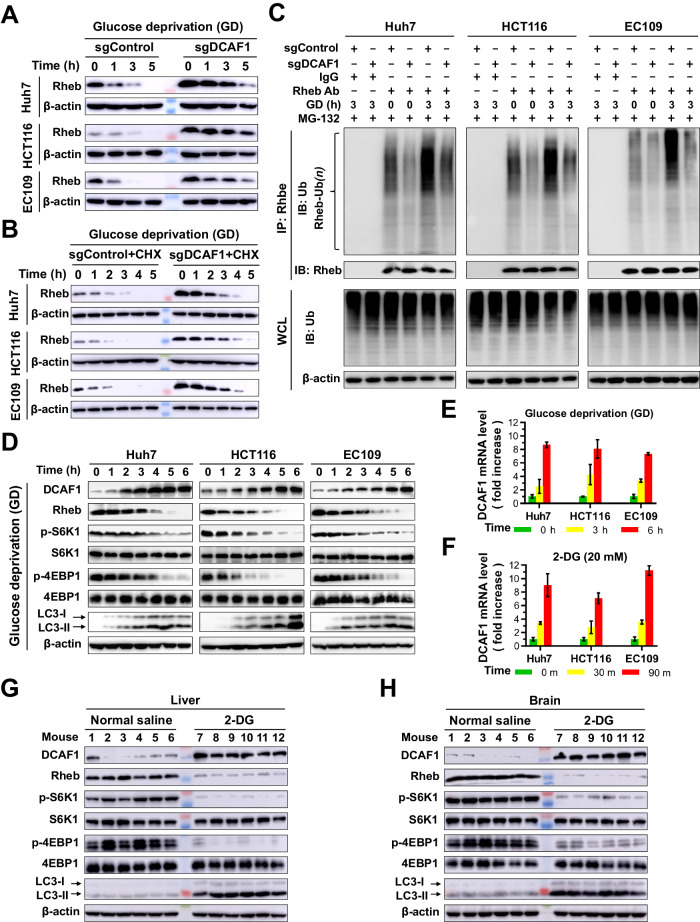
Glucose deprivation transactivates DCAF1, promotes ubiquition-mediated degradation of Rheb, and inhibits mTORC1 activity. **A**, **B** Silencing DCAF1 delays glucose deprivation-induced Rheb degradation and enhances the protein stability of Rheb. Stable sgDCAF1 and sgControl cells were cultured in glucose-free medium, treated without (**A**) or with (**B**) 100 µg/ml CHX, and collected at indicated times for IB analysis. **C** Stable sgDCAF1 cells were cultured with or without glucose-free medium, and the polyubiquitination of Rheb was analyzed by immunoprecipitation. **D** Huh7, HCT116, and EC109 cells were cultured in glucose-free medium and cell protein was collected at indicated times for IB analysis. **E** Huh7, HCT116, and EC109 cells were cultured in glucose-free medium and total RNA was collected at indicated times. The mRNA levels of DCAF1 were analyzed using quantitative-PCR (Q-PCR). **F** Cells were treated with 20 mM 2-DG, and total RNA was collected at indicated times. The DCAF1 mRNA levels were analyzed using Q-PCR. **G**, **H** Effect of 2-DG treatment on the protein level of DCAF1, Rheb, and mTORC1 activity in mouse model. The 6-week-old C57BL6 mice were randomized into two groups and treated with normal saline or 2-DG solution for 3 weeks. The liver (**G**) and brain (**H**) tissues were harvested, and protein expression was evaluated by IB analysis using specific antibodies as indicated. All data were representative of at least three independent experiments (*n* = 3).

We determined the role of DCAF1 in the regulation of Rheb degradation and mTORC1 activity under glucose deprivation conditions. We observed that glucose deprivation resulted in decreased Rheb abundance in sgControl cells in a time-dependent manner (Fig. 5A). Silencing DCAF1 (sgDCAF1) effectively delayed glucose deprivation-induced Rheb downregulation (Fig. 5A) and enhanced Rheb protein stability during glucose deprivation (Fig. 5B and Supplementary Fig. 5I). Further examination found that silencing DCAF1 attenuated glucose deprivation-induced Rheb ubiquitination (Fig. 5C).

To determine the underlying trigger mechanism of DCAF1-mediated Rheb degradation, we examined the protein and mRNA levels of DCAF1 in response to glucose deprivation. Notably, endogenous DCAF1 in both proteins (Fig. 5D and Supplementary Fig. 5A) and mRNA levels (Fig. 5E and Supplementary Fig. 5E) were significantly upregulated when cells were starved by culturing in glucose-free medium or EBSS. This regulation was confirmed by 2-DG treatment (Fig. 5F and Supplementary Fig. 5B). However, serum or amino acid deprivation had no such effect, even when prolonged to 10 h (Supplementary Fig. 5C, D). Meanwhile, the increase of DCAF1 induced by glucose deprivation, EBSS starvation, or 2-DG treatment could be inhibited by CHX, which blocked protein synthesis (Supplementary Fig. 5F–H). Furthermore, glucose deprivation or EBSS starvation resulted in marked accumulation of DCAF1 concomitant with a decrease in Rheb and a drastic impairment in mTORC1 activity, as evidenced by downregulating S6K1 and 4EBP1 phosphorylation (Fig. 5D and Supplementary Fig. 5A). These results were confirmed by 2-DG treatment in vitro (Supplementary Fig. 5B) and in vivo (Fig. 5G, H). 2-DG treatment significantly upregulated DCAF1 with concomitant suppression of Rheb and the inactivation of mTORC1 in mice liver and brain (Supplementary Fig. 5G, H). These results indicate that glucose deprivation transactivates DCAF1, which promotes ubiquitin-proteasomal degradation of Rheb and inhibits mTORC1 activity. These findings imply that when glucose supply is abundant, the DCAF1 level is relatively low or moderate, but it is elevated in response to glucose deficiency.

### DCAF1 enhances glucose deprivation-induced autophagy by suppressing the mTORC1 activity

**Fig. 6:**
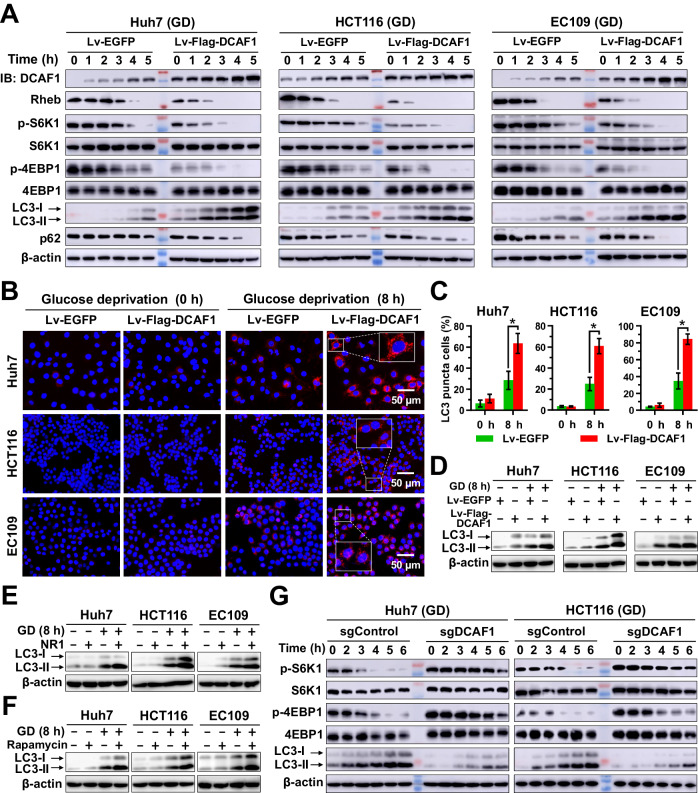
DCAF1 enhances glucose deprivation-induced autophagy. **A**–**D** Stable Lv-Flag-DCAF1 promotes Rheb degradation, inhibits mTORC1 activity, and enhances glucose deprivation-induced autophagy. Huh7, HCT116, and EC109 cells with stably overexpressed DCAF1 were cultured in glucose-free medium for different durations and collected for IB analysis (**A**) or immunofluorescence detection of LC3 (**B**). Scale bars: 50 μm. The statistical analysis for panel **B** was shown in panel **C**. The cells treated as in panel **B** were collected and analyzed by IB (**D**). **E**, **F** Huh7, HCT116, and EC109 cells were cultured with and without glucose-free medium and treated with NR1 (**E**) or rapamycin (**F**). Cell proteins were collected and analyzed. **G** Stable sgDCAF1 or its counterparts were cultured with glucose-free medium. Cell proteins were collected at indicated times and analyzed using IB. Data are represented as mean ± SEM. *p < 0.05 by Student’s *t* test (**C**). All data were representative of at least three independent experiments (*n* = 3).

mTORC1 is a well-known negative regulator of autophagy, whereas nutrient deficiency such as glucose deprivation could induce autophagy *via* inactivating mTORC1 [43, 44]. Previous reports revealed that inhibition of Rheb caused the inactivation of mTORC1 during glucose deprivation [15]. Meanwhile, results in Fig. 5 showed that glucose deprivation or 2-DG treatment in vitro and in vivo caused a time-dependent DCAF1 accumulation and enhanced autophagy by inhibiting the mTORC1 activity (Fig. 5D, G, H and Supplementary Fig. 5A, B). We hypothesize that glucose deprivation-induced upregulation of DCAF1 participates in the stimulation of autophagy. As shown in Fig. 6, overexpression of DCAF1 accelerated glucose deprivation-induced downregulation of Rheb and the inactivation of mTORC1, as well as autophagy induction, as evidenced by increased LC3-II expression and LC3 puncta with a concomitant decrease in p62 abundance (Fig. 6A–D). Moreover, both Rheb inhibitor NR1 and mTORC1 inhibitor rapamycin treatment exhibited similar results as overexpression of DCAF1 and enhanced glucose deprivation-induced autophagy (Fig. 6E, F). Conversely, glucose deprivation inactivated mTORC1 and potentiated autophagy in control cells, and this response was significantly reversed by silencing DCAF1 (Fig. 6G and Supplementary Fig. 6A). These results reveal that DCAF1 is an important positive regulator of autophagy during glucose deprivation by inactivating Rheb-mTORC1 axis.

### DCAF1 promotes cell survival during glucose deprivation

**Fig. 7:**
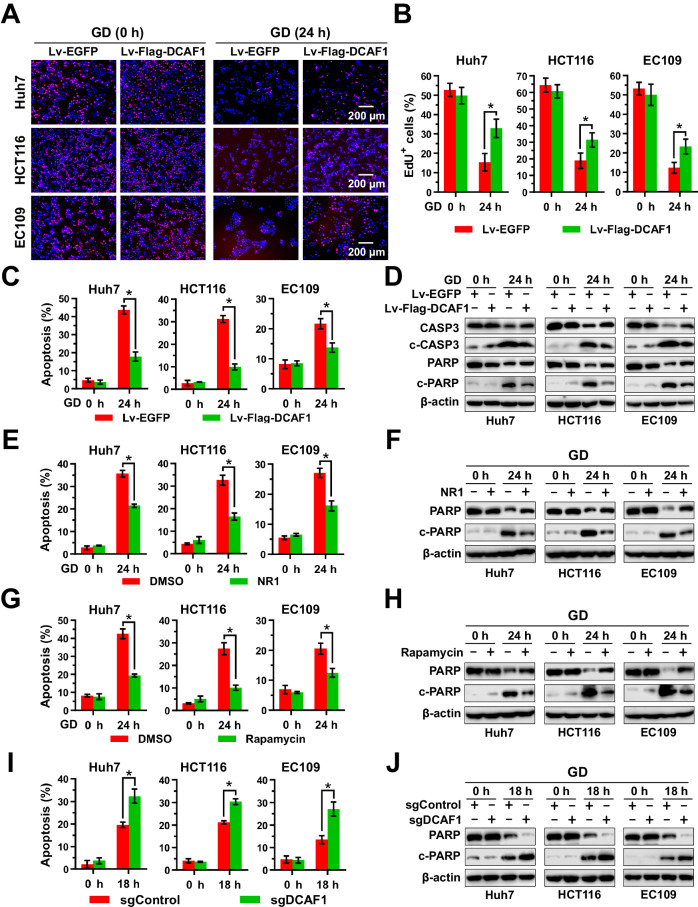
DCAF1 promotes cancer cell survival and protects cancer cell from glucose deprivation-induced cell death. **A**, **B** DCAF1 promotes cell survival under glucose deprivation. Huh7, HCT116, and EC109 cells with stable overexpression of DCAF1 (Lv-Flag-DCAF1) and its counterpart (Lv-EGFP) were cultured in glucose-free medium for 24 h. Cell proliferation was detected by EdU staining, and representative pictures are shown in panel **A**. Scale bars: 200 μm. Statistical analysis is shown in panel **B**. **C**, **D** DCAF1 protects cancer cells from cell death induced by glucose deprivation. Stable Lv-Flag-DCAF1 or control Lv-EGFP cells were cultured in glucose-free medium for 24 h. Cells were collected for apoptotic analysis using Annexin V-APC/7-AAD double staining FACS (**C**) and IB analysis (**D**). **E**–**H** NR1 and rapamycin protect cancer cells from cell death induced by glucose deprivation. Huh7, HCT116, and EC109 cells were cultured in glucose-free medium and treated with NR1 (**E**, **F**) or rapamycin (**G**, **H**) for 24 h. Apoptosis was analyzed using Annexin V-FITC/PI double staining FACS (**E**, **G**) and IB analysis (**F**, **H**). **I**, **J** Silencing DCAF1 promotes glucose deprivation-induced cell death. Stable sgDCAF1 or control sgControl cells were cultured in normal glucose or glucose-free medium for 18 h. Apoptosis was analyzed using Annexin V-APC/7-AAD double staining FACS (**I**) and IB analysis (**J**). Data are represented as mean ± SEM. *p < 0.05 by Student’s *t* test (**B**, **C**, **E**, **G**, and **I**). All data were representative of at least three independent experiments (*n* = 3).

Previous reports revealed that inhibition of mTORC1 and induction of autophagy can facilitate cancer cell survival under low glucose conditions [45, 46]. To investigate the biological function of DCAF1 in glucose deficiency, we investigated its effect on cancer cell survival and cell death during glucose deprivation. Our results found that glucose deprivation suppressed cell proliferation, while overexpression of DCAF1 partially restored it, as indicated by an increase in Edu^+^ cells (Fig. 7A, B). To further validate the protective role of DCAF1 against glucose deprivation-induced cell death, cells with stably overexpressed DCAF1 and their control counterparts were incubated in glucose-free medium, and apoptosis was detected. We observed that Huh7, HCT116, and EC109 cells containing the vector (Lv-EGFP) underwent massive cell death after shifting to glucose-free conditions (Fig. 7C and Supplementary Fig. 7A). IB analysis for apoptotic markers revealed that cleaved caspase 3 and cleaved PARP were accumulated during glucose deprivation (Fig. 7D). In contrast, stable overexpression of DCAF1 (Lv-Flag-DCAF1) significantly reduced glucose deprivation-induced cell death (Fig. 7C, D and Supplementary Fig. 7A), suggesting that DCAF1 can protect cancer cells from glucose deprivation-induced cell death. Furthermore, treatment with Rheb inhibitor NR1 (Fig. 7E, F and Supplementary Fig. 7B) or mTOR inhibitor rapamycin (Fig. 7G, H and Supplementary Fig. 7C) produced similar protective effects. However, stable silencing of DCAF1 had the opposite effect, sensitizing cells to glucose deprivation concomitant with enhanced apoptosis (Fig. 7I, J and Supplementary Fig. 7D), which was partially alleviated by Rheb inhibitor NR1 (Supplementary Fig. 7E). In summary, these results indicate that DCAF1 promotes cell survival and protects cancer cells from apoptosis by inactivating the Rheb-mTORC1 pathway under glucose deprivation conditions.

## Discussion

Glucose deprivation is a common occurrence in solid tumors due to limited blood supply and heightened glucose consumption [47]. Notably, tumor glucose levels are often lower compared to normal tissues [48]. Cancer cells have developed various cellular pathways to overcome glucose deficiency and ensure their survival in such environments [1]. Exploring the mechanism by which cancer cells survive in low glucose environments is crucial for understanding cancer progression and developing novel anti-cancer strategies.

mTORC1 activity is regulated by glucose, growth factors, and amino acids [49, 50]. Recent advancements have shed light on the connection between amino acid, growth factors, and mTORC1 function, leading to the identification of specific sensors [3, 51]. However, the precise regulation of mTORC1 activity by glucose levels remains an intriguing and unresolved issue. AMPK is a well-known glucose sensor that modulates mTORC1 during glucose deprivation by activating TSC2 or inhibiting Raptor [6, 52–58]. Notably, mTORC1 can still be inactivated upon glucose starvation in AMPK knockout cells, indicating that AMPK is not unique in glucose sensing [14] and that other sensors might also participate in the regulation of mTORC1. Ras-related GTPases (RagA-D) have been implicated as additional regulators of mTORC1 during glucose deprivation [6, 59–62]. Furthermore, ubiquitination plays a crucial role in regulating mTORC1 signaling by modulating the ubiquitination levels of mTORC1 complex components and key regulators of the mTORC1 pathway [4, 7, 63–65]. These findings suggest that the regulation of mTORC1 activity in response to glucose levels is diverse and occurs at multiple levels. It’s worth exploring how is mTORC1 activity is controlled by glucose, and which sensors are involved?

Accumulating evidence suggests that Rheb is an important positive regulator of the mTORC1 pathway [2, 3, 9]. Inhibition of Rheb leads to mTORC1 inactivation, while overexpression of Rheb in vitro and in vivo restores mTORC1 activity [10]. Besides the classical AMPK-TSC1/2-Rheb pathway, other pathways such as GAPDH-Rheb and PRAK-Rheb also participate in modulating mTORC1 activity in response to glucose deprivation or energy deletion [14, 15]. Rheb can antagonize FKBP38 to activate mTORC1 after stimulation from growth factor [66] or bind MCRS1 to trigger amino-acid-induced mTORC1 activation [67]. Recent studies have highlighted the crucial role of ubiquitination in regulating Rheb. Amino acid decreased the activity of lysosomal deubiquitinase ATXN3, leading to the accumulation of polyubiquitinated Rheb (Ub-Rheb) and the activation of mTORC1 on the lysosome. The accumulated Ub-Rheb was degraded in both proteasome- and lysosome-dependent manner [16]. During growth factor deficiency, the E3 ubiquitin ligase RNF152 promotes the mono-ubiquitination and inactivation of Rheb, while upon growth factor stimulation, USP4 deubiquitinates and activates Rheb. Mono-ubiquitination of Rheb suppresses mTORC1 activity [17]. Additionally, Harraz et al. reported that E3 ubiquitin ligase Siah1 degraded Rheb and inhibited the mTOR pathway in response to nitric oxide [18]. Heat shock protein 70 has also been reported to enhance proteasome-dependent degradation of Rheb, leading to mTORC1 inactivation [68]. In the context of glucose deprivation, we find that DCAF1 elevates the polyubiquitination of Rheb and promotes its proteasome-dependent degradation, resulting in mTORC1 inactivation. These studies have revealed distinct modes of regulation for the Rheb-mTORC1 pathway in response to different nutrient stresses.

DCAF1 is expressed in various human and mouse tissues, suggesting its conserved and potentially critical role in multicellular organisms [25]. Since its discovery almost 20 years ago [69], significant progress has been made in elucidating the function of DCAF1 in various cellular processes [20–22]. DCAF1 plays a crucial role in cell cycle [23, 24, 70], DNA replication, embryonic development [20, 25] and the onset and development of tumors [22, 26, 27] by modulating specific substrates. Previous studies have reported that Merlin suppressed tumorigenesis by inhibiting CRL4^DCAF1^ activity [22, 27]. DCAF1 serves as an oncogenic signaling and promotes proliferation of Merlin-loss tumor cells [22]. In Merlin-deficient cells, CRL4^DCAF1^ directly ubiquitinylates and destabilizes LATS1/2, leading to YAP activation and tumorigenesis [71]. Notably, growth factor signaling or serum stimulation induces Akt-dependent phosphorylation of Merlin, facilitating its interaction with DCAF1 and promoting its polyubiquitination and proteasome-mediated degradation [72, 73]. Furthermore, Ren et al. reported that inflammation-induced Jak-STAT3 signaling promoted colon cancer development through DCAF1-mediated proteasome degradation of DICER1 [26]. More recently, DCAF1 protein was reported to be overexpressed and positively correlated with poor clinical outcomes in prostate tumors. DCAF1 promotes prostate cancer cell proliferation by downregulating the stability and activity of p53 [21]. In this study, we identified DCAF1 as a novel glucose sensor and demonstrated its negative regulation of the Rheb-mTORC1 pathway. Under normal or glucose-rich conditions, DCAF1 expression is relatively low or moderate, allowing for the accumulation of Rheb and sustained activation of mTORC1. Activated mTORC1 promotes cancer cell proliferation and cell growth. However, under glucose-deprived conditions, rather than amino acid or serum deprivation, DCAF1 is transactivated, leading to the polyubiquitination and proteasome-dependent degradation of Rheb, thereby inactivating mTORC1 and promoting cancer cell survival. These findings are consistent with previous reports indicating that mTORC1 activation promotes cancer cell proliferation under nutrient-rich conditions, but mTORC1 inactivation promotes tumor cells survival under nutrient or energy deficiency [6, 74]. Uncontrolled high mTORC1 activity can result in cell death during energy deprivation [57]. Notably, the mTORC1 inhibitor rapamycin has been shown to promote the proliferation of tumor cells located in poorly vascularized areas of the tumor in a pancreatic cancer mouse model [6]. Moreover, during tumor initiation, mTORC1 functions as an oncogene [75, 76], but in established tumors, when nutrients were limiting, mTORC1 suppression drives tumor cell proliferation [76, 77]. Our findings, in conjunction with previous reports, suggest that DCAF1 may play distinct roles in different stages of cancer progression, which require further investigation. Notably, we observed a significant upregulation of DCAF1 at both the protein and mRNA levels in response to glucose deprivation; however, the underlying mechanism remains to be elucidated. Notably, previous studies reported that the NF2 tumor suppressor gene product, Merlin, functions as an upstream negative regulator of DCAF1 (VprBP) [22]. Additionally, Poulose et al. reported that DCAF1 (VprBP) is regulated by the androgen receptor at the transcript level, and by O-GlcNAc transferase at the protein level [21], providing potential insight for further study.

Mounting evidence indicates that glucose deficiency often leads to cell death in fast-growing cancer cells. To overcome the stress of glucose deprivation and support cell survival, cancer cells have developed various mechanisms [1], including autophagy, to sustain themselves under low glucose conditions [45, 46, 78, 79], and it is often observed in poorly vascularized sites of solid tumors [80]. The regulation of glucose deprivation-induced protective autophagy involves multiple pathways [45, 46, 78, 79, 81, 82], with the mTORC1 complex being a well-known negative regulator of autophagy [53, 83]. In addition to the classical AMPK-mTORC1 pathway [45, 84, 85], other mechanisms have been identified. For instance, HK-II binding to TORC1 suppresses mTORC1 activity and promotes protective autophagy in response to glucose deprivation [8]. Glucose deprivation could induce β-catenin degradation and lead to autophagy [82]. The accumulation of DEPTOR, an mTOR inhibitor, also leads to autophagy as a cellular protective mechanism [4, 7]. In this study, we uncovered that DCAF1-mediated Rheb degradation and mTORC1 inactivation contribute to cancer cell survival under glucose deprivation conditions. It is worth noting that previous studies have also reported that Rheb inhibition during cellular stress triggers adaptive mechanisms such as cell cycle arrest, autophagy, and growth inhibition to play a protective role [10, 86]. For example, Rheb inactivation during glucose deprivation promotes cardiomyocyte survival by activating autophagy [10]. Furthermore, the Rheb/p27 axis, independent of mTORC1, has been suggested to activate autophagy and promote cancer cell survival under serum deprivation [87]. In our study, we observed that transactivated DCAF1 significantly triggered autophagy during glucose deprivation. To our knowledge, this is the first experimental evidence demonstrating the role of DCAF1 as a positive regulator of autophagy in the absence of glucose by suppressing the Rheb-mTORC1 pathway. However, we also observed that DCAF1 only partially rescued glucose deprivation-induced apoptosis and promoted cell survival, suggesting the involvement of other underlying mechanisms.

In summary, our study identified DCAF1 as a novel cellular glucose sensor and demonstrated its involvement in cancer cell survival under glucose deprivation conditions. Mechanistically, glucose deprivation transactivated DCAF1, which promoted K48-linked polyubiquitination and degradation of Rheb, subsequently suppressing mTORC1 activity and inducing autophagy to facilitate cancer cell survival under such conditions. Though the precise Rheb ubiquitination sites mediated by DCAF1 during this process need further investigation, these findings enhance our understanding of the mechanisms underlying cancer cell survival in glucose-deprived microenvironments, contribute to our knowledge of this crucial cellular process, and may offer potential insights for the development of targeted cancer therapies.

## Materials and methods

### Cell culture and reagents

High-glucose DMEM medium (Cat: C3110-0500, Viva Cell) with 10% FBS (BI) was used for culturing the Huh7, HCT116, EC109, and HEK293T (293T) cells. All the cell lines were tested and had no mycoplasma contamination. For nutrient deprivation experiments, cells were cultured in EBSS (Cat: C0214, Beyotime Biotechnology, Jiangsu, China). Glucose deprivation was achieved by washing cells twice with PBS and culturing them in glucose-free DMEM (Cat: PM150274, Procell) containing 10% FBS according to previous reports [46, 82, 88–90]. For amino acid (AA) deprivation, cells were cultured in a handmade DMEM base medium lacking AA (Cat: X054P1, BasalMedia). Serum deprivation experiments were conducted by incubating cells in a DMEM-based medium without serum.

2-Deoxy-D-glucose (2-DG, Beyotime, China, Cat:154-17-6), NR1 (a Rheb inhibitor, AOBIOUS, US, Cat:AOB 33900), Cycloheximide (CHX, Meilunbio, China, Cat:66-81-9), Bafilomycin A1 (Baf A1, Sigma Aldrich, China, Cat:88899-55-2), and MG132 (a proteasome inhibitor, MCE, China, Cat:HY-13259) were purchased from the respective reagent companies mentioned above and dissolved in dimethyl sulfoxide (DMSO) (Cat:D8370, Beijing Solarbio Science & Technology Co., Ltd).

### Construction of stable transfection cell lines

Cell lines stably overexpressed DCAF1 (Lv-Flag-DCAF1) and control cell lines (Lv-EGFP) were generated in Huh7, HCT116, and EC109 cells, using a lentiviral system. Cell lines stably silencing *DCAF1* in Huh7, HCT116, and EC109 (sgDCAF1) and its counterpart sgControl was constructed using CRISPR-Cas9 system. Lentivirus was customized from GeneChem Co., Ltd. (Shanghai, China). Huh7, HCT116, and EC109 cells were incubated with medium which contained the virus and 8 µg/ml polybrene for 24 h; the screening procedure used G418 (Cat: ST081, Beyotime, China).

### Antibodies

Primary antibodies used in this study include anti-Rheb (Cat:sc-271509), anti-Ubiquitin (Ub) (Cat:sc-166553), anti-DCAF1(Cat:sc-376850), anti-DDB1 (Cat:sc-376860), anti-p-S6K (Cat:sc-8416), anti-p-4EBP-1(Cat:sc-293124), anti-CUL4a (Cat:sc-377188) (Santa Cruz Biotechnology, Inc., Texas, USA), anti-Flag (Cat:GNI4110, GNI, Japan), anti-HA (Cat:AF2305, Beyotime Biotechnology, Jiangsu, China), anti-S6K (Cat:A5512), anti-4EBP1 (Cat:A5090), anti-LC3 (Cat:A5202), anti-p62 (Cat:A5180) (Bimake, Shanghai, China), anti-CASP3 (Cat:9662), anti-cleaved CASP3 (c-CASP3) (Cat:9664), anti-total PARP (t-PARP) (Cat:9532), anti-cleaved PARP (c-PARP) (Cat:5625) (Cell Signaling Technology, Inc., Boston, MA), anti-β-actin (Cat:30102ES60, Yeasen Biotechnology Co., Ltd., Shanghai, China), anti-ubiquitin K48 (Cat:R24785), and anti-ubiquitin K63 (Cat:R27470) (Chengdu Zhengneng Biotechnology Co., Ltd., Chengdu, China). Secondary antibodies include goat anti-mouse IgG (Cat: ZB-2301) and goat anti-rabbit IgG (Cat: ZB-2305) (Beijing Zhongshan Jinqiao Biotechnology Co., Ltd., Beijing, China), normal mouse IgG (Cat: sc-2025, Santa Cruz Biotechnology, Inc., Texas) and EasyBlot anti-mouse IgG (HRP) (Cat: GTX221667-01, Genetex, California, USA) were also employed. All the original western blotting data are in the Supplementary Materials.

### Immunoprecipitation

After lysing on ice for 30 min using NP-40 buffer (Cat:P0013F, Beyotime, China), the supernatants were collected after centrifuging the cell lysates for 15 min at 12,000 rpm, 4 °C and incubated with the appropriate antibodies as indicated, and rotated with agarose beads (dilution: 1:20, Cat:sc-2003, Santa Cruz Biotechnology) at 4 °C overnight. The agarose bead-containing compounds were rinsed three times with NP-40 buffer. Whole cell lysates and immunoprecipitated specimens were analyzed by IB.

For transfection-based experiments, 293T cells were transfected with Flag-tagged plasmid or siRNA using lipo8000 (Cat: C0533, Beyotime, China) for 48 h and then treated as described above.

### In vivo ubiquitination assay

After transfecting with HA-Ub, Flag-RHEB, Myc-DCAF1 or siDCAF1 for 48 h, 293T cells were collected and lysed with RIPA lysis buffer (Cat:P0013C, Beyotime, China) containing 1% SDS and EDTA-free protease inhibitors (Cat:Roche.11873580001, Roche). Then cell lysates were centrifuged and the supernatants were incubated with the appropriate antibodies as indicated in the figure legends and rotated at 4 °C overnight. The next day, immunoprecipitates were washed and analyzed as described in immunoprecipitation assay.

### Plasmids construction and transfection

Flag-DCAF1, Flag-Rheb and Myc-DCAF1 were constructed for transfection. HA-ubiquitin was kindly gifted from Lijun Jia (Shanghai University of Traditional Chinese Medicine, Shanghai, China). HA-ubiquitin (K48) (Cat: HH-gene-488), HA-ubiquitin (K48R) (Cat: HH-gene-041) and HA-ubiquitin (K63R) (Cat: HH-gene-042) were purchased from HedgehogBio Co., Ltd. (Shanghai, China).

### Sequences of siRNA

siRNAs were synthesized by Genepharma Co., Ltd. (Shanghai, China). The detailed sequences are as follows: siDCAF1-1: UCACAGAGUAUCUUAGAGA; siDCAF1-2: GAUGGCGGAUGCUUUGAUA. siRheb-1: GGUGAUCAGUUAUGAAGAA; siRheb-2: GGCAAGAUGAAUAUUCUAU; siRheb-3: GACGAUUCAAUUUGUUGAA; siRheb-4: CAAGGCAAGUCUUCAUGCU; siControl: UUCUCCGAACGUGUCACGU.

### RNA extraction and quantification

RNA extraction was performed using the Ultrapure RNA Kit (Beijing ComWin Biotech Co., Ltd., Beijing, China). RNA (1.0 mg) was purified and reverse transcribed using the PrimeScript RT Master (Takara Biomedical Technology (Beijing) Co., Ltd., Beijing, China). After that, cDNA was quantified by real-time quantitative PCR using SYBR Green Real-Time PCR Master Mixes (Applied Biosystems, Foster City, CA, USA). The mRNA abundance was normalized to the amount of GAPDH in each sample. The primer sequences used are as follows: DCAF1: forward, 5´- GCGGATGCTTTGATAGGCACCT-3´, reverse, 5´-ACAGCAGGTGAAGCCACTCTCA-3´; Rheb: forward, 5´-CTATCTTTCCTCAGACATACTCCA-3´, reverse, 5´-CACCATATCCAACAATTTGCCATG-3´; GAPDH: forward, 5´-GTCTCCTCTGACTTCAACAGCG-3´, reverse, 5´- ACCACCCTGTTGCTGTAGCCAA-3´.

### Real-time monitoring of cell growth

Cells were added into E-Plate 16 plates (ACEA Biosciences) at an amount of 5000 cells per well and grown at 37 °C with 5% CO_2_. Impedance measurements were performed every 10 min throughout the experiment using the xCELLigence RTCA DP instrument. The measurements were displayed as Cell Index, which provides real time information on cell viability.

### Co-localization analysis

Huh7, HCT116, and EC109 cells were cultured on a glass-bottom-dish. The cells were fixed with methanol and blocked with 5% BSA for 30 min and 2 h, respectively, at room temperature. Then, primary antibodies, as indicated, were added, and incubated at 4 °C. The next day, cells were rinsed and incubated with secondary antibodies: Alexa Fluor^®^ 488 Goat Anti-Rabbit IgG (H + L) (green) (Cat: A0423) or Cy3-labeled Goat Anti-Rabbit IgG (H + L) (red) (dilution: 1:500) (Cat: A0516). Two hours later, DAPI (blue) (2 μg/ml) (Cat: C1002, Beyotime, China) was added to stain the nuclei. Images were taken using fluorescence microscopy (magnification: 400×; Nikon, Nikon Inc., Tokyo, Japan).

### LC3 Immunofluorescence staining

Huh7, HCT116, and EC109 cells stably overexpressing DCAF1 were seeded on glass-bottom dishes. After 8 h of glucose deprivation, cells were fixed and blocked as described above; then, LC3 primary antibody and Cy3-labeled Goat Anti-Rabbit IgG (H + L) secondary antibody (red) were added. DAPI (blue) was used for dyeing the nuclei. Images were captured using fluorescence microscopy (magnification: 200×; Nikon, Nikon Inc., Tokyo, Japan).

### 5-Ethynyl-2’-deoxyuridine (EdU) proliferation assay

The EdU assay was carried out using a BeyoClick™ EdU-555 Kit (Beyotime, China). Huh7, HCT116, and EC109 cells with stably overexpressed DCAF1 were cultured in glucose-free DMEM (Cat: PM150274, Procell) containing 10% FBS for 0 or 20 h. Subsequently, the cells were incubated in EdU working solution (1:500) at 37 °C for 2 h. The nuclei were stained with Hoechst 33342. Images were captured using fluorescence microscopy (magnification: 100×; Nikon, Nikon Inc., Tokyo, Japan).

### Detection of apoptosis

Huh7, HCT116, and EC109 cells with stably overexpressed DCAF1 were grown in normal DMEM or glucose-free DMEM. Cells were collected as indicated, and apoptosis was detected using the Annexin V-APC/7-AAD Apoptosis kit (Tianjin Sungene Biotech Co. Ltd., China). Cells distributed in the upper and lower right quadrants were defined as apoptotic cells.

### Mouse model

Six-week-old C57BL6 mice were randomized into two groups with six mice in each group. The mice were intraperitoneally injected with normal saline or 2-DG solution (200 mg/kg) twice a week, on a 1-day-on/3-days-off schedule for 3 weeks. Liver and brain tissues were collected, and protein abundance was detected by IB assay. Animal experiments were performed according to the animal protocols approved by the Institutional Animal Care and Use Committee of Zhengzhou University.

### Statistical analysis

GraphPad Prism5 (GraphPad Software, Inc., La Jolla, CA, USA) and *t* test was used for statistically assessing the significance of differences between two groups. A *p*-value of <0.05 (* in the figures) means statistically significant. All data were representative of at least three independent experiments (*n* = 3).

### Supplementary information

SUPPLEMENTAL MATERIAL

Original Data

## Data Availability

The authors declare that all data that support the findings of this study are available within the paper and supplementary files.
