# Supplementary material for: Glucose deprivation triggers DCAF1-mediated inactivation of Rheb-mTORC1 and promotes cancer cell survival
Source: Cell Death Dis. 2024 Jun 11;15(6):409. doi: 10.1038/s41419-024-06808-1 (PMC11166663; doi:10.1038/s41419-024-06808-1)
Supplement: Supplementary file 1 — SUPPLEMENTAL MATERIAL [file 41419_2024_6808_MOESM1_ESM.pdf]

Figure S1

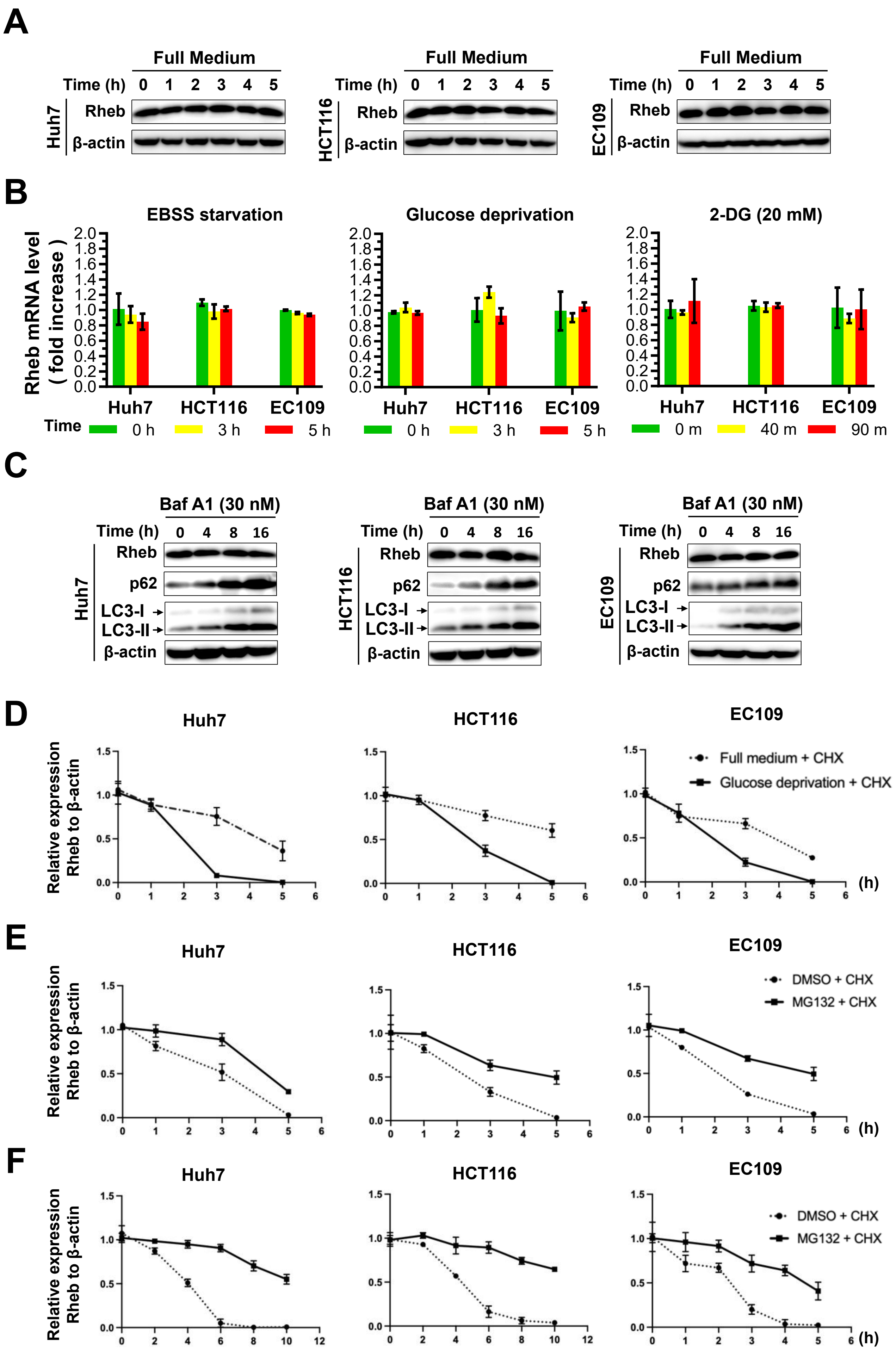

A
Full Medium
Time (h) 0 1 2 3 4 5
Huh7
HCT116
EC109
Rheb
β-actin
B
EBSS starvation
Glucose deprivation
2-DG (20 mM)
Rheb mRNA level (fold increase)
Huh7 HCT116 EC109
Time 0 h 3 h 5 h
0 m 40 m 90 m
C
Baf A1 (30 nM)
Time (h) 0 4 8 16
Rheb
p62
LC3-I
LC3-II
β-actin
D
Huh7
HCT116
EC109
Relative expression Rheb to β-actin
Full medium + CHX
Glucose deprivation + CHX
(h)
E
DMSO + CHX
MG132 + CHX
F

Figure S1 Glucose deprivation induces ubiquitin-mediated degradation of Rheb, related to Figure 1. (A) Huh7, HCT116, and EC109 cells were grown in full medium (DMEM high-glucose medium with 10%FBS) and harvested at the indicated time points. The protein level of Rheb was analyzed. (B) Cells were grown in EBSS, glucose-free medium, or treated with 2-DG for different times. The Rheb mRNA levels were analyzed by quantitative-PCR. (C) Cells were treated with Baf A1 (30 nM) and harvested at the indicated time points. The protein levels of Rheb, p62, and LC3 were analyzed. (D) Quantification of Rheb expression in Fig. 1D. (E) Quantification of Rheb expression in Fig. 1E. (F) Quantification of Rheb expression in Fig. 1J. All data were representative of at least three independent experiments (n=3).

Figure S2

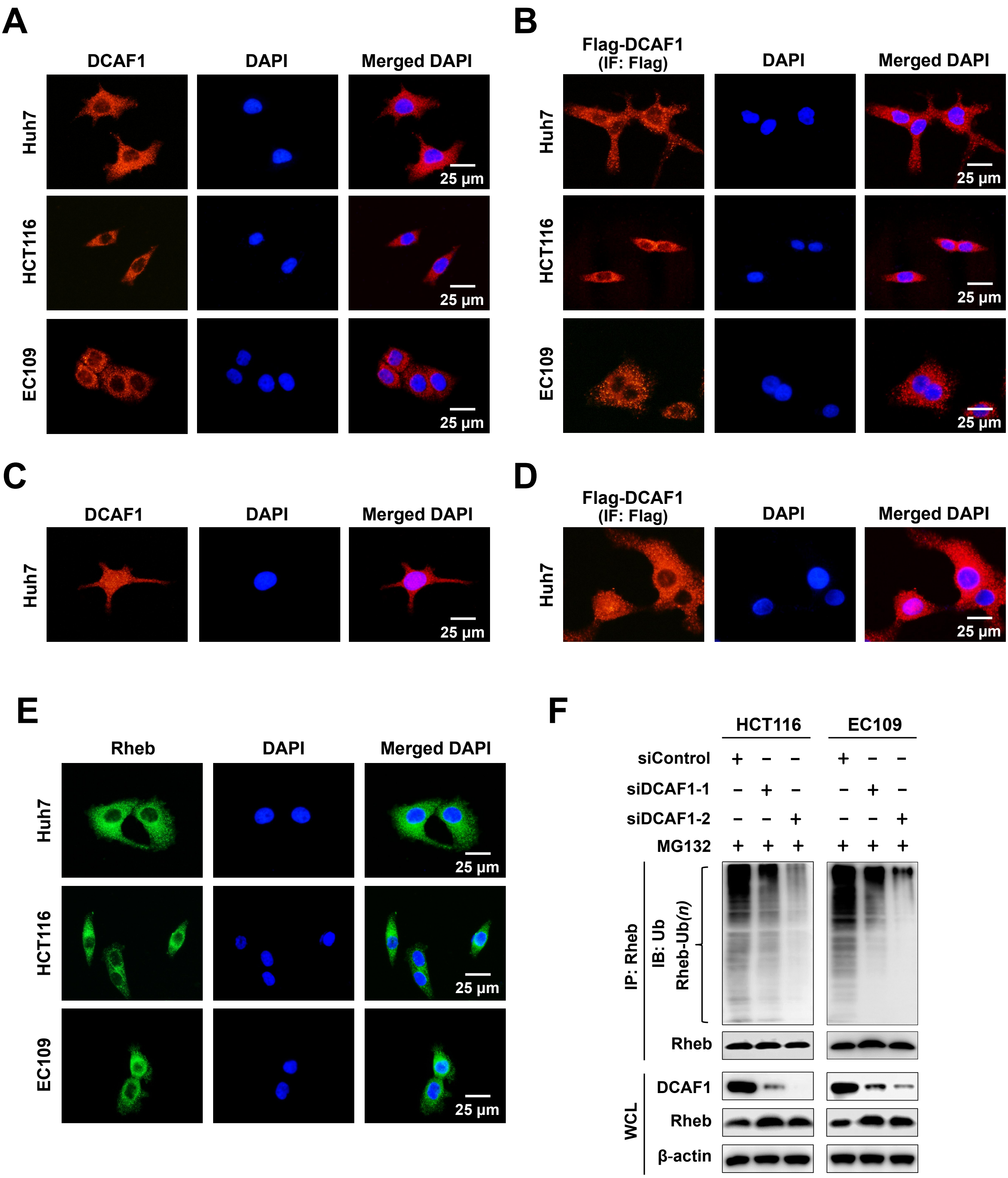

A
DCAF1
DAPI
Merged DAPI
Huh7
HCT116
EC109
25 μm
B
Flag-DCAF1 (IF: Flag)
DAPI
Merged DAPI
Huh7
HCT116
EC109
25 μm
C
DCAF1
DAPI
Merged DAPI
Huh7
25 μm
D
Flag-DCAF1 (IF: Flag)
DAPI
Merged DAPI
Huh7
25 μm
E
Rheb
DAPI
Merged DAPI
Huh7
HCT116
EC109
25 μm
F
HCT116
EC109
siControl
siDCAF1-1
siDCAF1-2
MG132
IP: Rheb
IB: Ub
Rheb-Ub(n)
Rheb
WCL
DCAF1
Rheb
β-actin

Figure S2

G

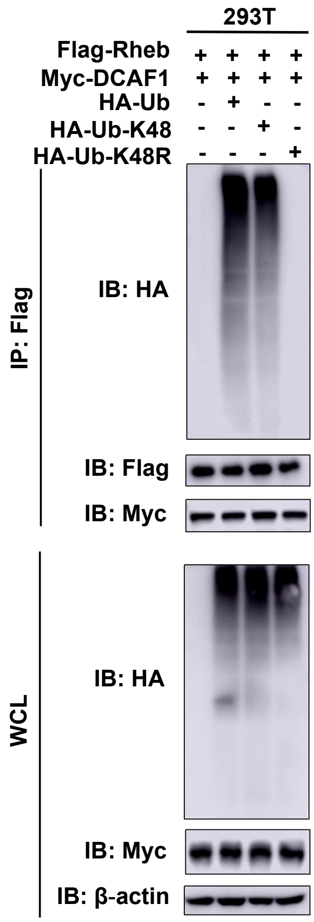
293T
Flag-Rheb + + + +
Myc-DCAF1 + + + +
HA-Ub - + - -
HA-Ub-K48 - - + -
HA-Ub-K48R - - - +
IP: Flag
IB: HA
IB: Flag
IB: Myc
WCL
IB: HA
IB: Myc
IB: β-actin

H

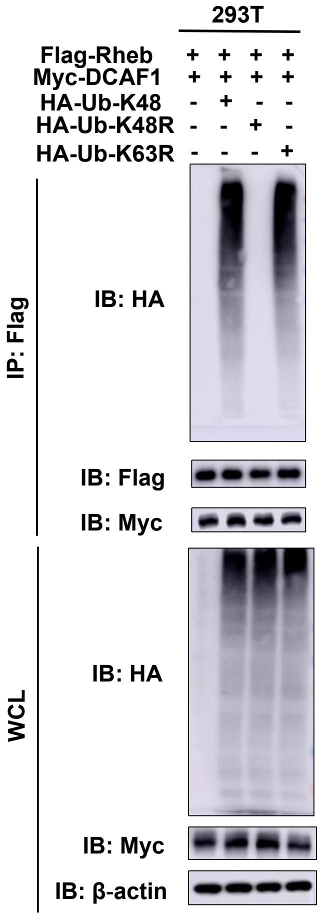
293T
Flag-Rheb + + + +
Myc-DCAF1 + + + +
HA-Ub-K48 - + - -
HA-Ub-K48R - - + -
HA-Ub-K63R - - - +
IP: Flag
IB: HA
IB: Flag
IB: Myc
WCL
IB: HA
IB: Myc
IB: β-actin

Figure S2 DCAF1 mediates K48-linked polyubiquitination of Rheb, related to Figure 2. (A-E) Localization of DCAF1 and Rheb. Huh7, HCT116, and EC109 cells were fixed and stained with antibodies against the indicated proteins. Representative images are shown. Scale bars: 25 μm. (F) HCT116 and EC109 cells were transfected with siDCAF1, and Rheb polyubiquitination was analyzed by IP with anti-Rheb, followed by IB analysis with anti-Ub. (G-H) DCAF1 elevates the K48-linked, but not the K63-linked, polyubiquitination of Rheb. 293T cells were transfected with recombinant plasmids as indicated, treated with MG132, and subjected to IP with anti-Flag, followed by IB with antibodies as indicated. All data were representative of at least three independent experiments (n=3).

Figure S3

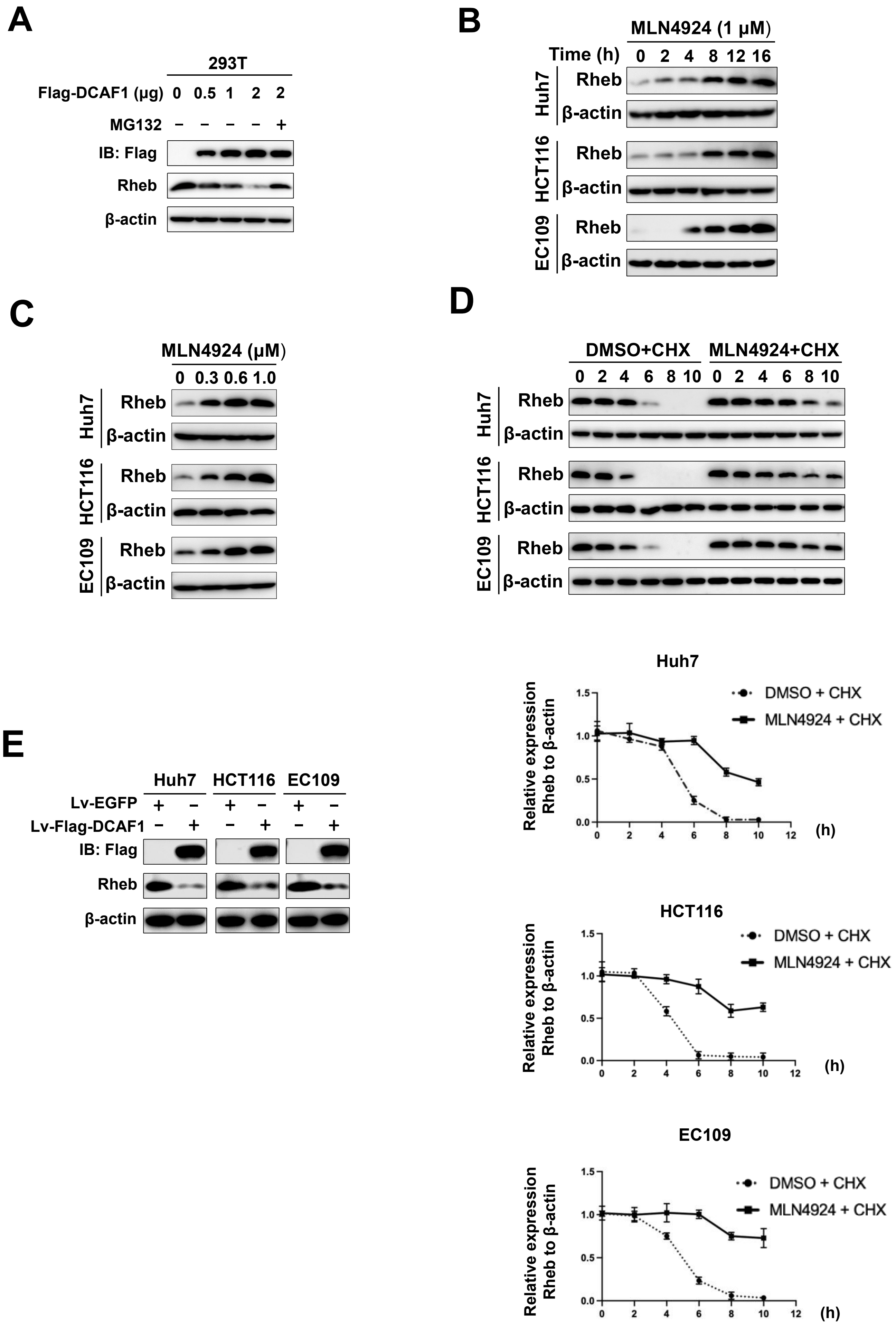

A
293T
Flag-DCAF1 (μg) 0 0.5 1 2 2
MG132 – – – – +
IB: Flag
Rheb
β-actin
B
MLN4924 (1 μM)
Time (h) 0 2 4 8 12 16
Huh7
HCT116
EC109
Rheb
β-actin
C
MLN4924 (μM)
0 0.3 0.6 1.0
D
DMSO+CHX
MLN4924+CHX
0 2 4 6 8 10 0 2 4 6 8 10
E
Huh7
HCT116
EC109
Lv-EGFP
Lv-Flag-DCAF1
Relative expression Rheb to β-actin
DMSO + CHX
MLN4924 + CHX
(h)

Figure S3

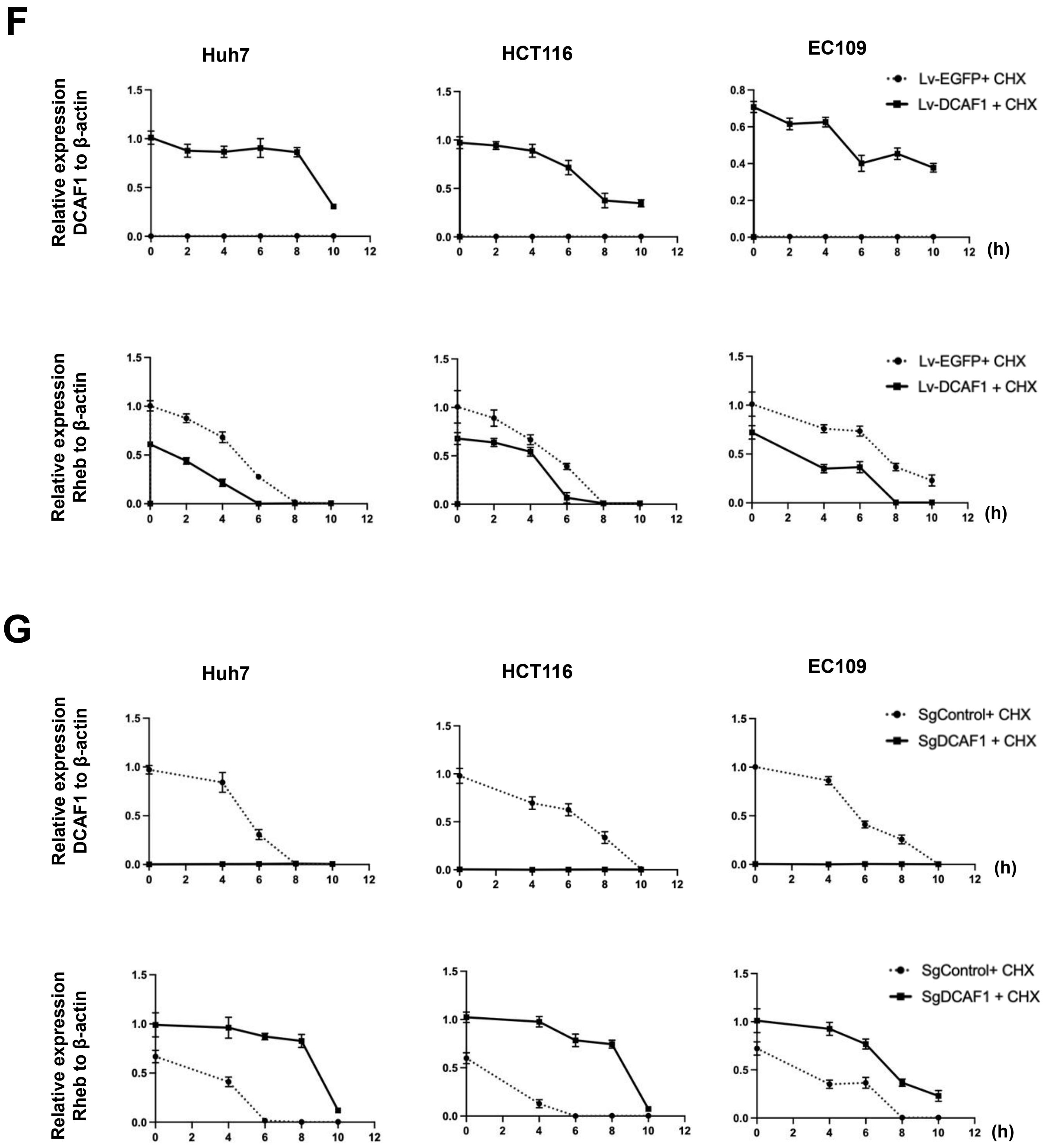

F
Huh7
HCT116
EC109
Relative expression DCAF1 to β-actin
Relative expression Rheb to β-actin
Lv-EGFP+ CHX
Lv-DCAF1 + CHX
(h)
G
SgControl+ CHX
SgDCAF1 + CHX

Figure S3 DCAF1 regulates the degradation of Rheb, related to Figure 3. (A) 293T cells were transfected with different amounts of Flag-DCAF1 and treated with or without MG132. Cell protein was analyzed by IB using antibodies as indicated. (B-C) The effect of MLN4924 on the protein level of Rheb. Huh7, HCT116, and EC109 cells were treated with MLN4924 (1 μM) for indicated time (B) or with different concentration of MLN4924 for 48 h (C), and cell lysates were analyzed by IB as indicated. (D) The effect of MLN4924 on Rheb stability. Huh7, HCT116, and EC109 cells were treated with 100 μg/ml CHX+DMSO or CHX+1μM MLN4924. Cells were harvested at the indicated time for IB analysis in the upper panel. Quantification of Rheb expression in the lower panel. (E) Huh7, HCT116, and EC109 cells stably overexpressing DCAF1 (Lv-Flag-DCAF1) or its counterpart (Lv-EGFP) were harvested and analyzed by IB as indicated. (F) Quantification of DCAF1 and Rheb expression in Fig. 3G. (G) Quantification of DCAF1 and Rheb expression in Fig. 3J. All data were representative of at least three independent experiments (n=3).

Figure S4

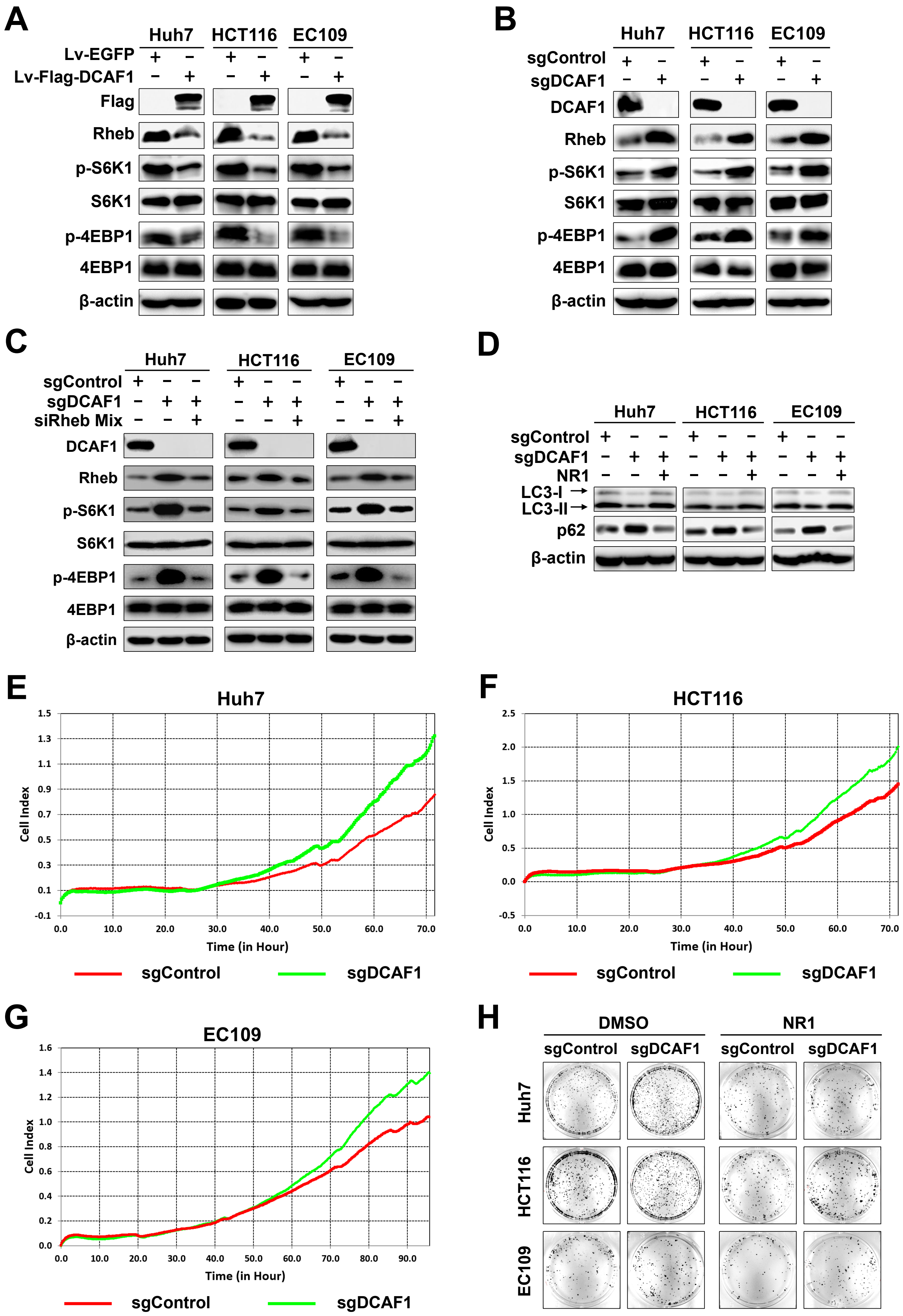

A
Huh7
HCT116
EC109
Lv-EGFP
Lv-Flag-DCAF1
Flag
Rheb
p-S6K1
S6K1
p-4EBP1
4EBP1
β-actin
B
sgControl
sgDCAF1
DCAF1
C
siRheb Mix
D
NR1
LC3-I
LC3-II
p62
E
Cell Index
Time (in Hour)
sgControl
sgDCAF1
F
G
H
DMSO

Figure S4 DCAF1 regulates mTORC1 activity through Rheb, related to Figure 4. (A-B) DCAF1 serves as a negative regulator of mTORC1 activity. Cell protein collected from Huh7, HCT116, and EC109 cells stably overexpressing DCAF1 (A) or silencing DCAF1 (B) was analyzed using IB as indicated. (C-D) DCAF1 regulates mTORC1 activity and autophagy through Rheb. Huh7, HCT116, and EC109 cells stably silencing DCAF1 were transfected with siRheb mix (C) or treated with NR1 (D), Rheb inhibitor, and analyzed using IB as indicated. (E-G) xCELLigence analysis of sgDCAF1 and its counterpart. Cell viability was measured every 10 min for 70 h using the xCELLigence system (Real-Time Cell Analyzer; Roche Applied Science). The results were consistent in three independent experiments. (H) DCAF1 regulates colony expansion through Rheb. sgDCAF1 and its counterpart sgControl were treated with DMSO or NR1. Colons were captured and representative pictures were shown. All data were representative of at least three independent experiments (n=3).

Figure S5

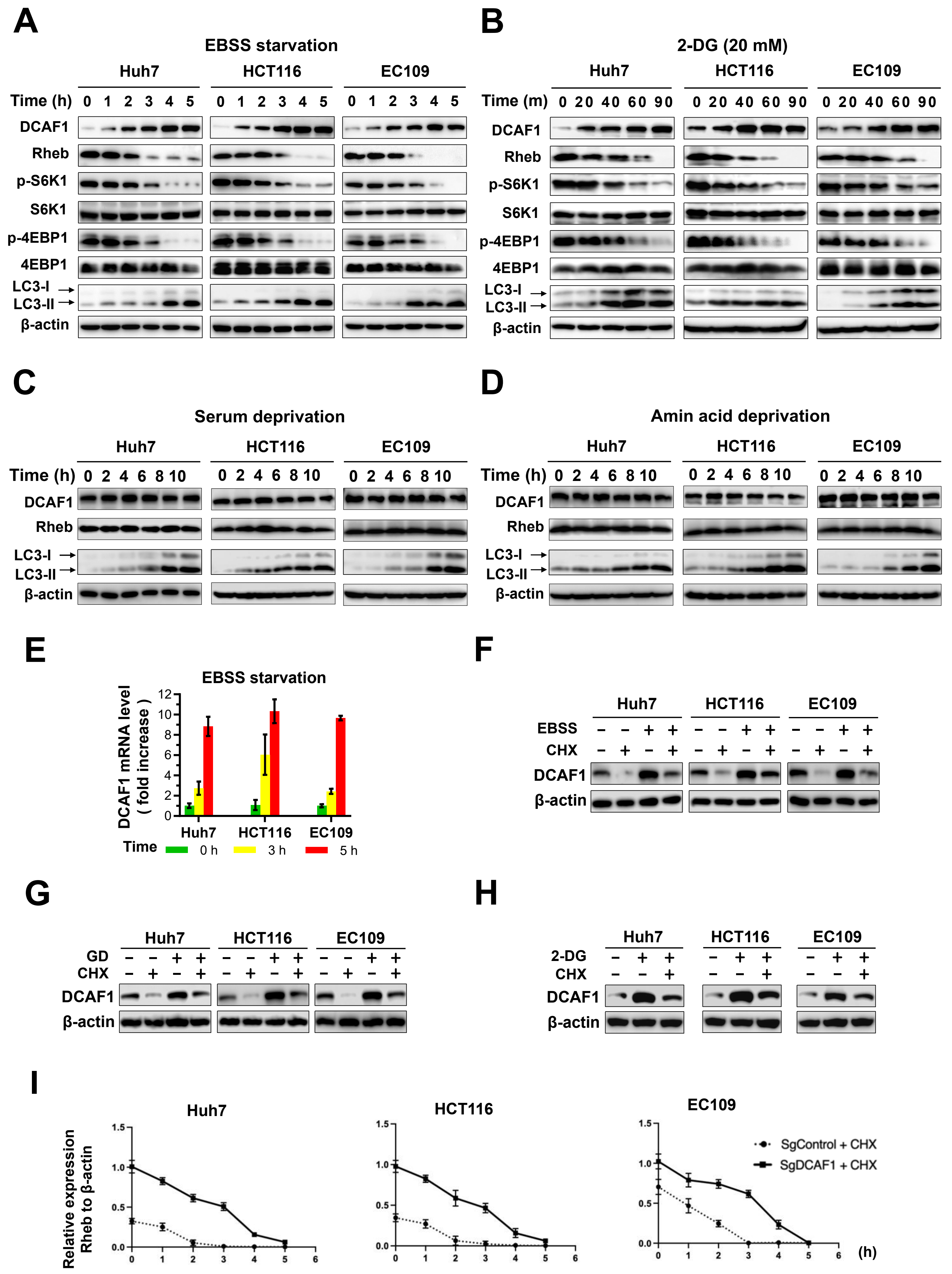
A
EBSS starvation
Huh7
HCT116
EC109
Time (h)
DCAF1
Rheb
p-S6K1
S6K1
p-4EBP1
4EBP1
LC3-I
LC3-II
β-actin
B
2-DG (20 mM)
Time (m)
C
Serum deprivation
D
Amin acid deprivation
E
EBSS starvation
DCAF1 mRNA level (fold increase)
Time
0 h
3 h
5 h
F
EBSS
CHX
G
GD
H
2-DG
I
Relative expression Rheb to β-actin
SgControl + CHX
SgDCAF1 + CHX
(h)

Figure S5 Glucose deprivation transactivates DCAF1, promotes ubiquition-mediated degradation of Rheb, and inhibits mTORC1 activity, related to Figure 5. (A) Huh7, HCT116, and EC109 cells were cultured in EBSS and collected at indicated times for IB analysis. (B) Cells were treated with 20 mM 2-DG and collected at indicated times for IB analysis. (C-D) Huh7, HCT116, and EC109 cells were cultured in serum-free medium (C), or amino acid-free medium (D), and collected at indicated times for IB analysis. (E) Cells were cultured in EBSS, and total RNA was collected at indicated times. The DCAF1 mRNA levels were analyzed using quantitative-PCR. (F-H) Cells were cultured with and without EBSS (F), with or without glucose-free medium (G) and treated with DMSO or CHX (100 μg/ml), or Cells were treated with 2-DG (20 mM) or CHX (100 μg/ml) as indicated (H). Cell proteins were collected and the protein level of DCAF1 was analyzed using IB. (I) Quantification of and Rheb expression in Fig. 5B. Data are represented as mean ± SEM. All data were representative of at least three independent experiments (n=3).

Figure S6

A

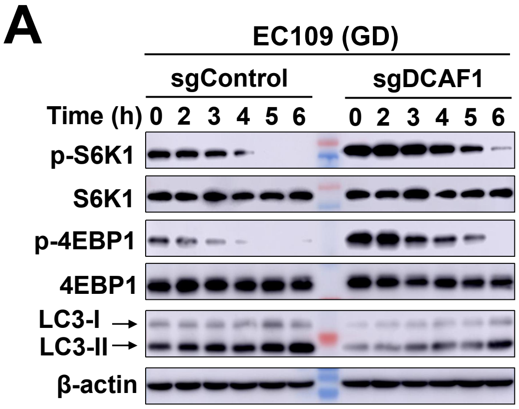

EC109 (GD)
sgControl
sgDCAF1
Time (h) 0 2 3 4 5 6 0 2 3 4 5 6
p-S6K1
S6K1
p-4EBP1
4EBP1
LC3-I
LC3-II
β-actin

Figure S6 DCAF1 enhances glucose deprivation-induced autophagy, related to Figure 6. Stable EC109 expressing sgDCAF1 or its counterparts were cultured in glucose-free medium. Cell proteins were collected at indicated times and analyzed by IB. All data were representative of at least three independent experiments (n=3).

Figure S7

A

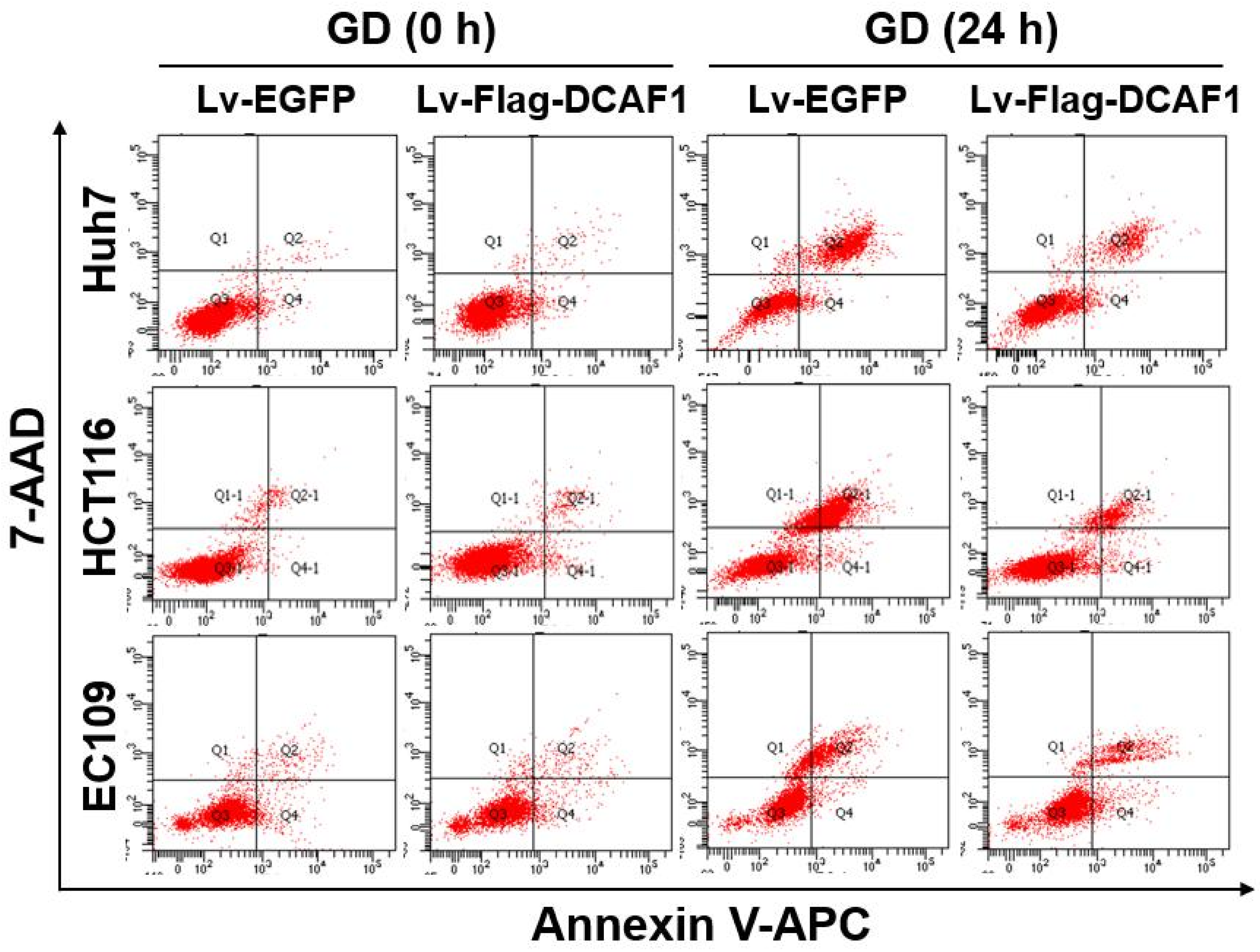

B

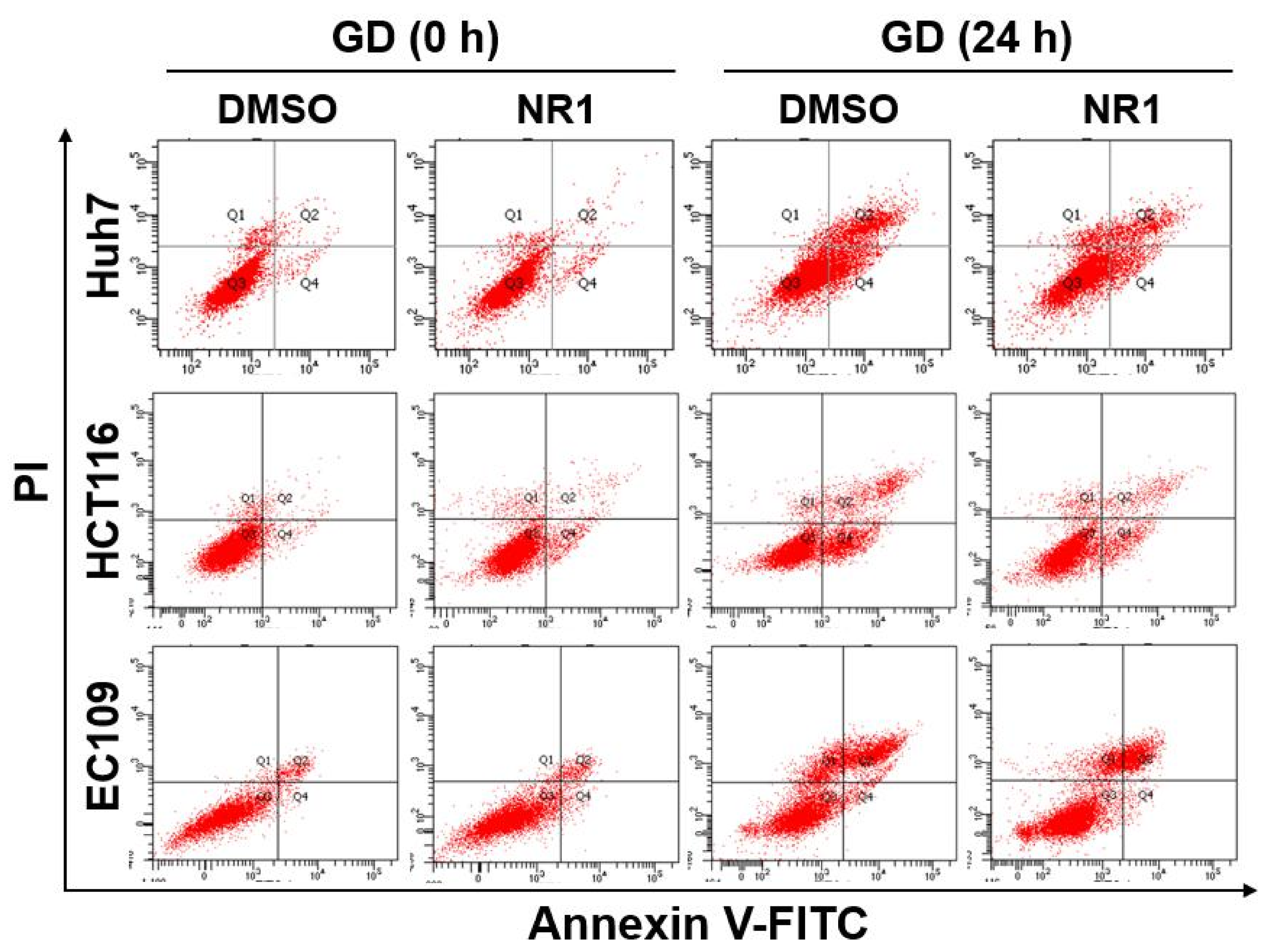

C

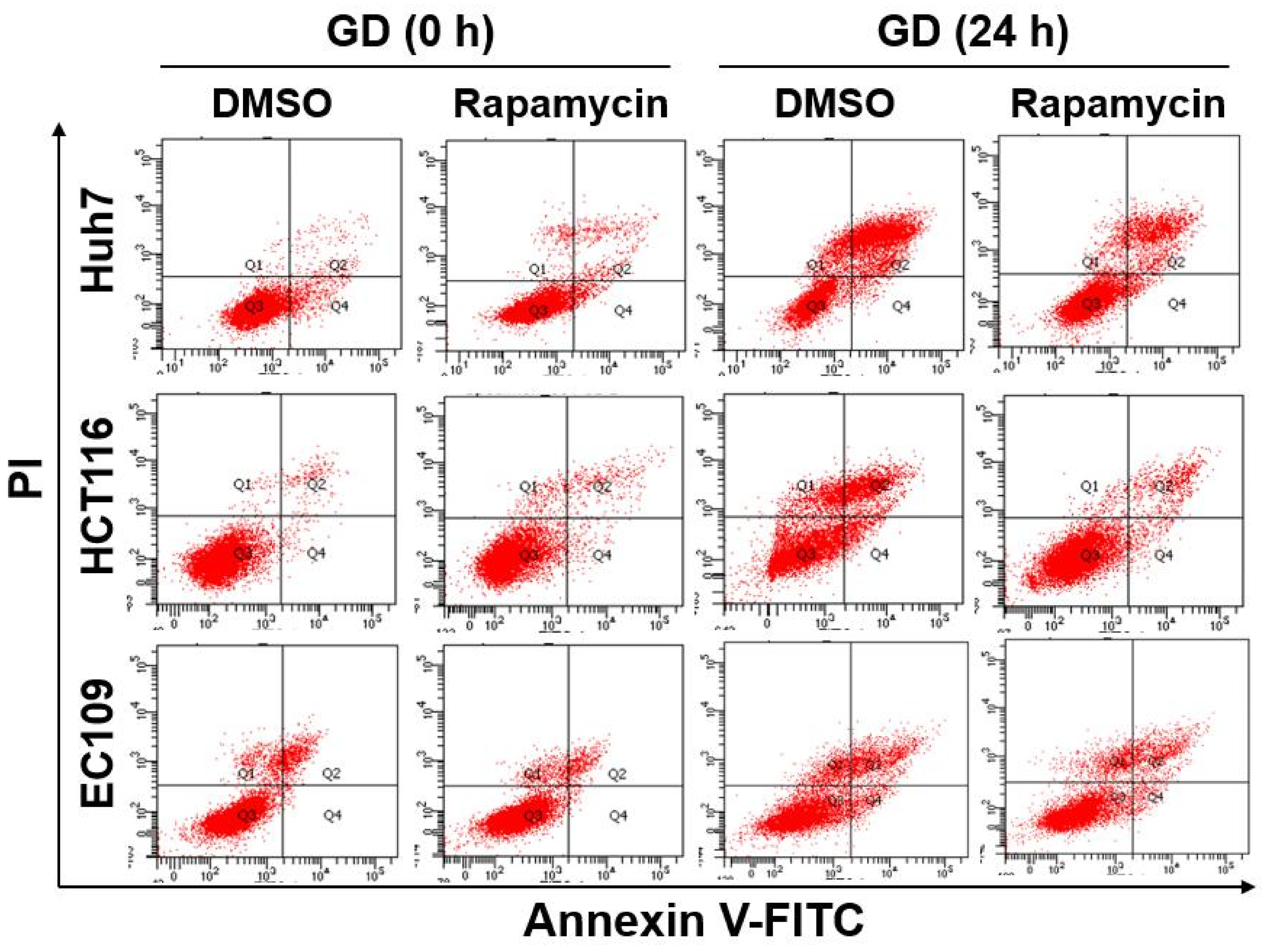

D

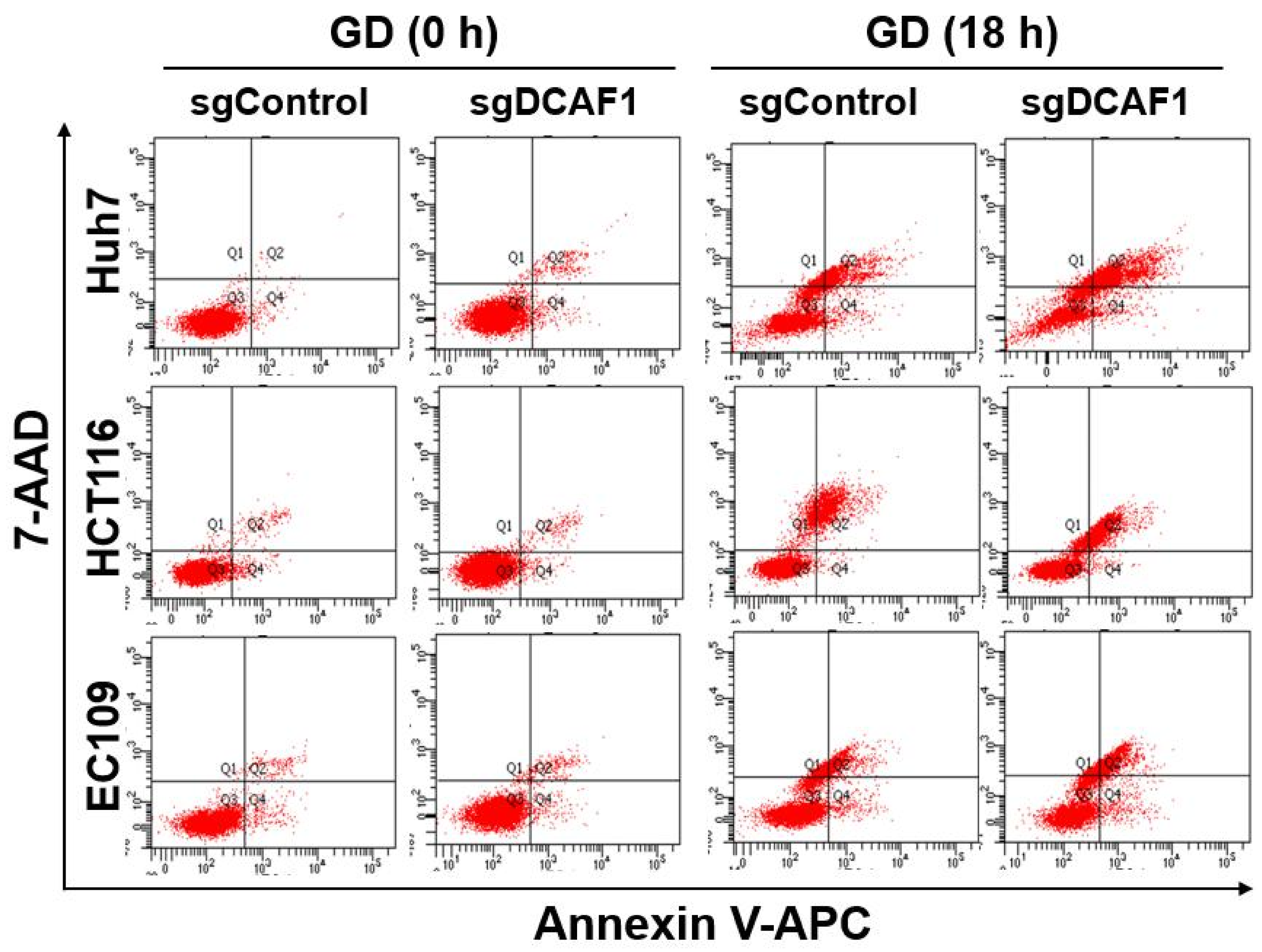

E

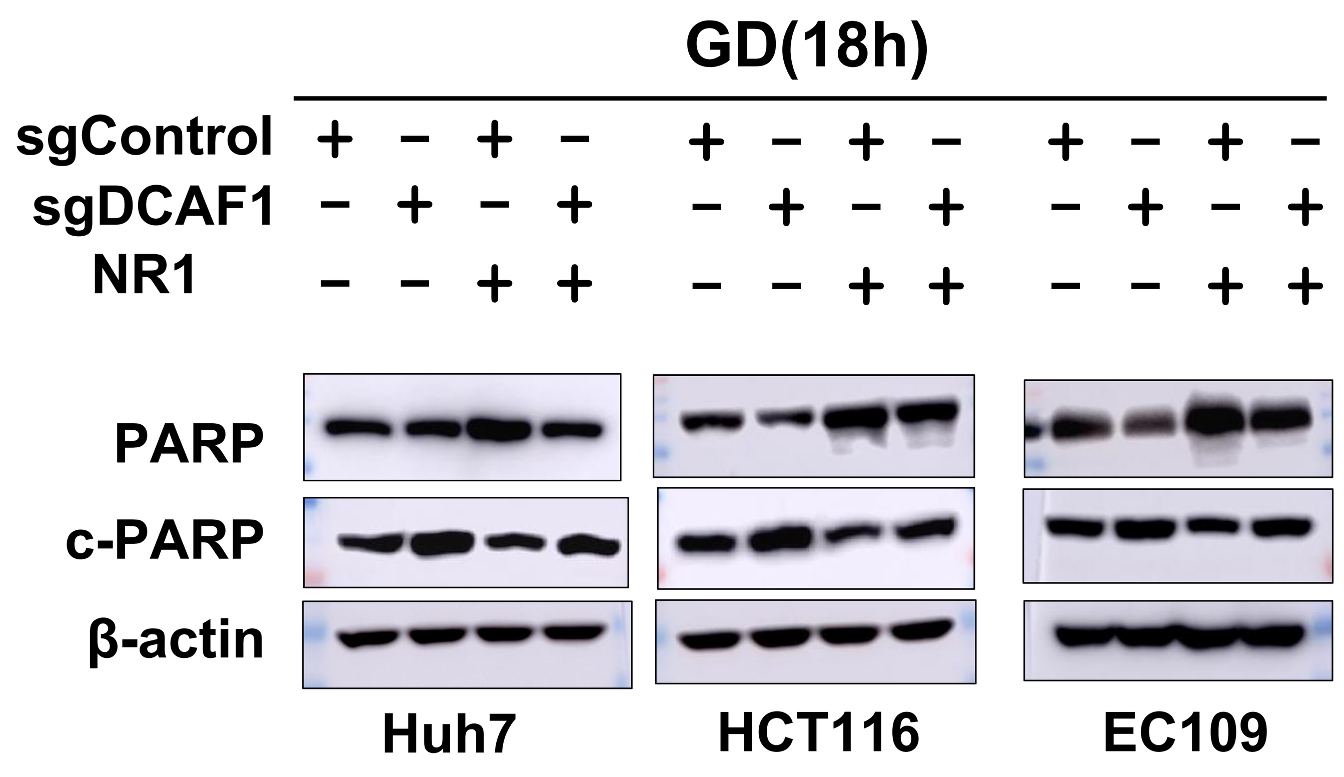

Figure S7 DCAF1 promotes cancer cell survival and protects cancer cell from glucose deprivation-induced cell death, related to Figure 7. (A) Stable Lv-Flag-DCAF1 or control Lv-EGFP cells were cultured in glucose-free medium for 24 h and collected for apoptosis using Annexin V-APC/7-AAD double staining FACS. (B-C) NR1 and rapamycin protects from cell death induced by glucose deprivation. Huh7, HCT116, and EC109 cells were cultured in glucose-free medium and treated with NR1 (B) or rapamycin (C). 24 h later, cells were collected for apoptosis analysis using Annexin V-FITC/PI double staining FACS. (D) Silencing DCAF1 promotes glucose deprivation induced cell death. Stable sgDCAF1 or control sgControl cells were cultured in glucose-free medium for 18 h and collected for apoptosis analysis using Annexin V-APC/7-AAD double staining FACS. (E) NR1 rescues cell death promoted by sgDCAF1 under glucose deprivation. Stable sgDCAF1 or sgControl cells were cultured in glucose-free medium and treated with NR1 for 18 h. Cells were collected for IB analysis. All data were representative of at least three independent experiments (n=3).
