# Supplementary material for: Glucose deprivation triggers DCAF1-mediated inactivation of Rheb-mTORC1 and promotes cancer cell survival
Source: Cell Death Dis. 2024 Jun 11;15(6):409. doi: 10.1038/s41419-024-06808-1 (PMC11166663; doi:10.1038/s41419-024-06808-1)

Figure 1A

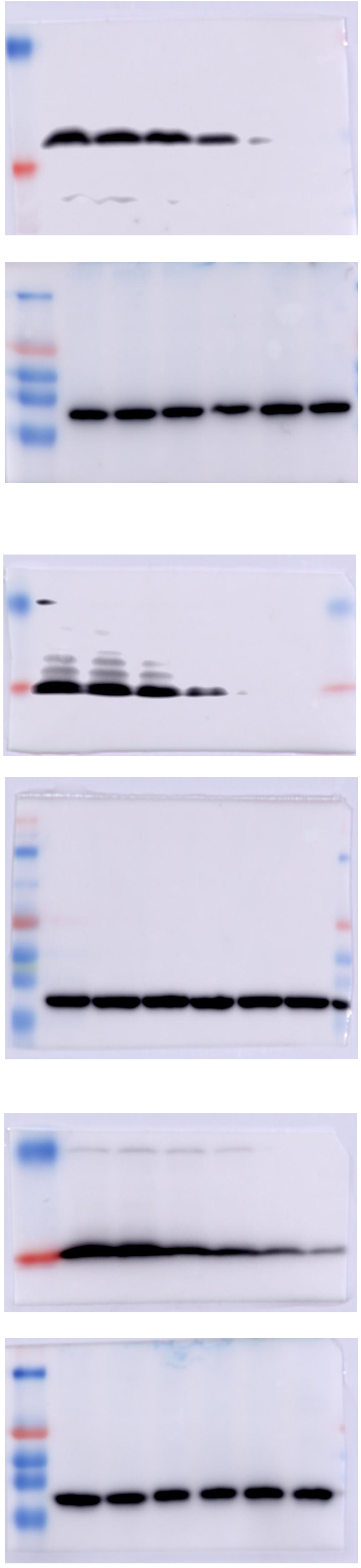

Figure 1B

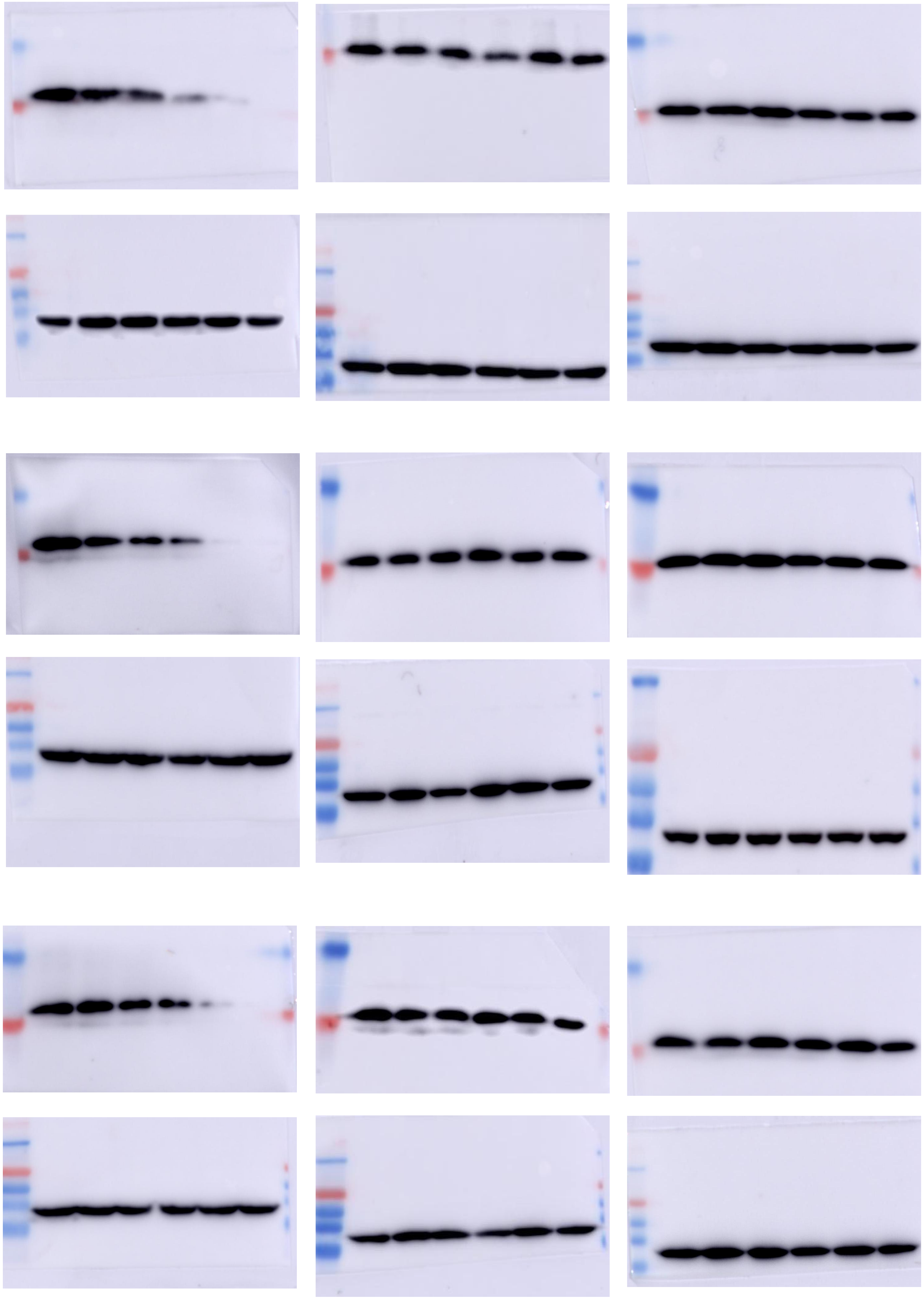

Figure 1C

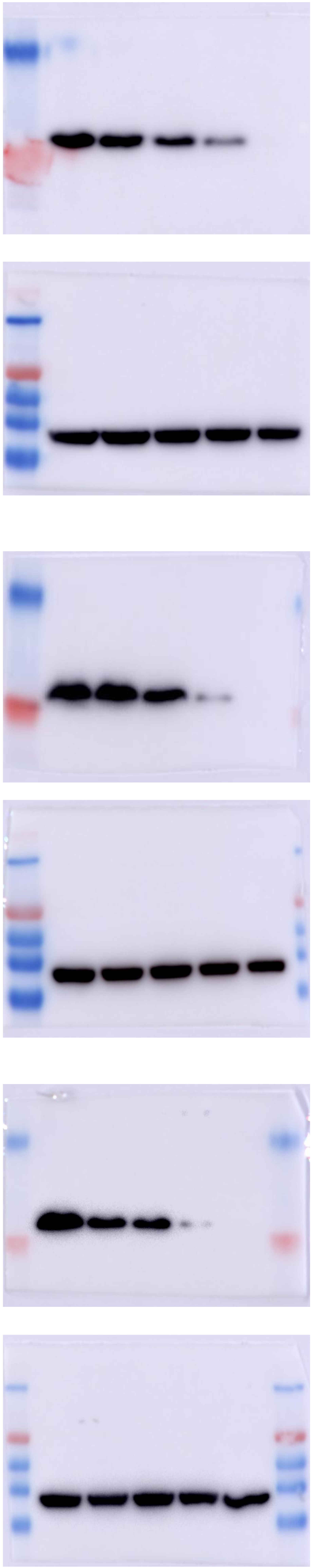

Figure 1D

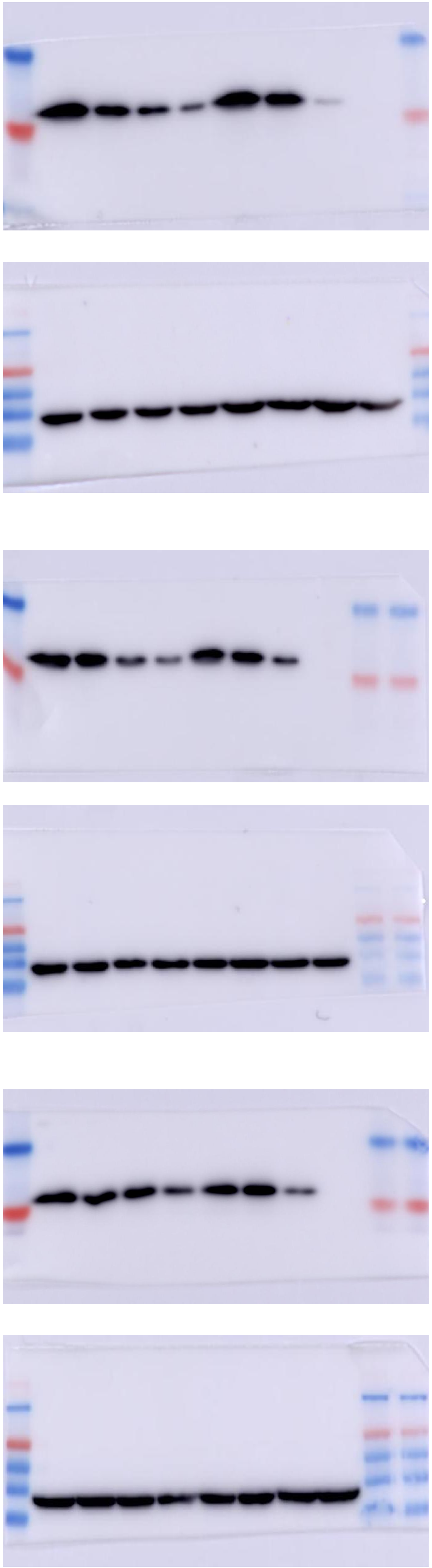

Figure 1E

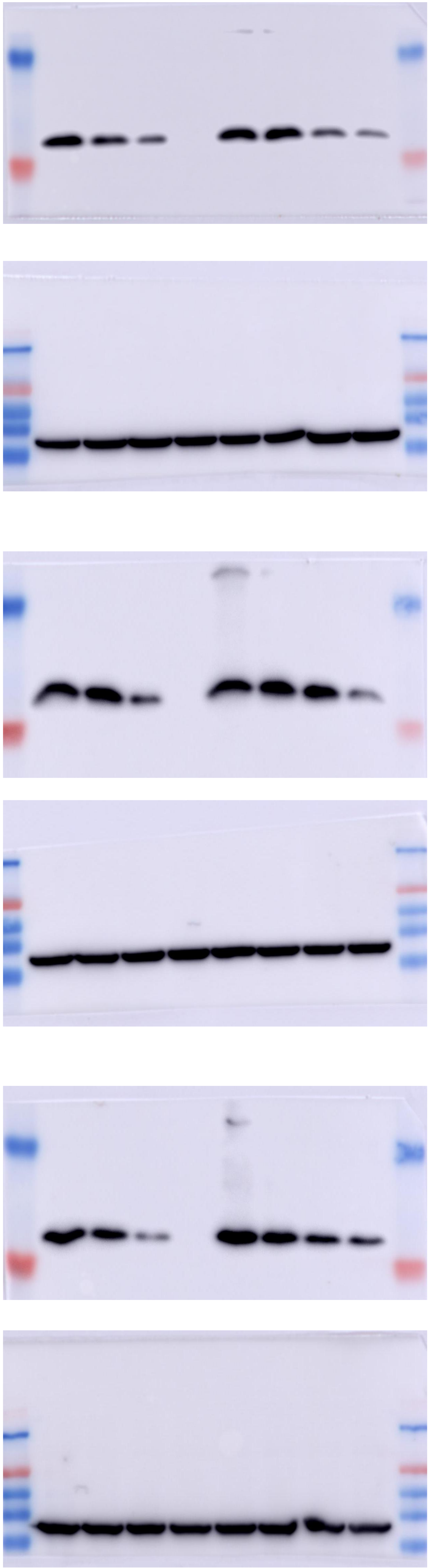

Figure 1F

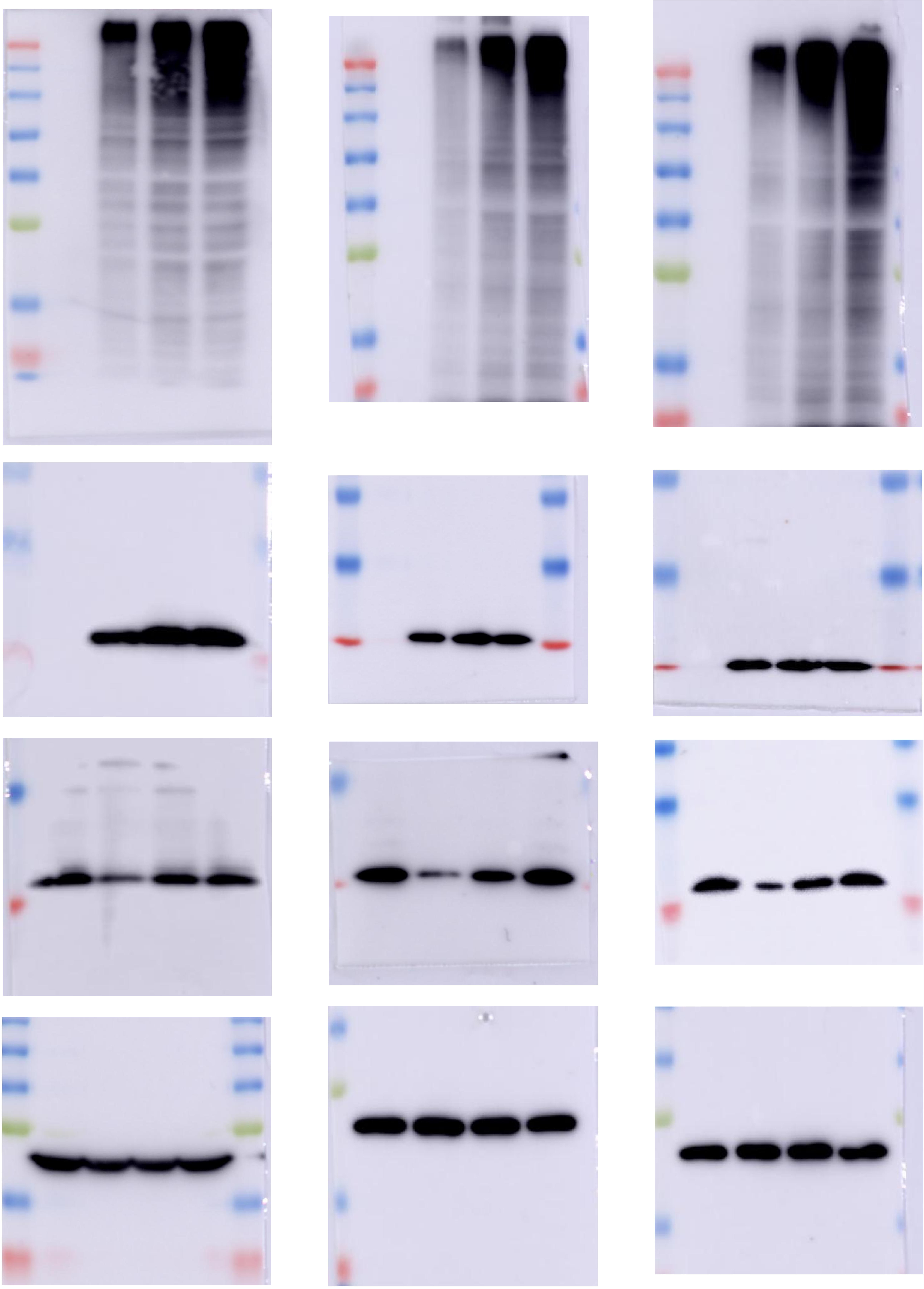

Figure 1G

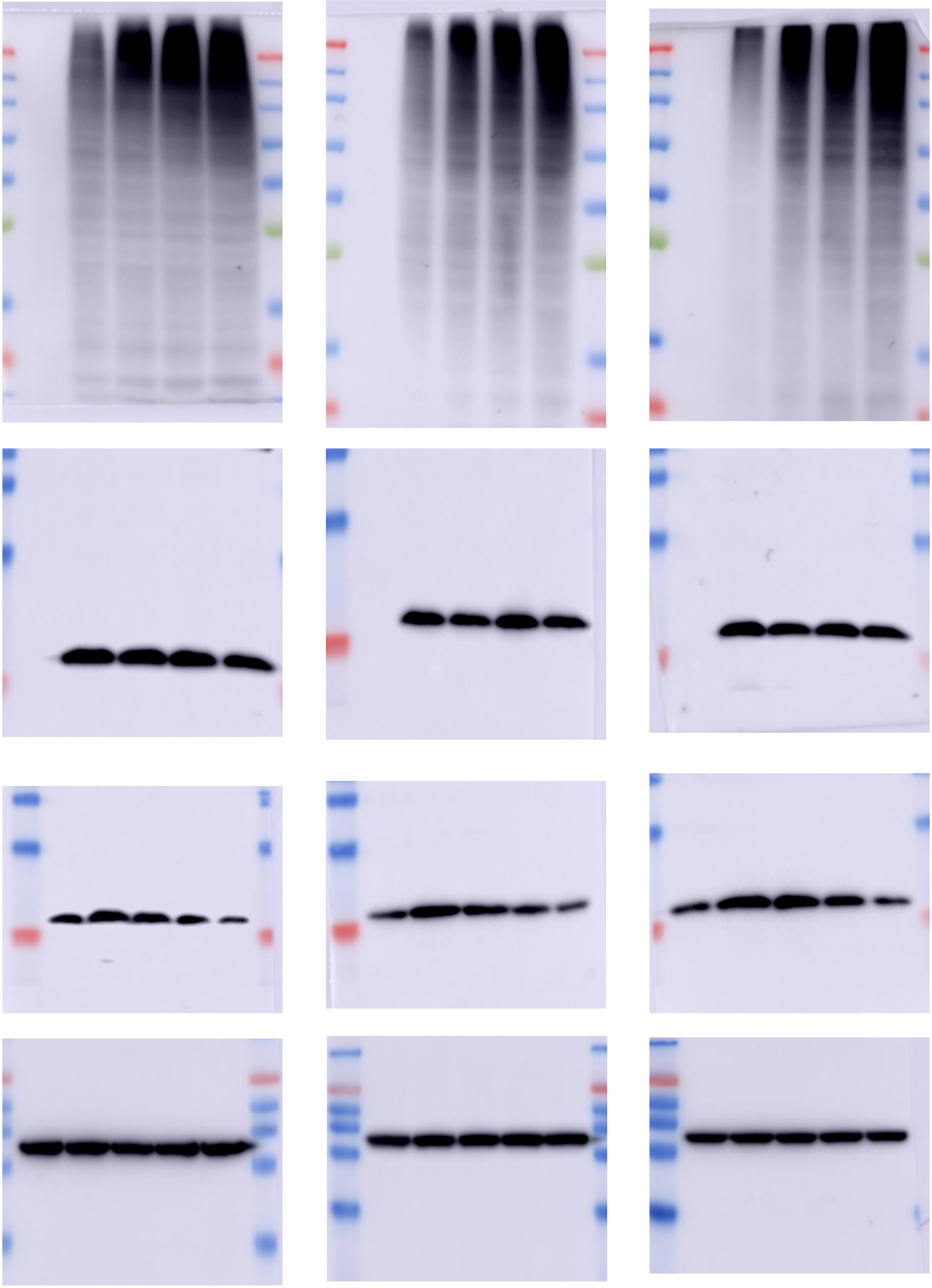

Figure 1H

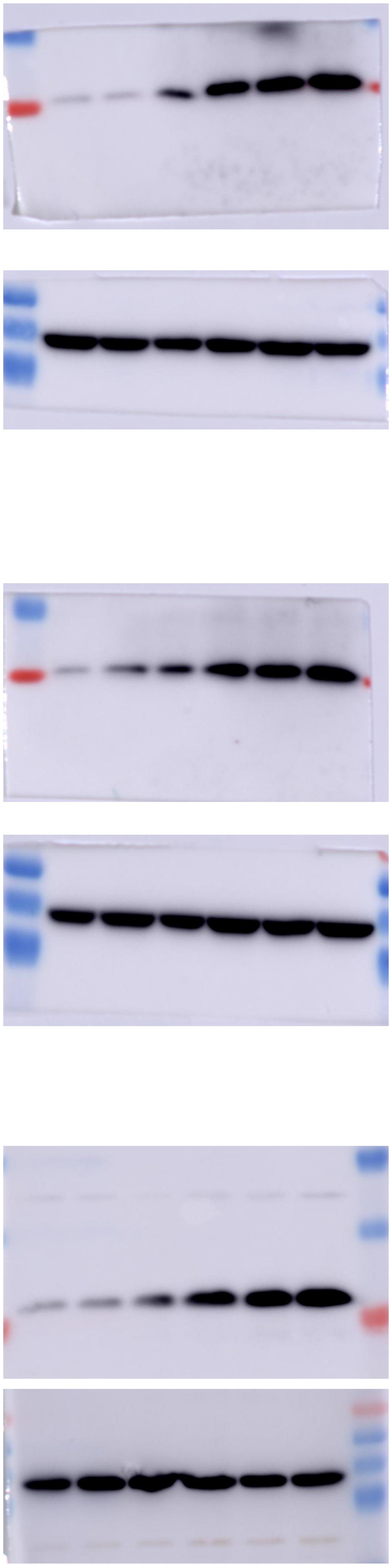

Figure 1I

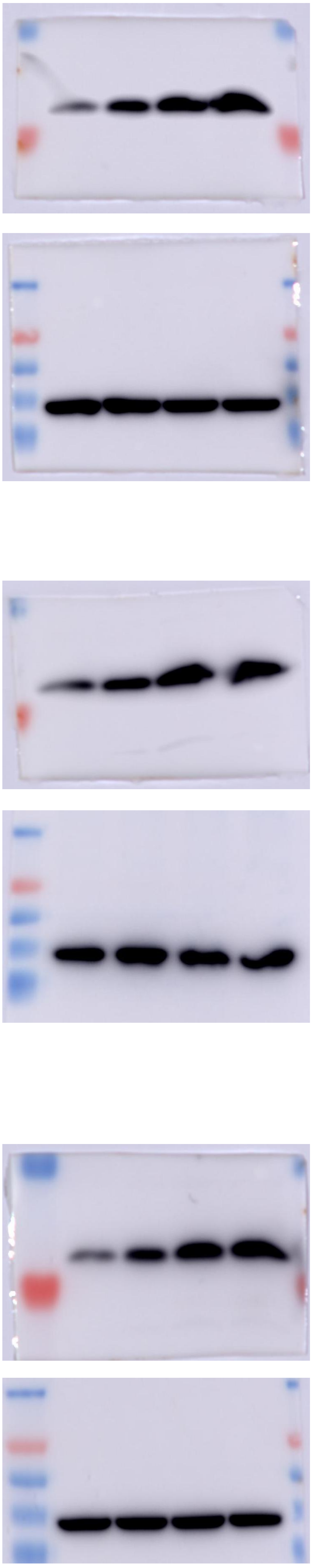

Figure 1J

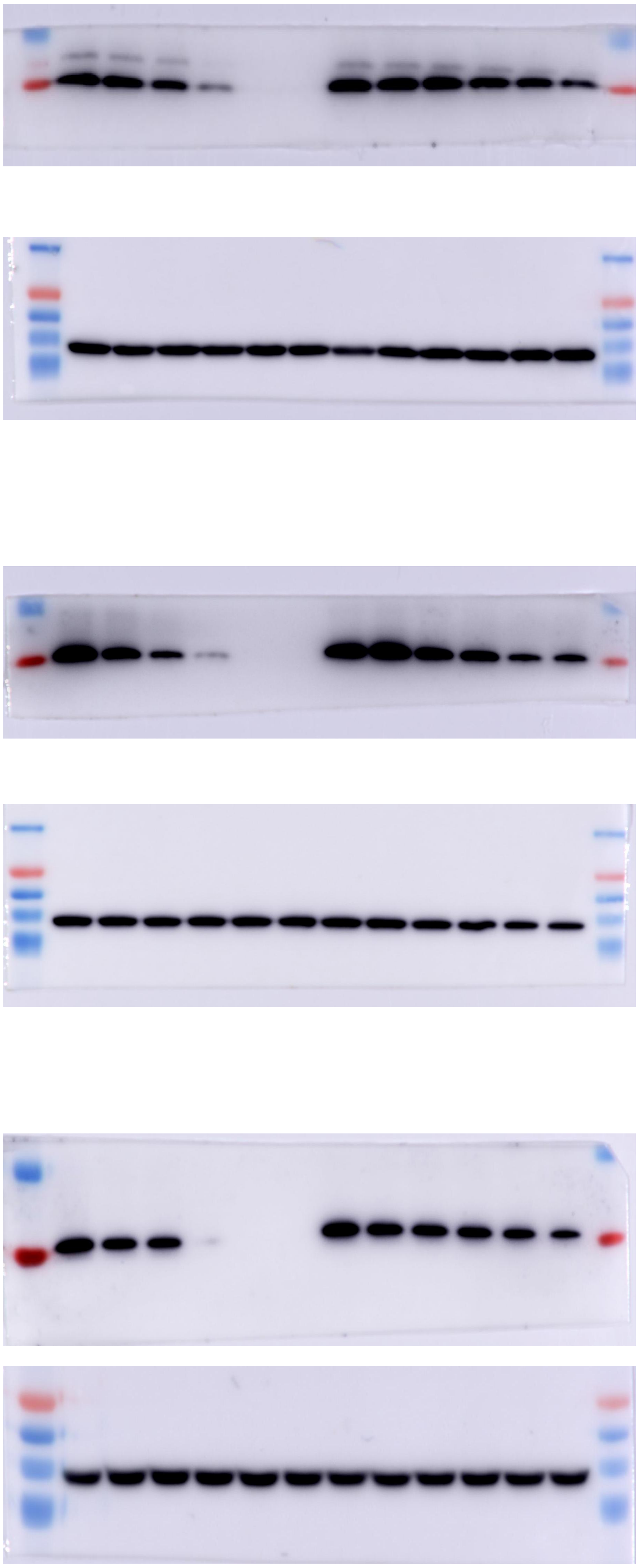

Figure 2B

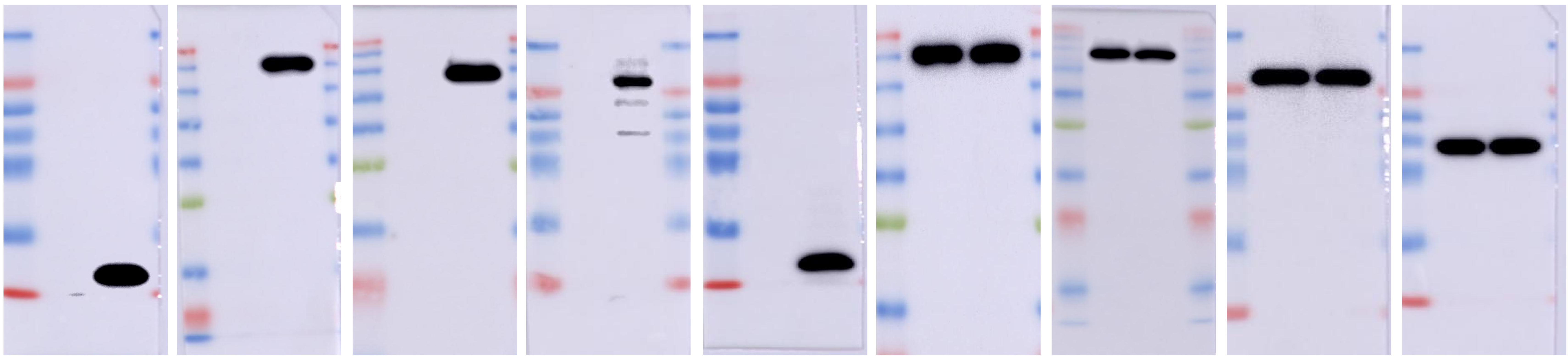

Figure 2C

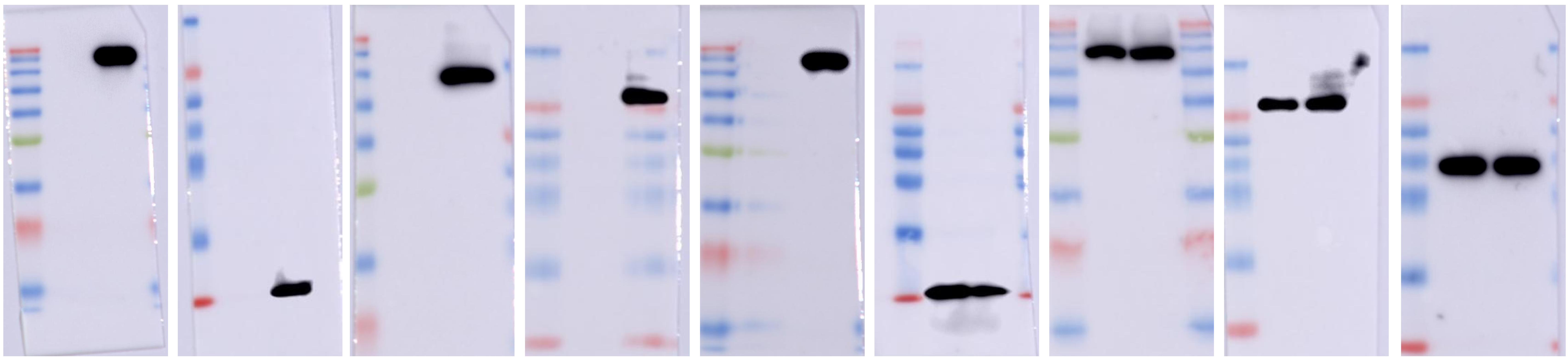

Figure 2D

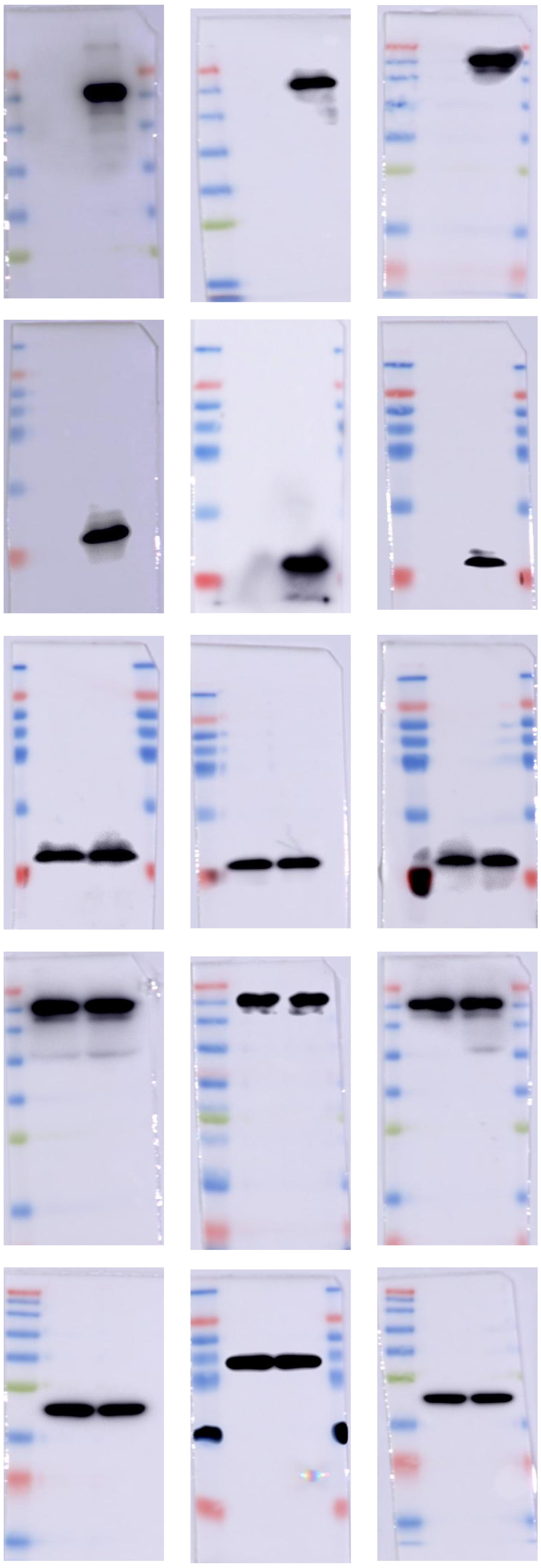

Figure 2E

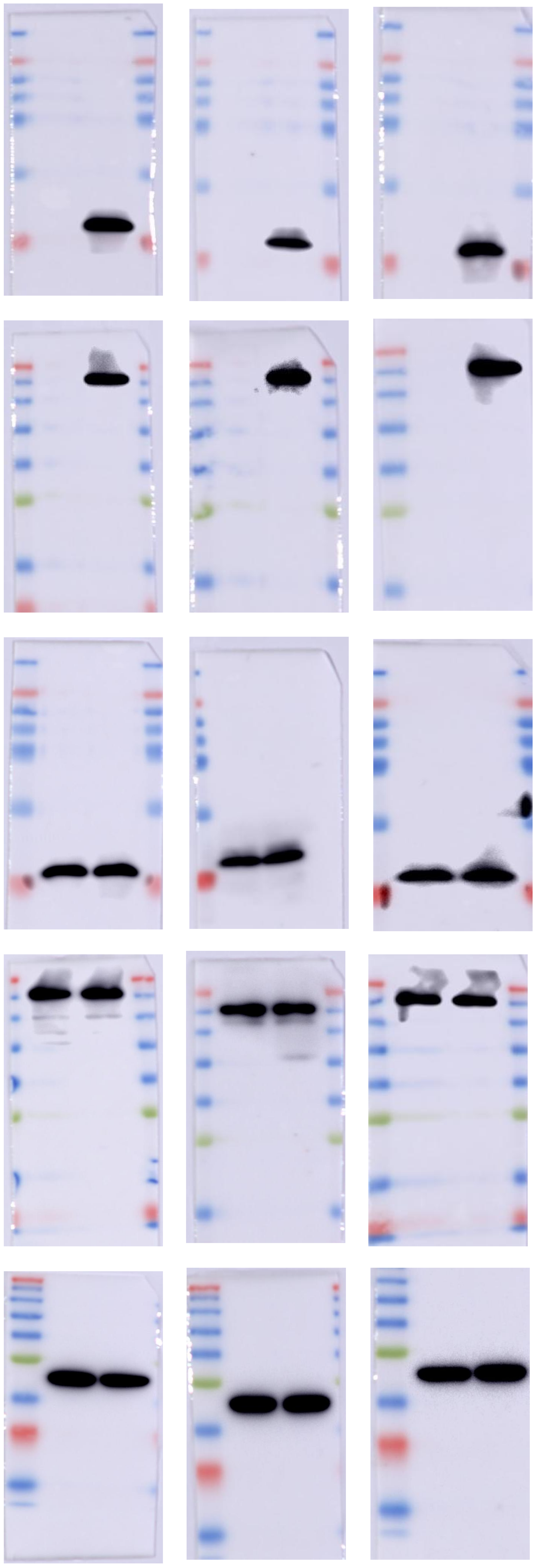

Figure 2G

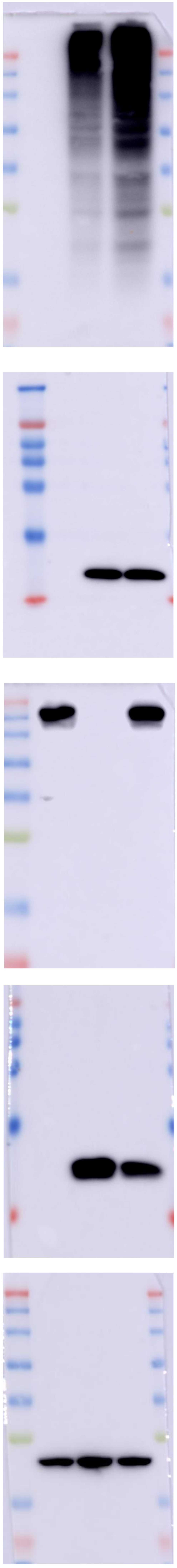

Figure 2H

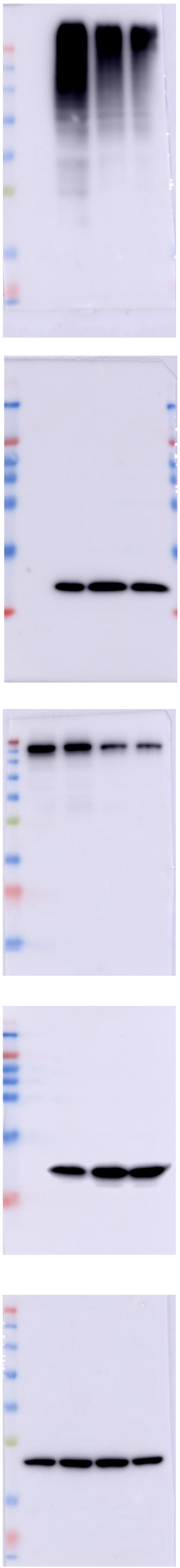

Figure 2I

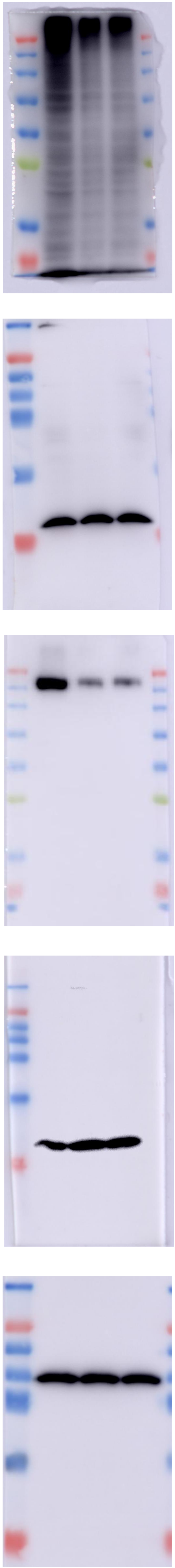

Figure 2J

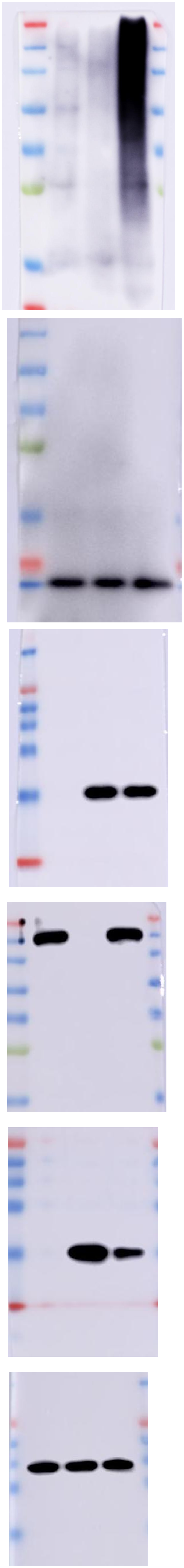

Figure 3A

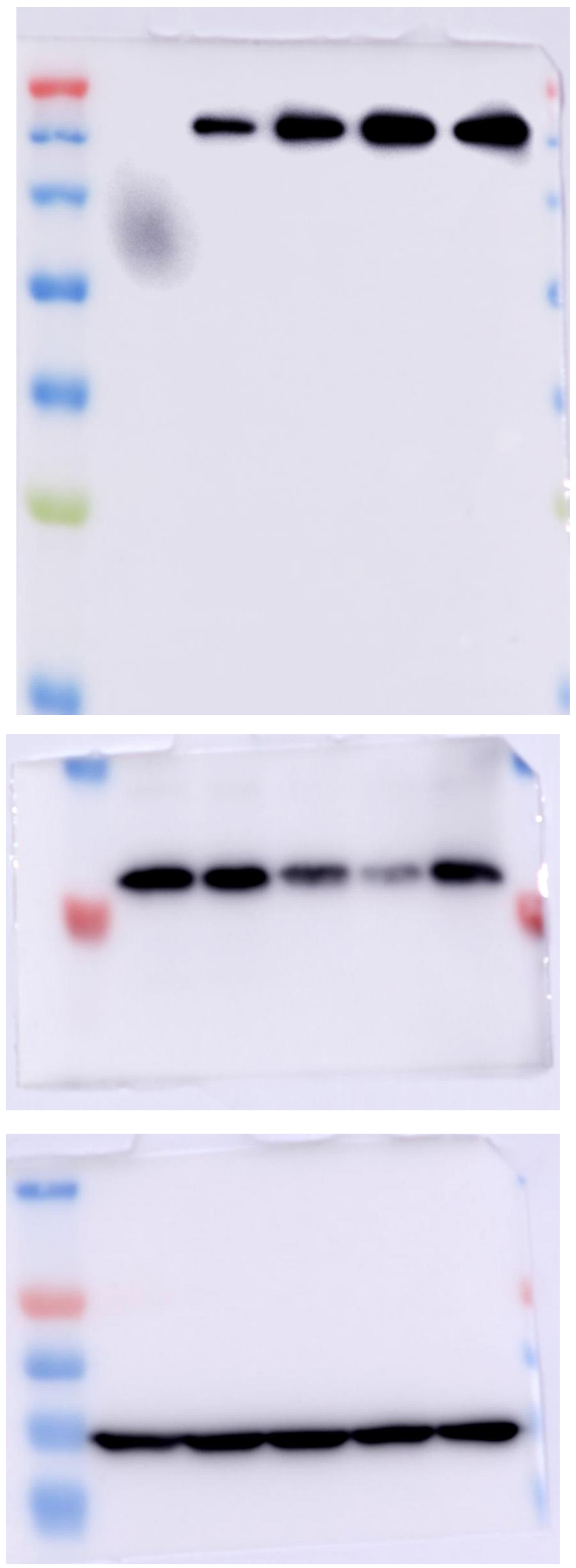

Figure 3B

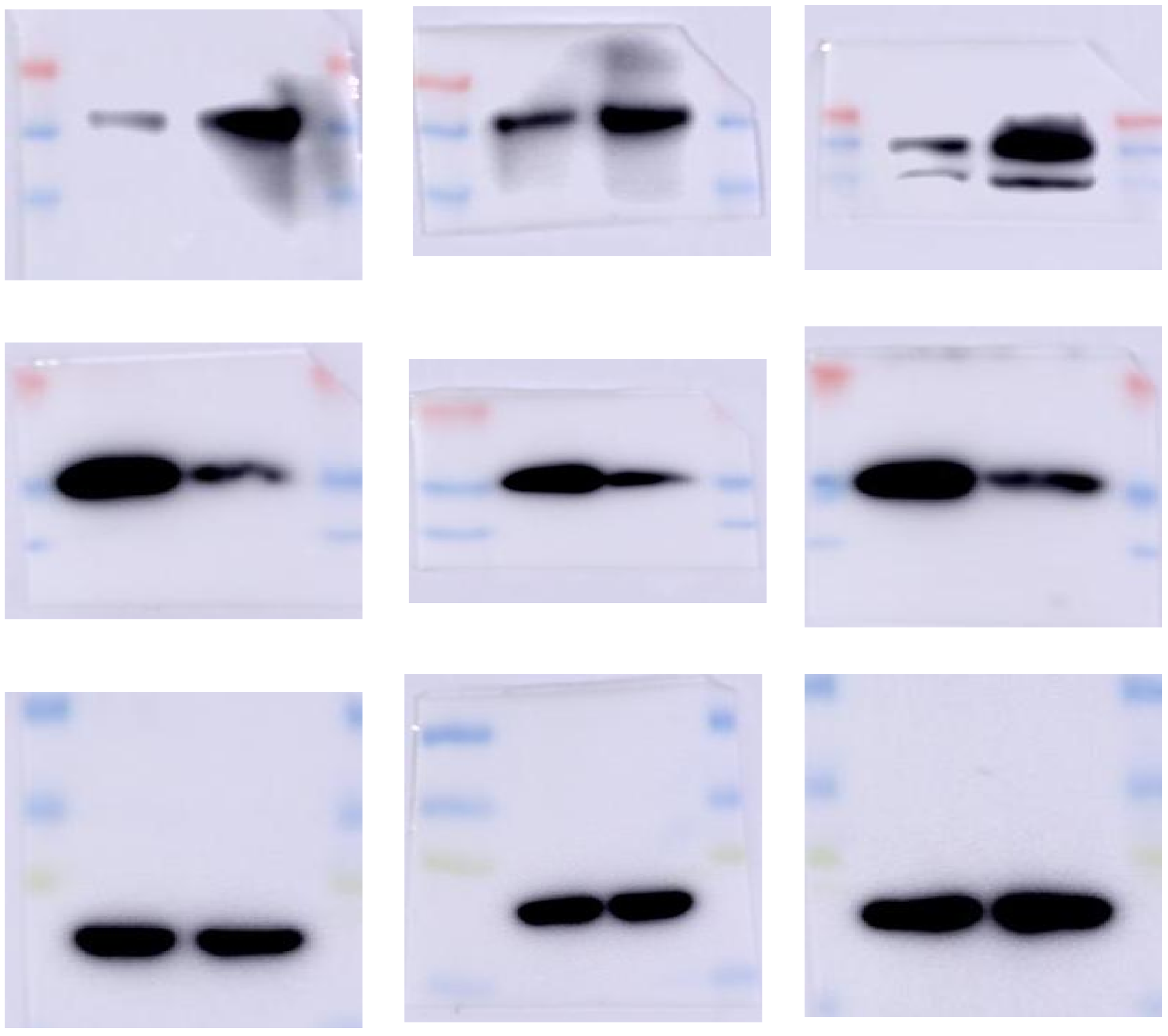

Figure 3C

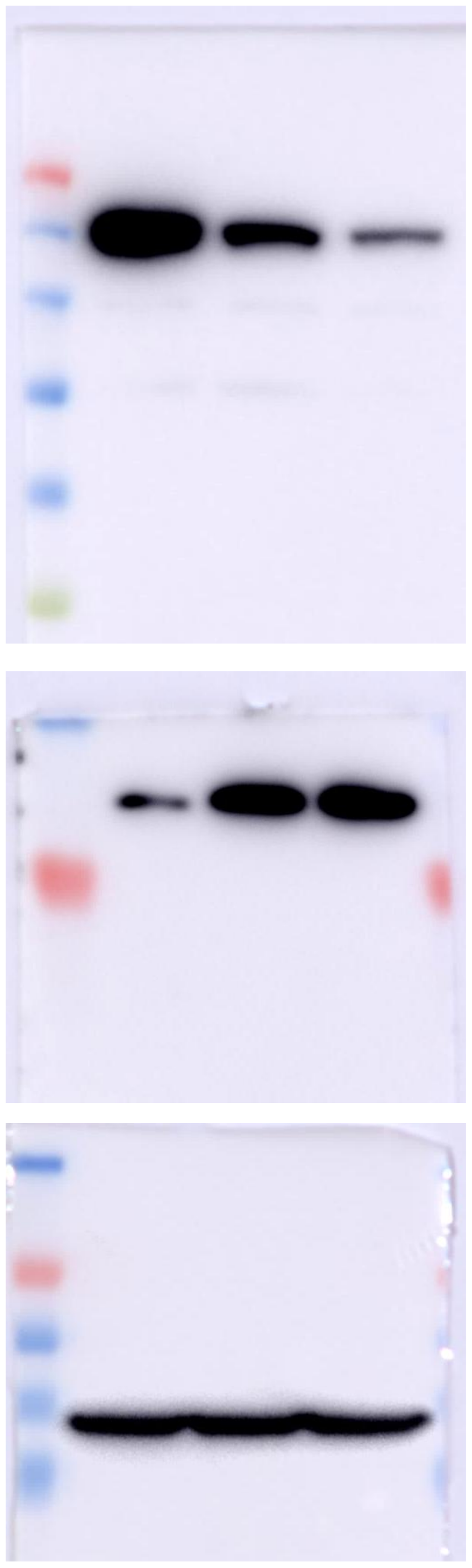

Figure 3D

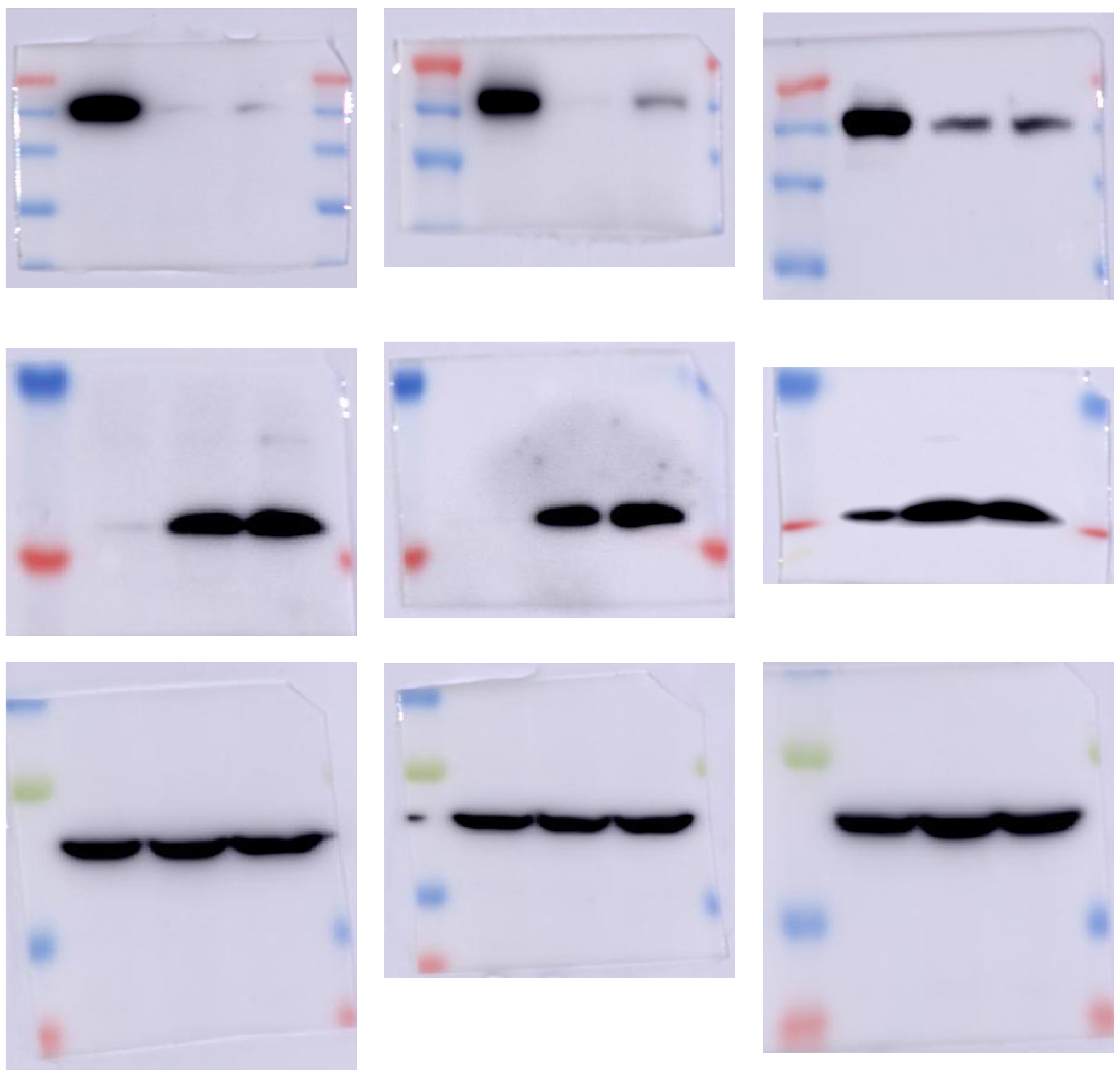

Figure 3E

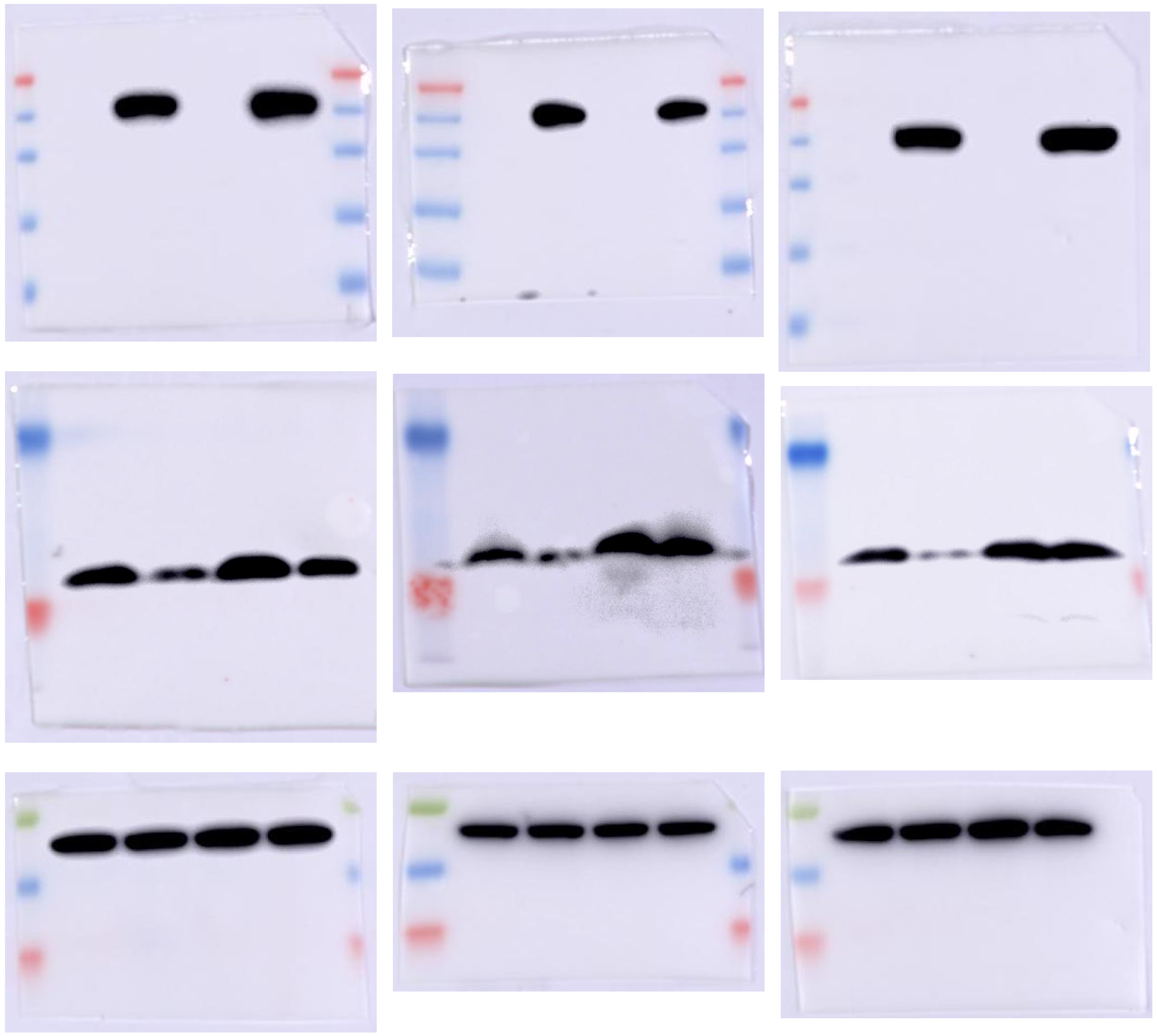

Figure 3F

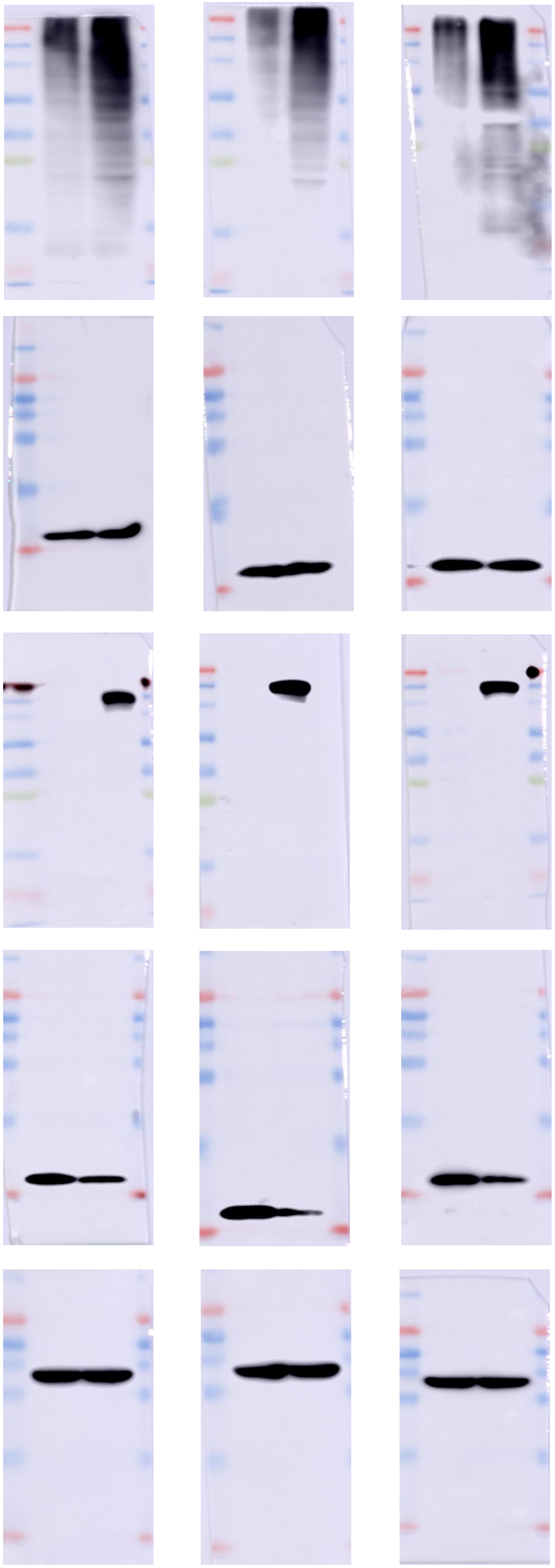

Figure 3G

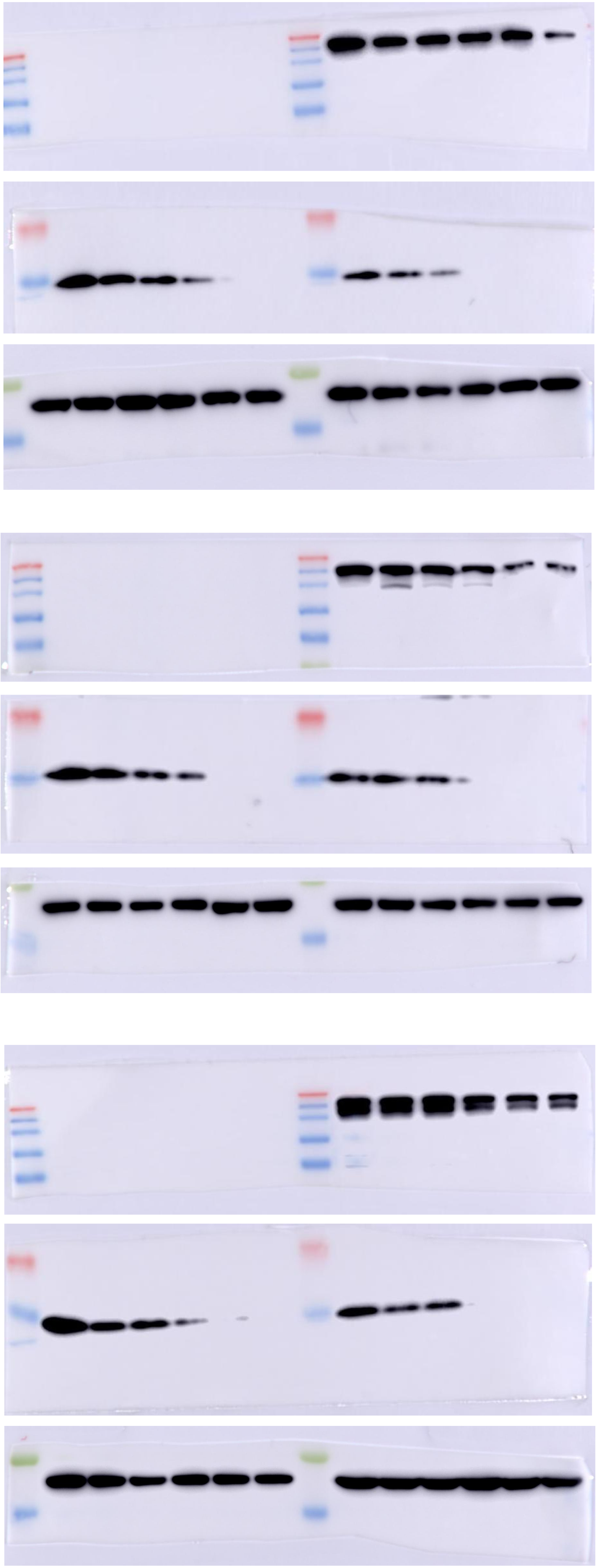

Figure 3H

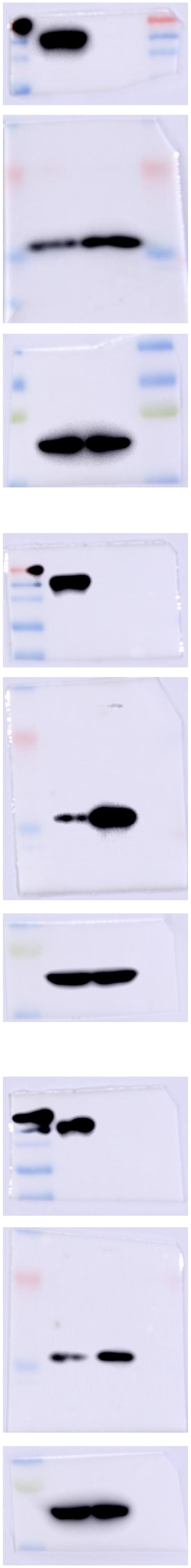

Figure 3I

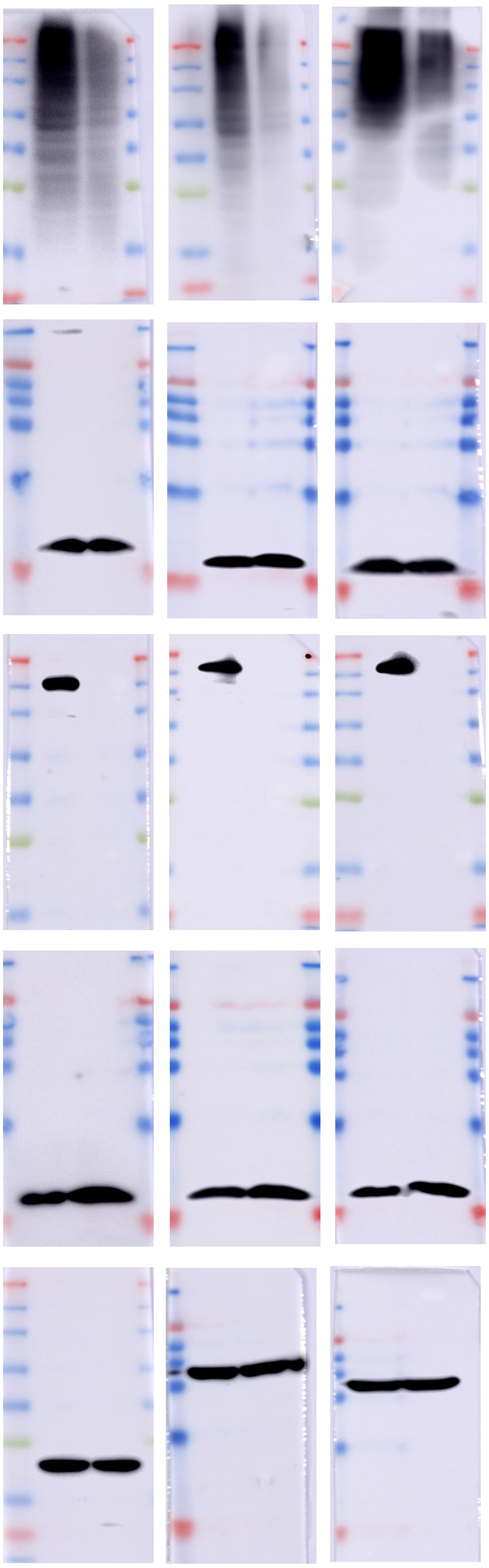

Figure 3J

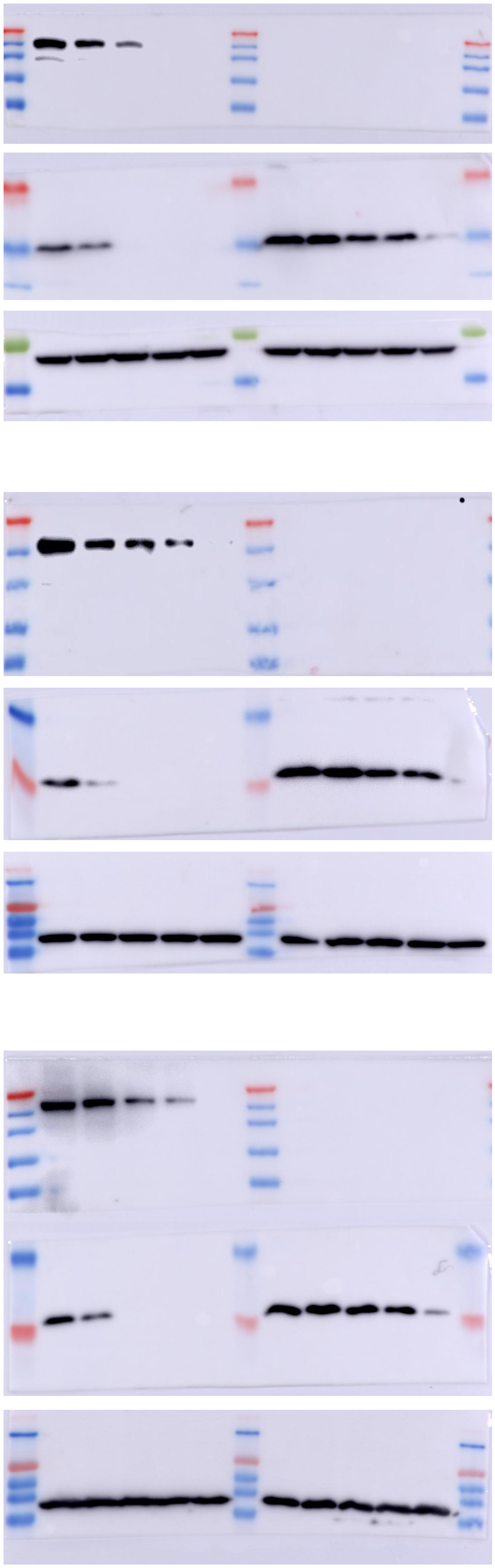

Figure 4A

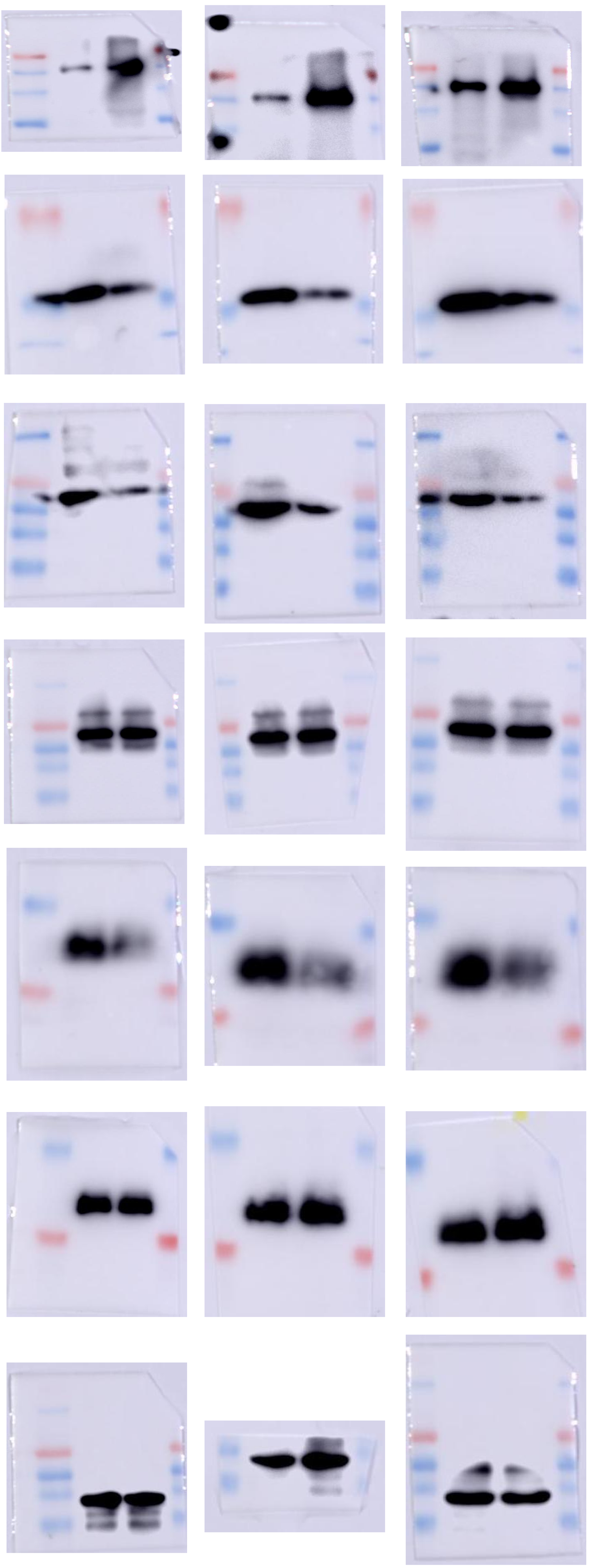

Figure 4B

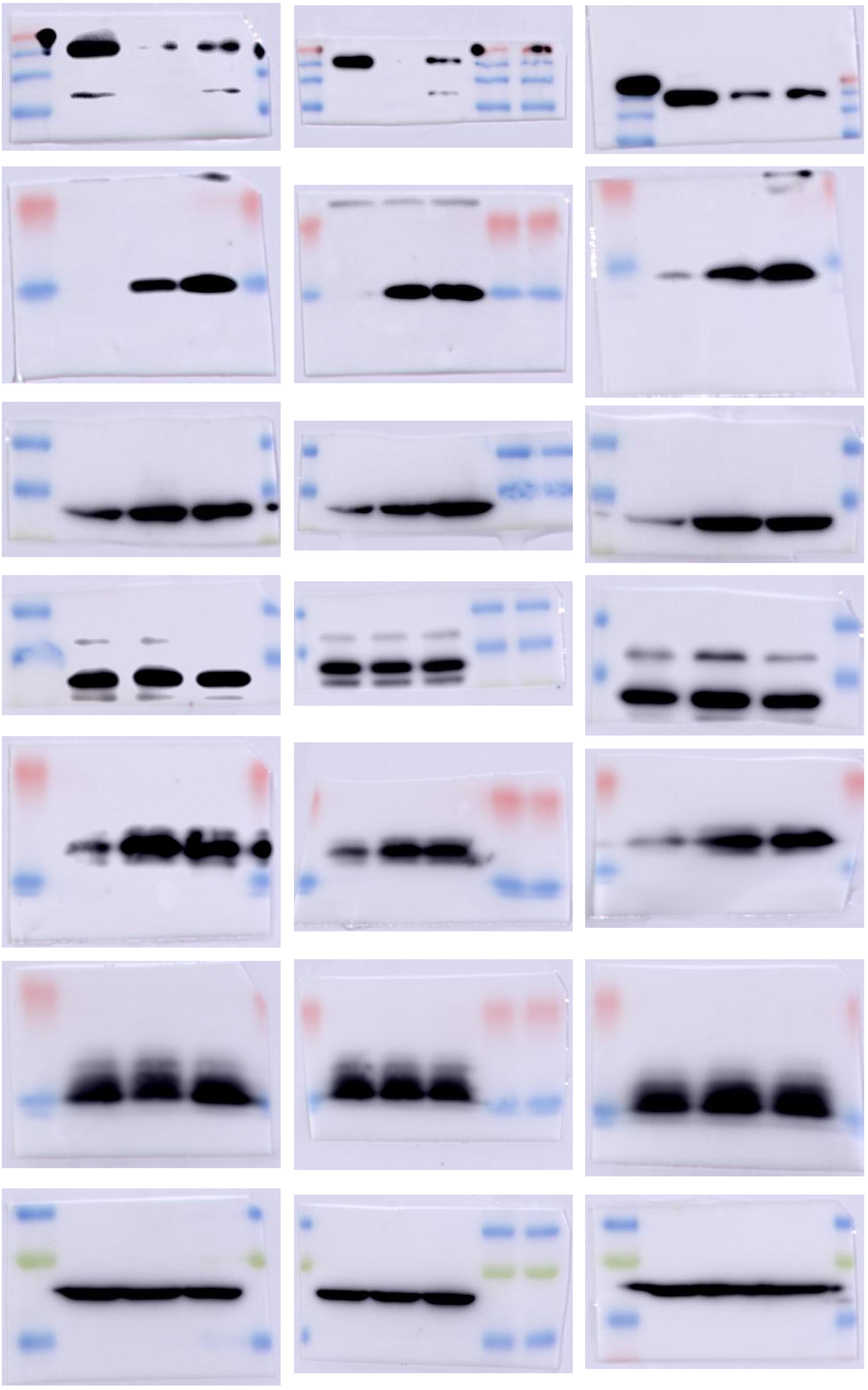

Figure 4C

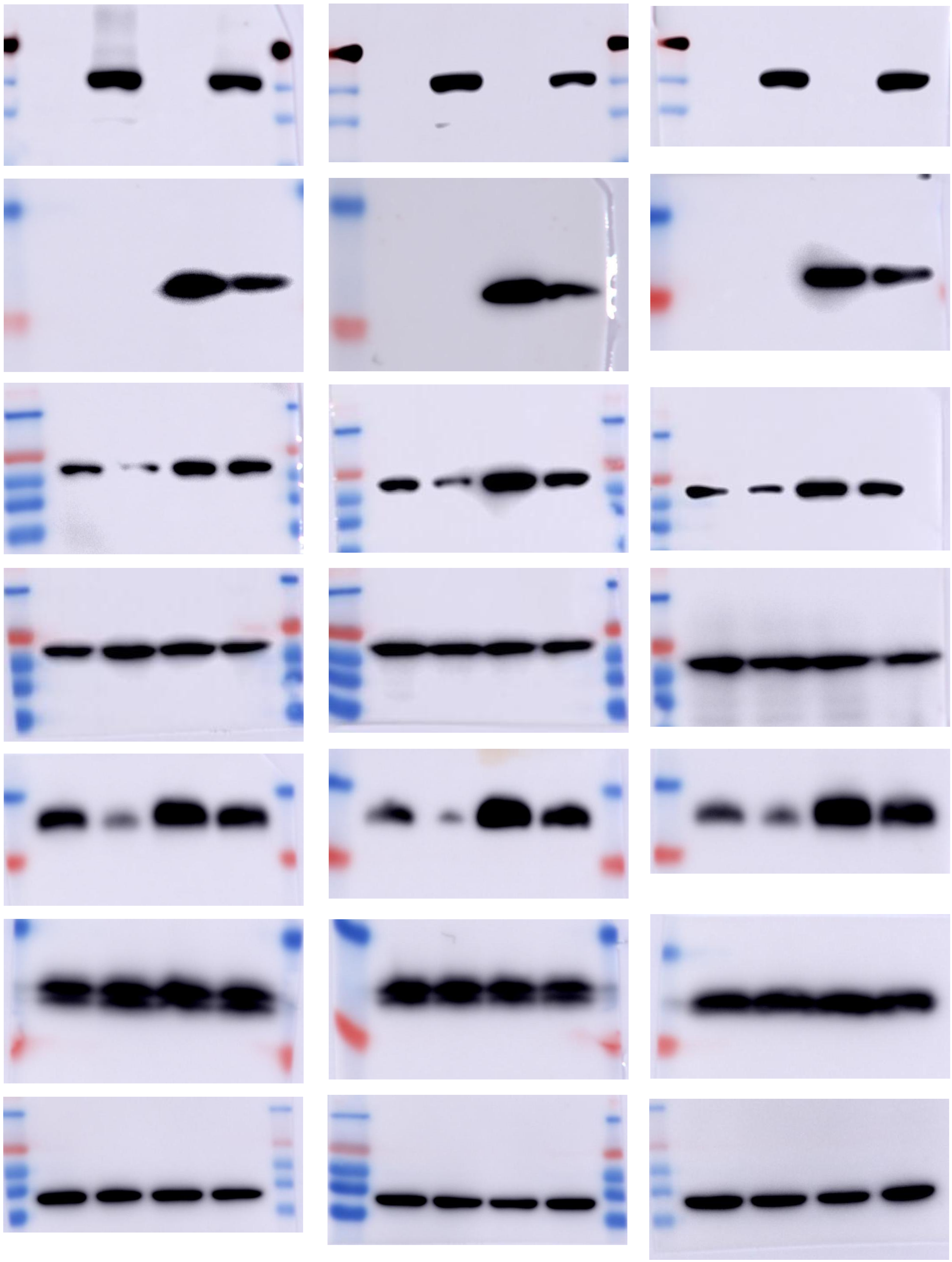

Figure 4D

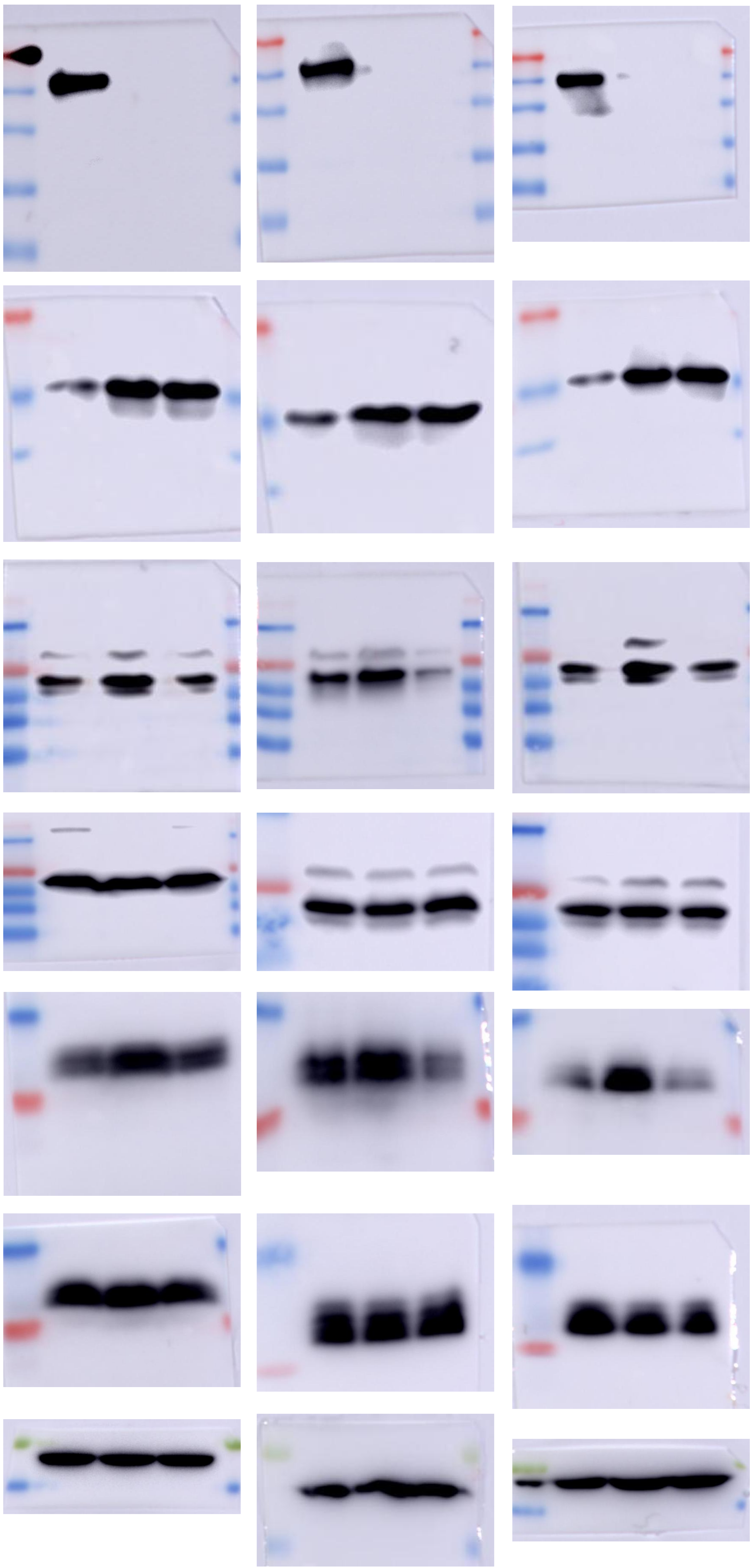

Figure 4F

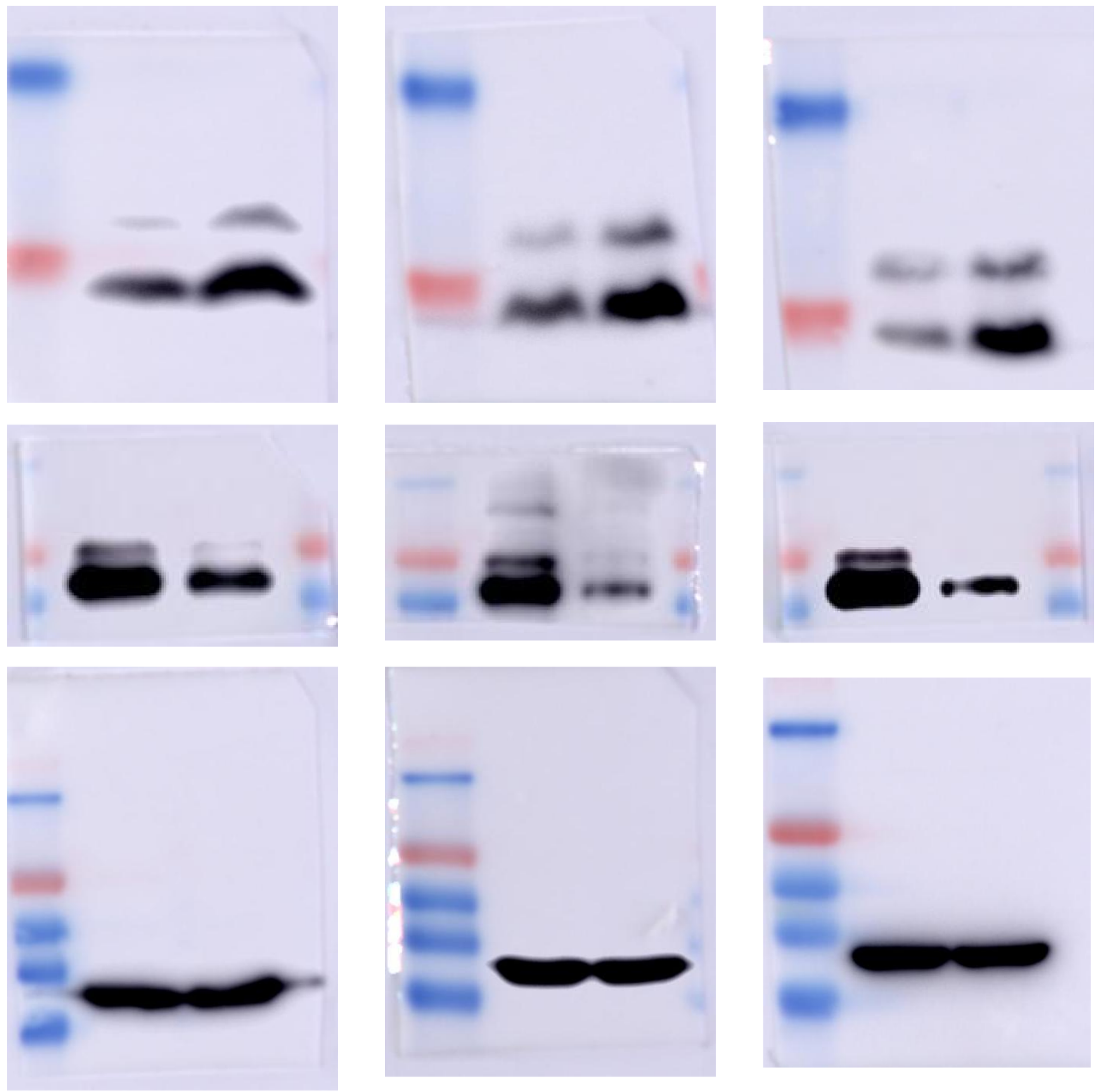

Figure 4G

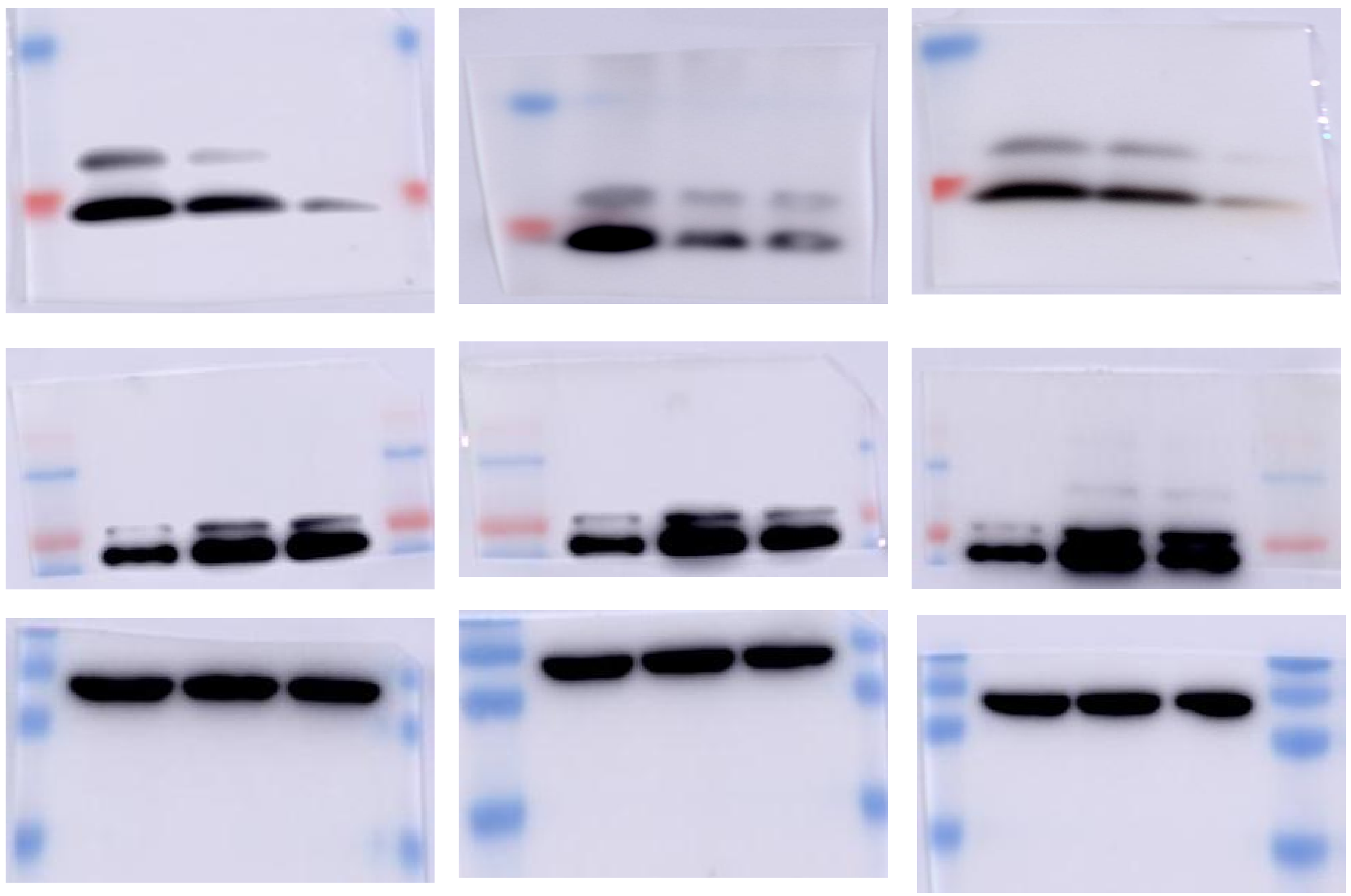

Figure 4H

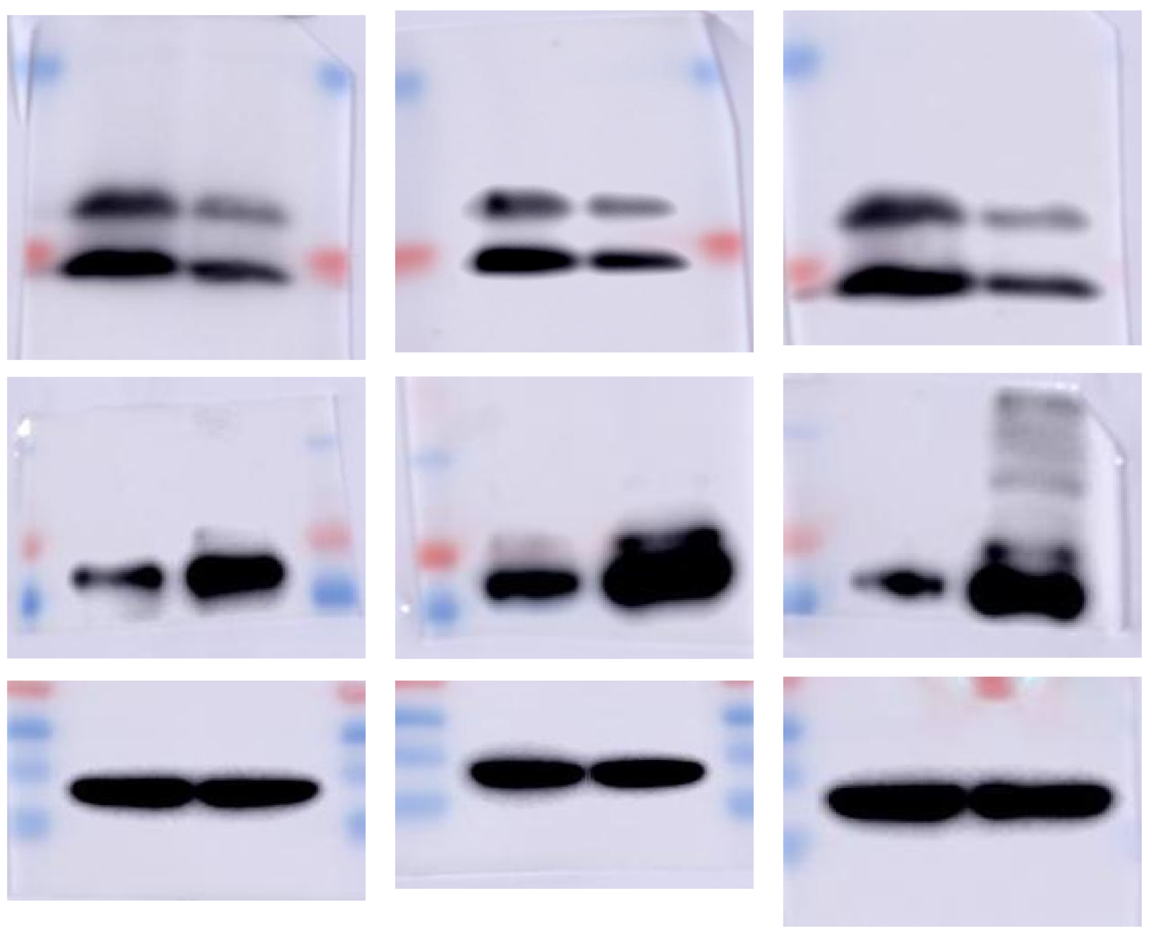

Figure 5A

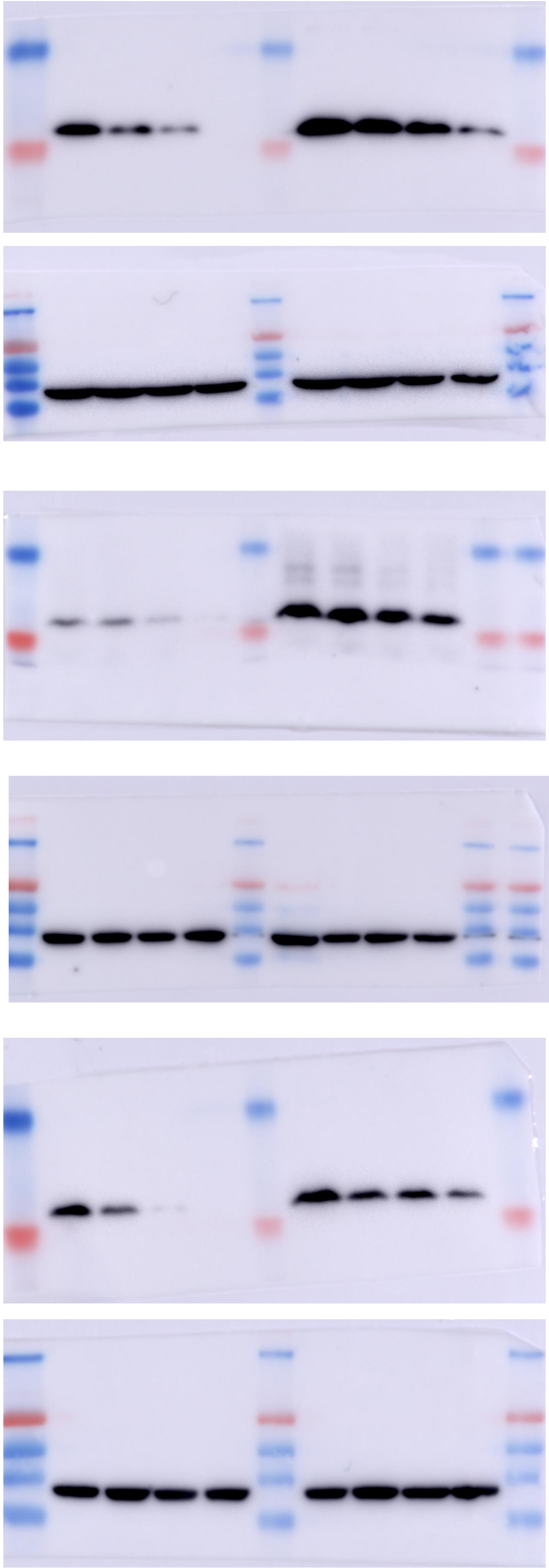

Figure 5B

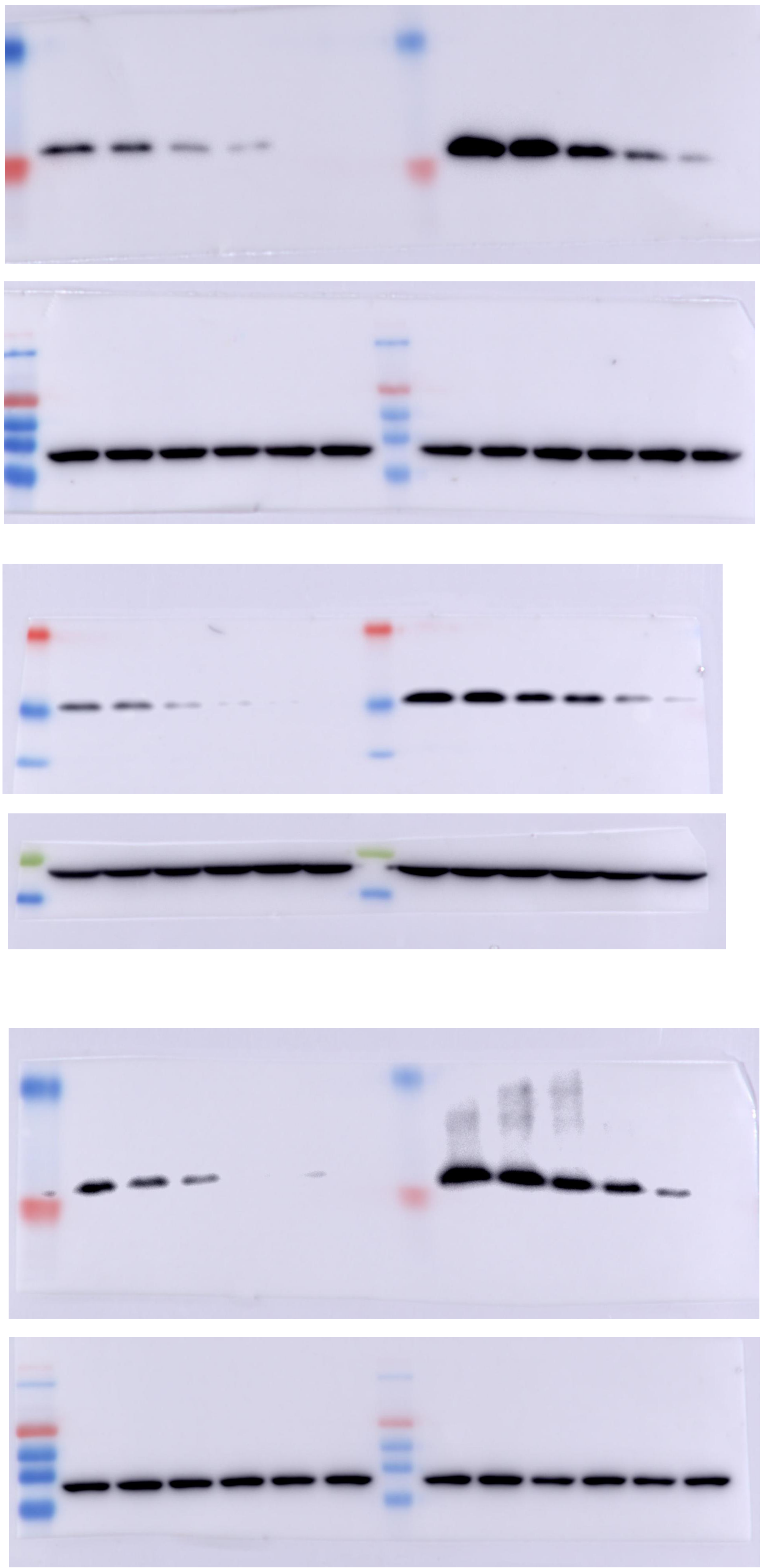

Figure 5C

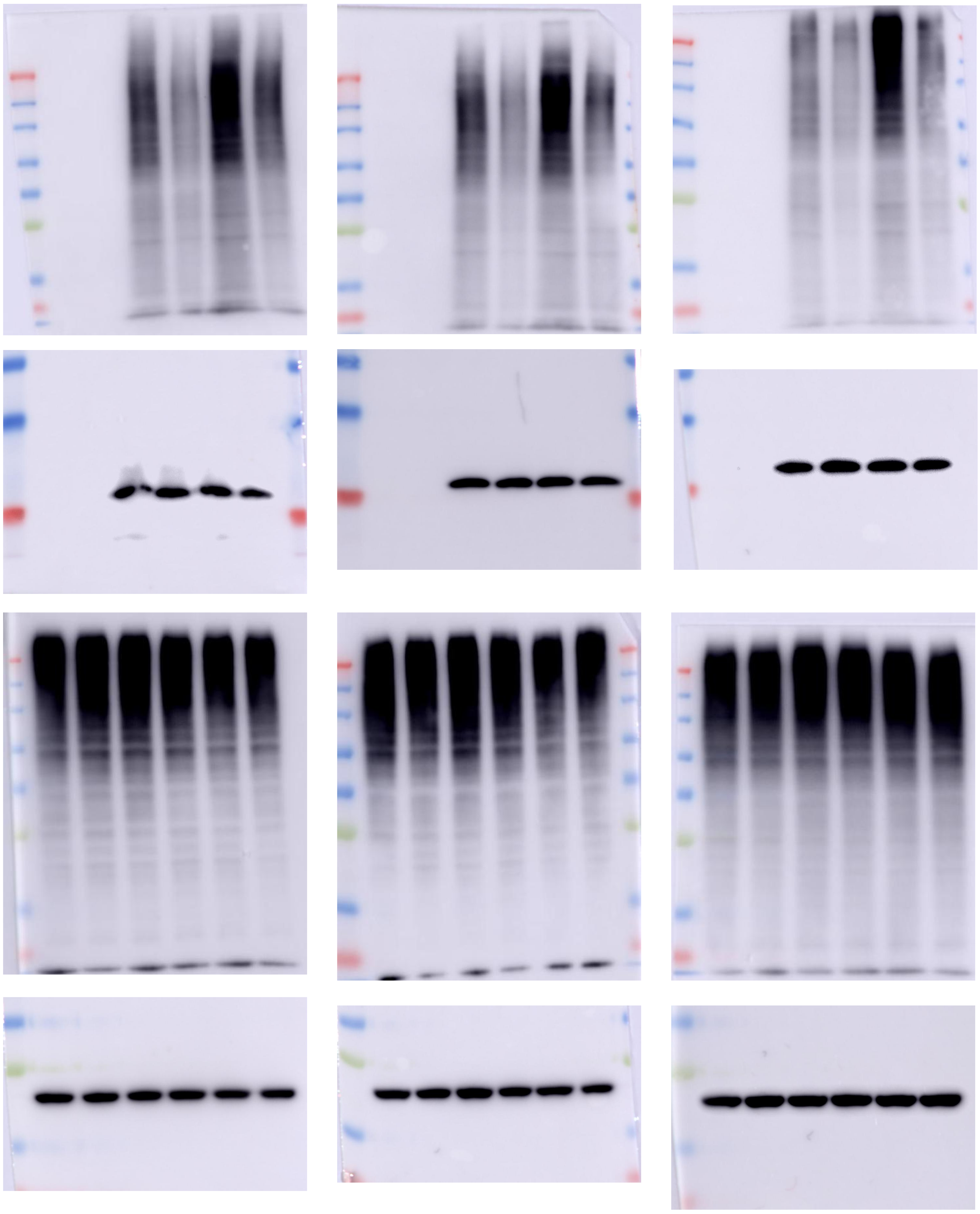

Figure 5D

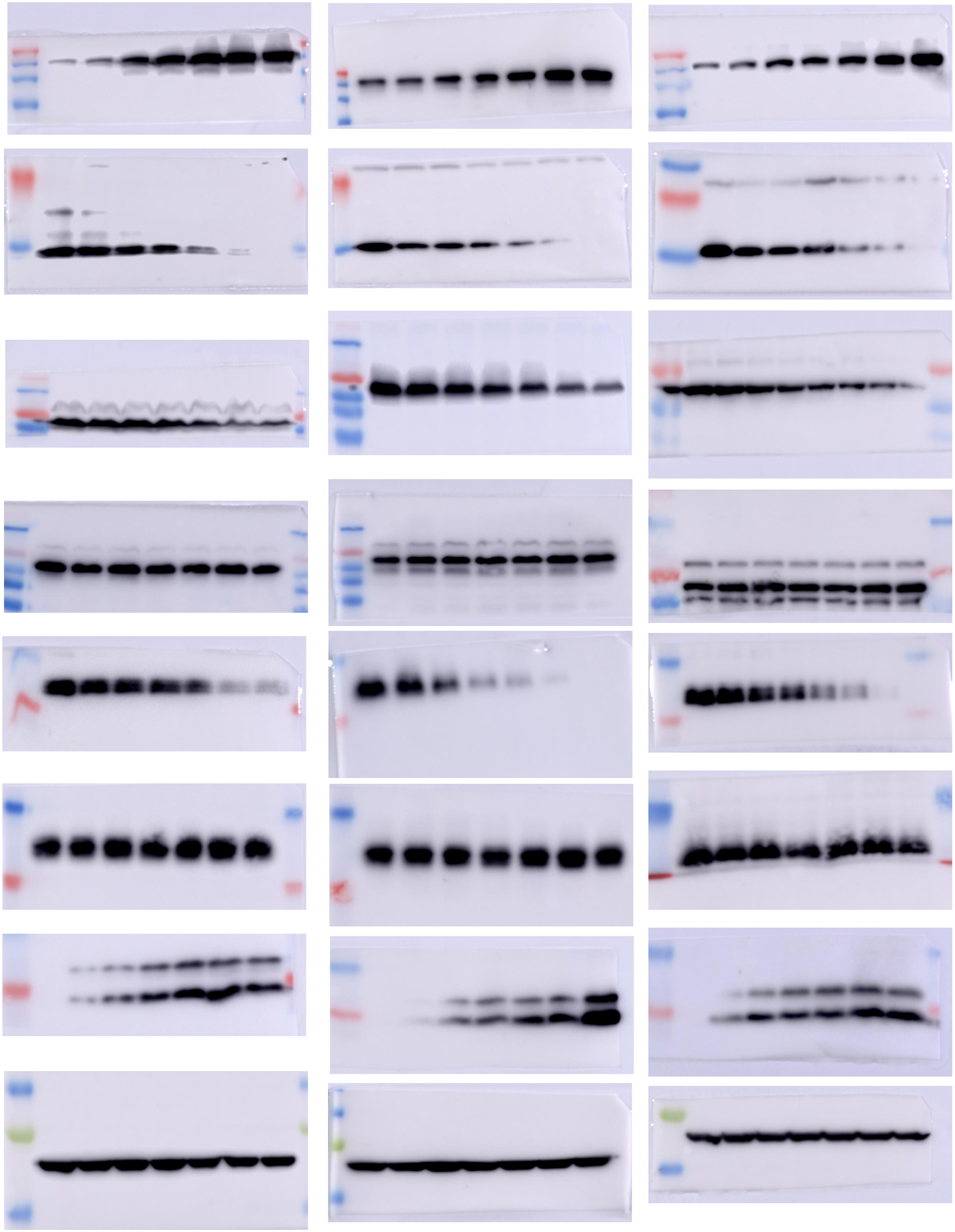

Figure 5G

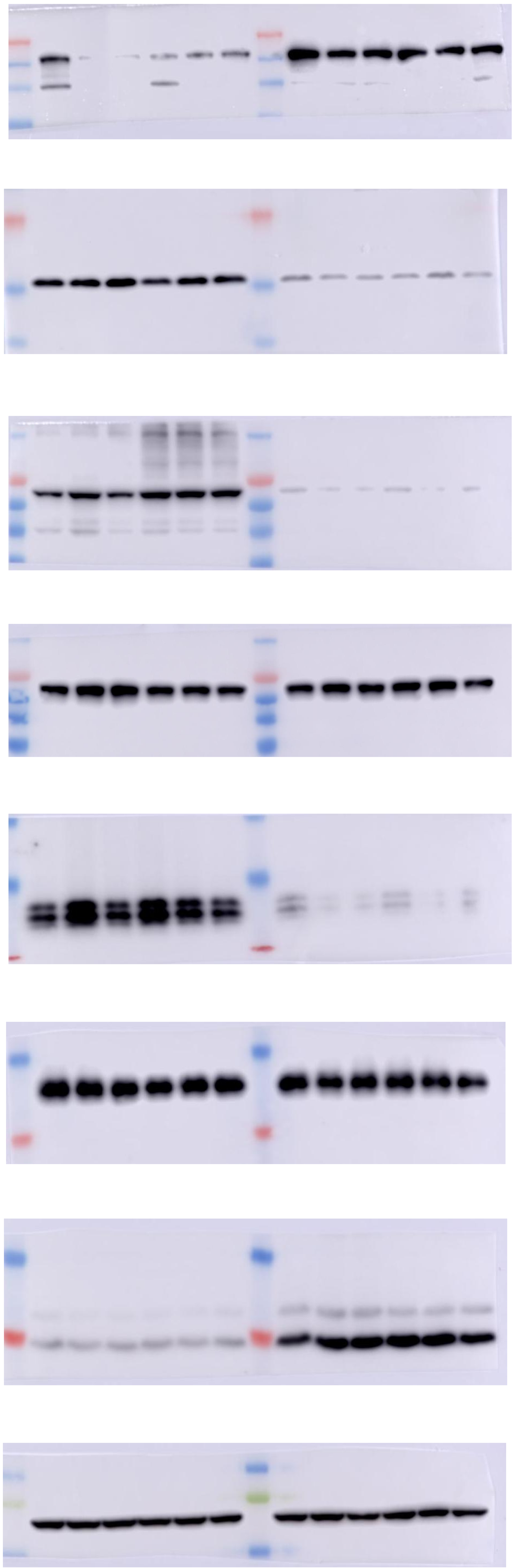

Figure 5H

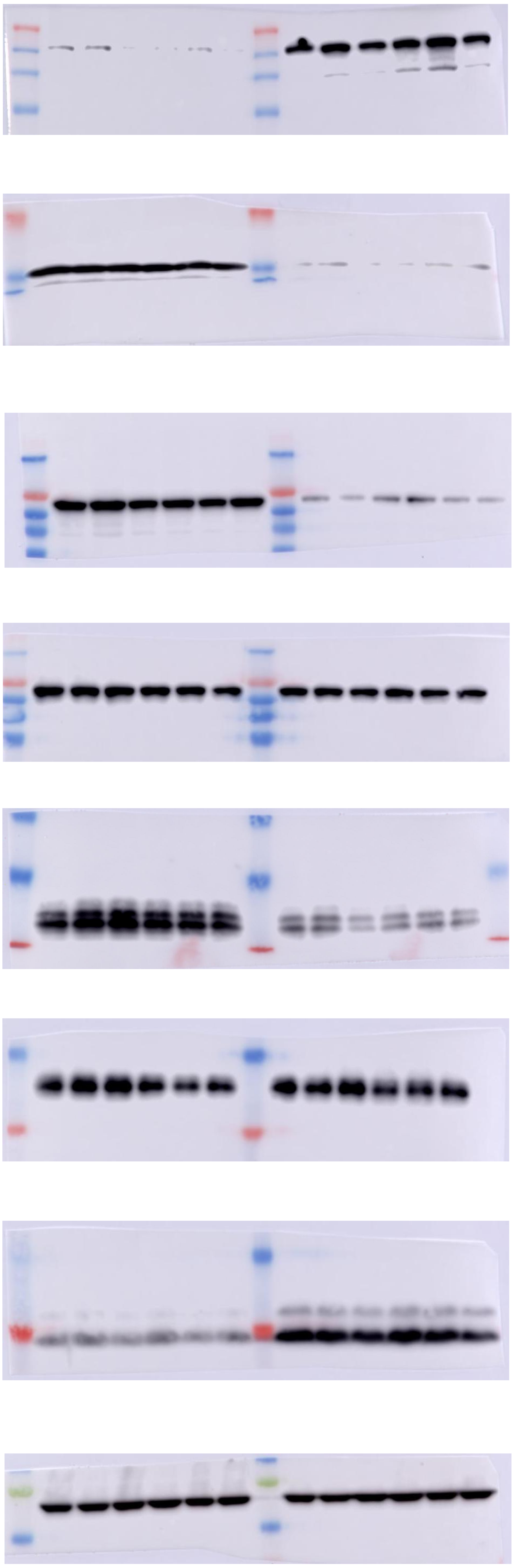

Figure 6A

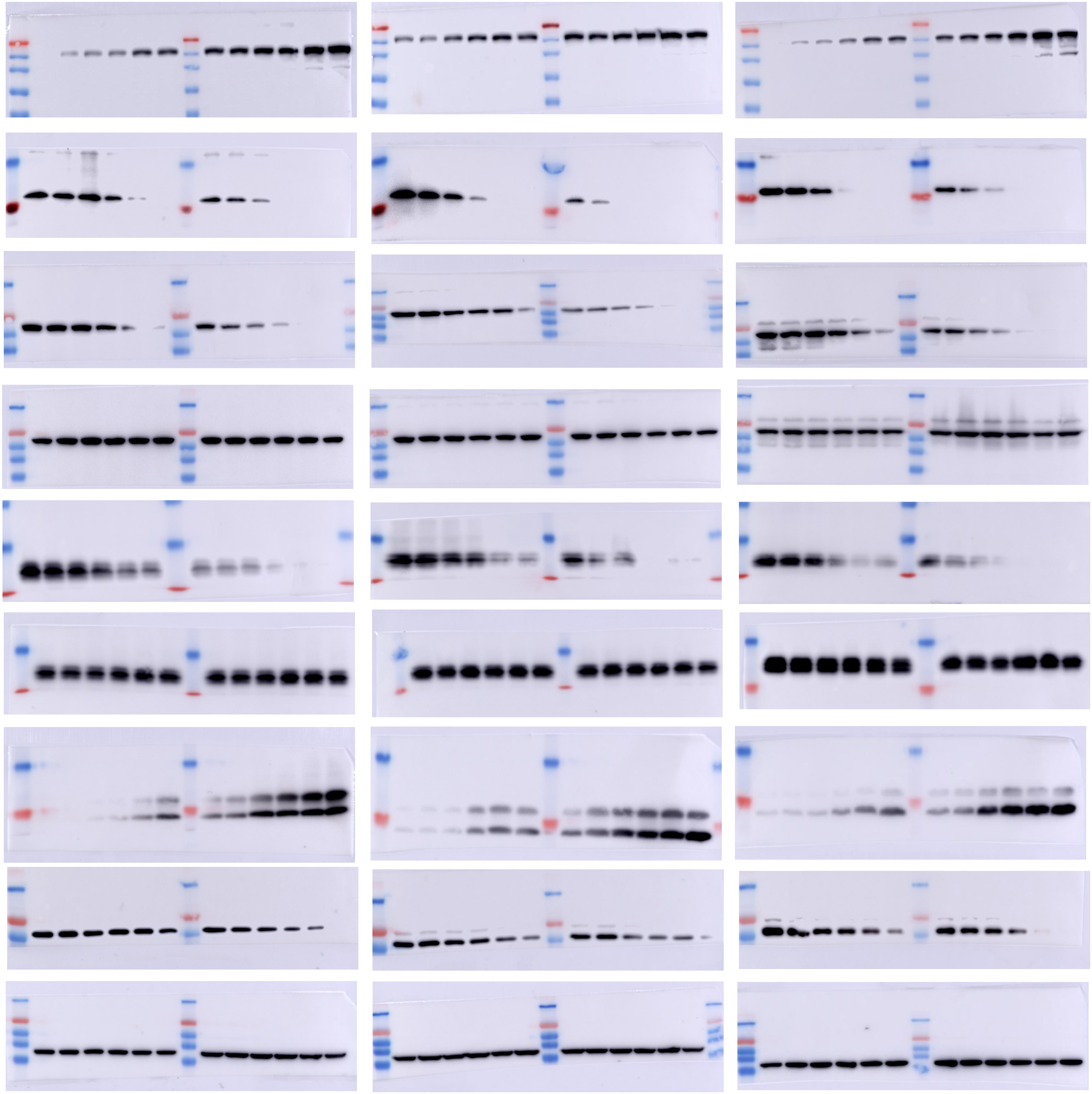

Figure 6D

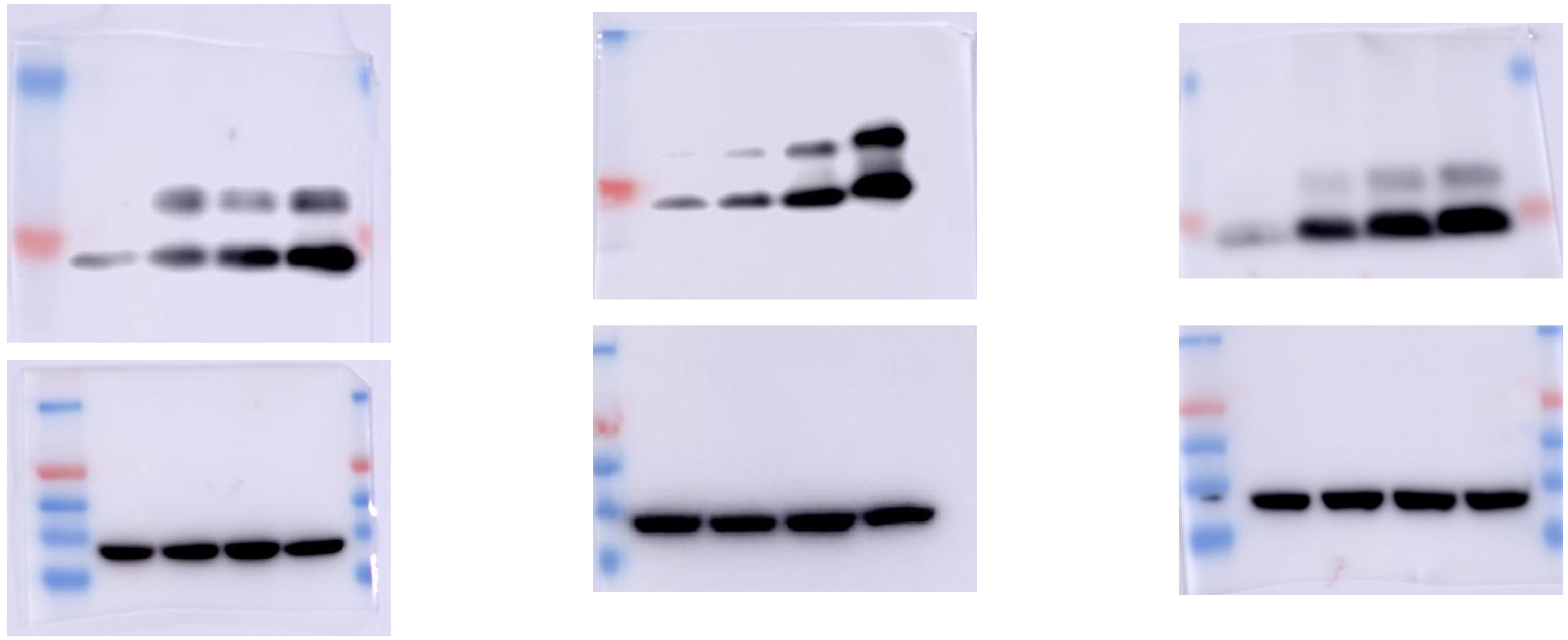

Figure 6E

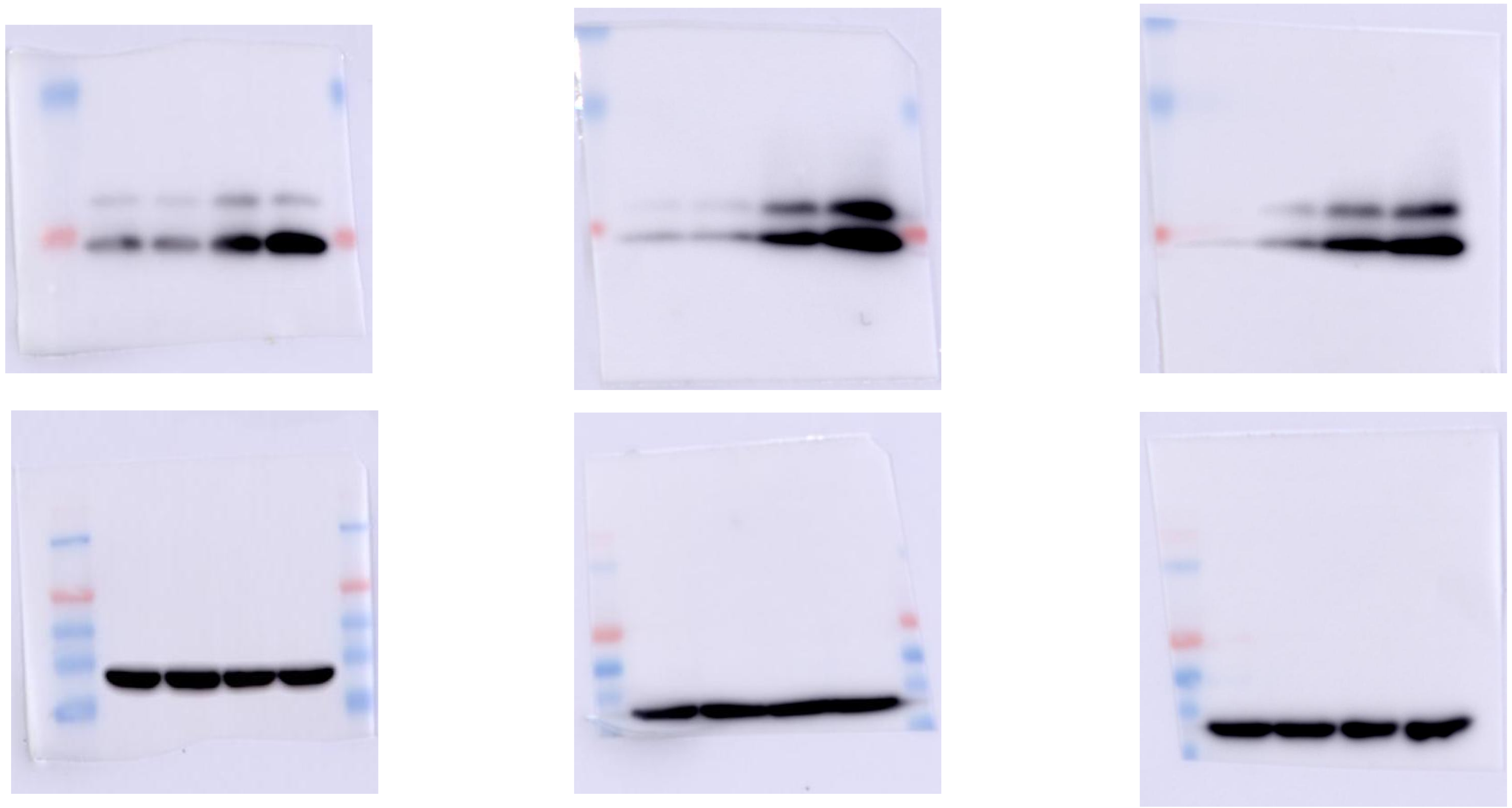

Figure 6F

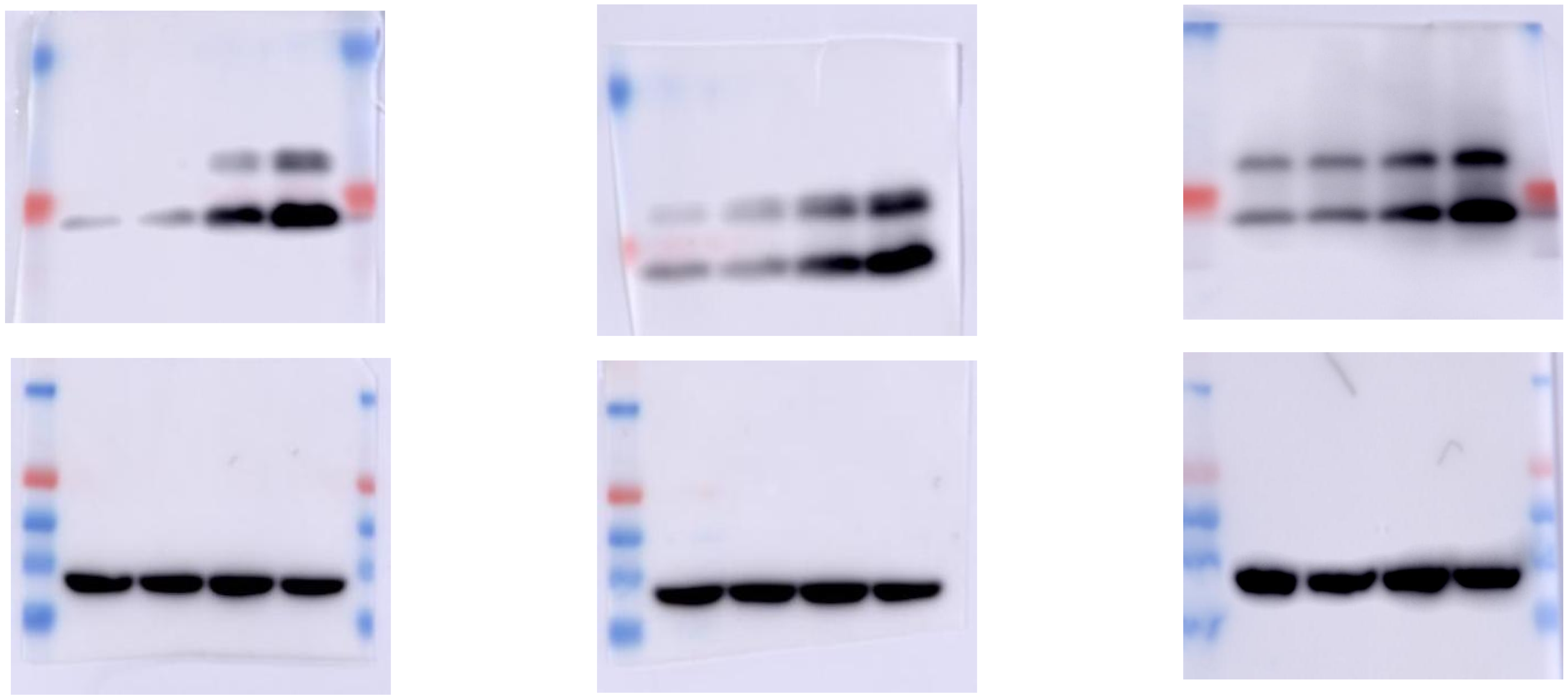

Figure 6G

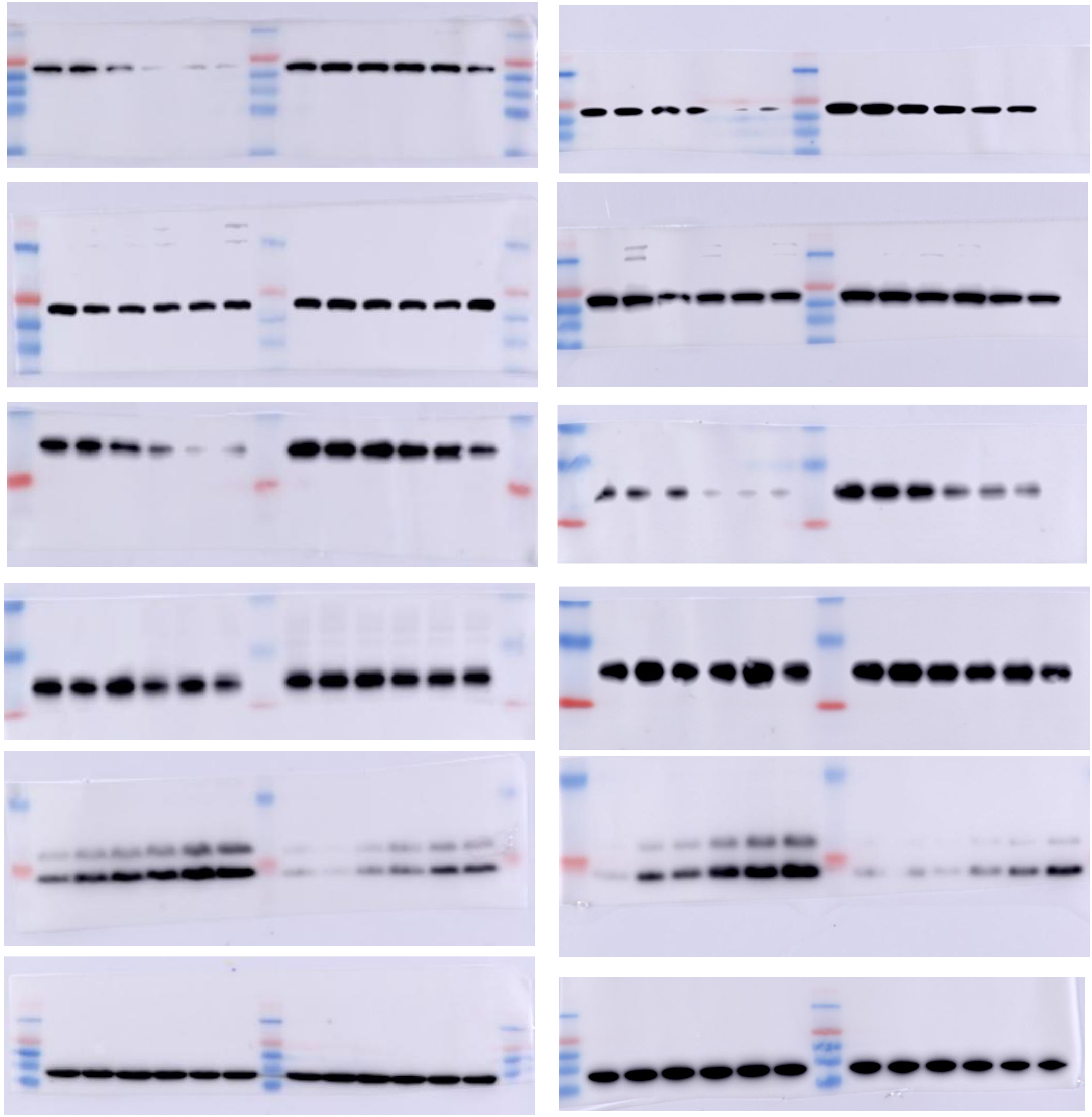

Figure 7D

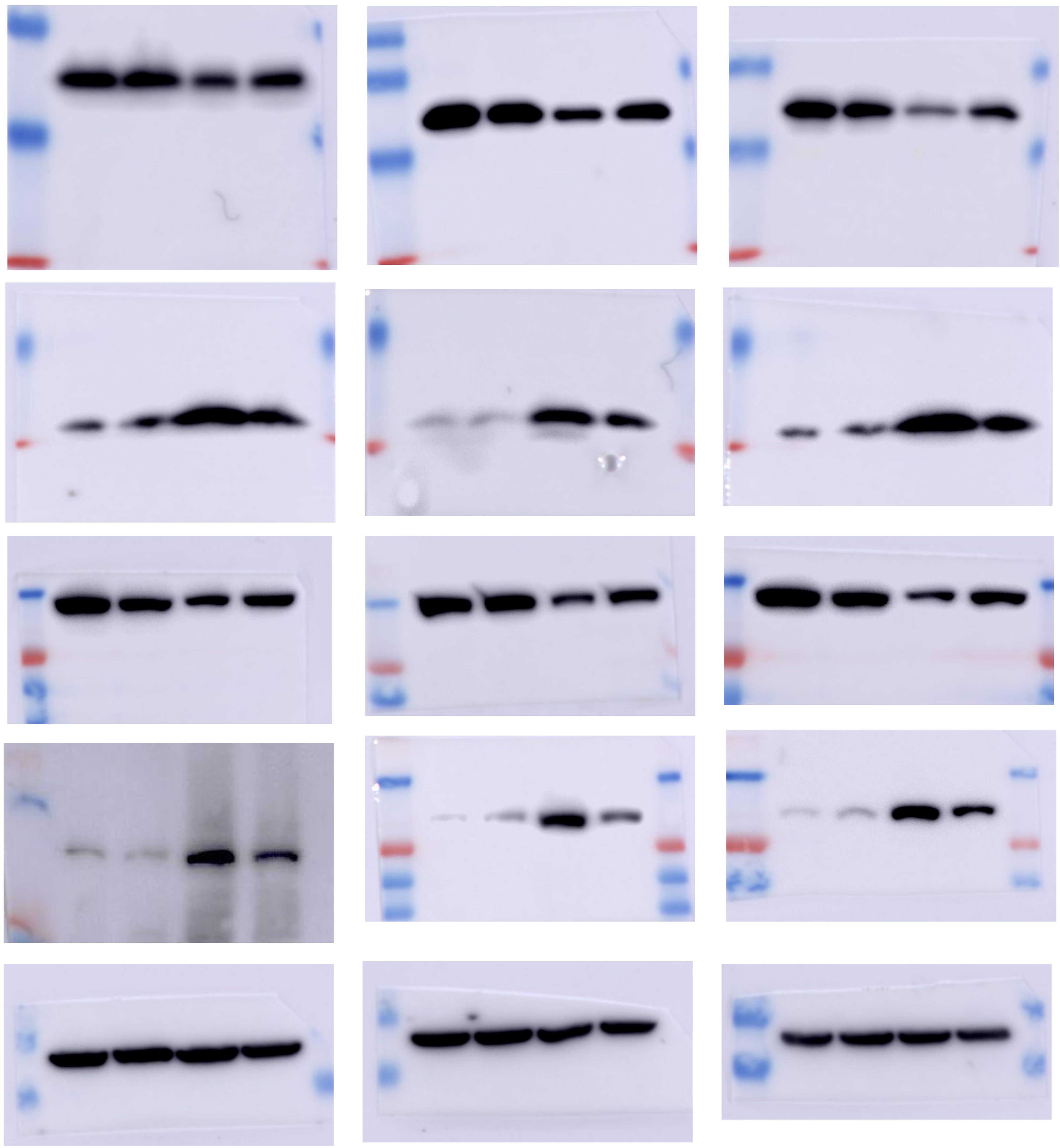

Figure 7F

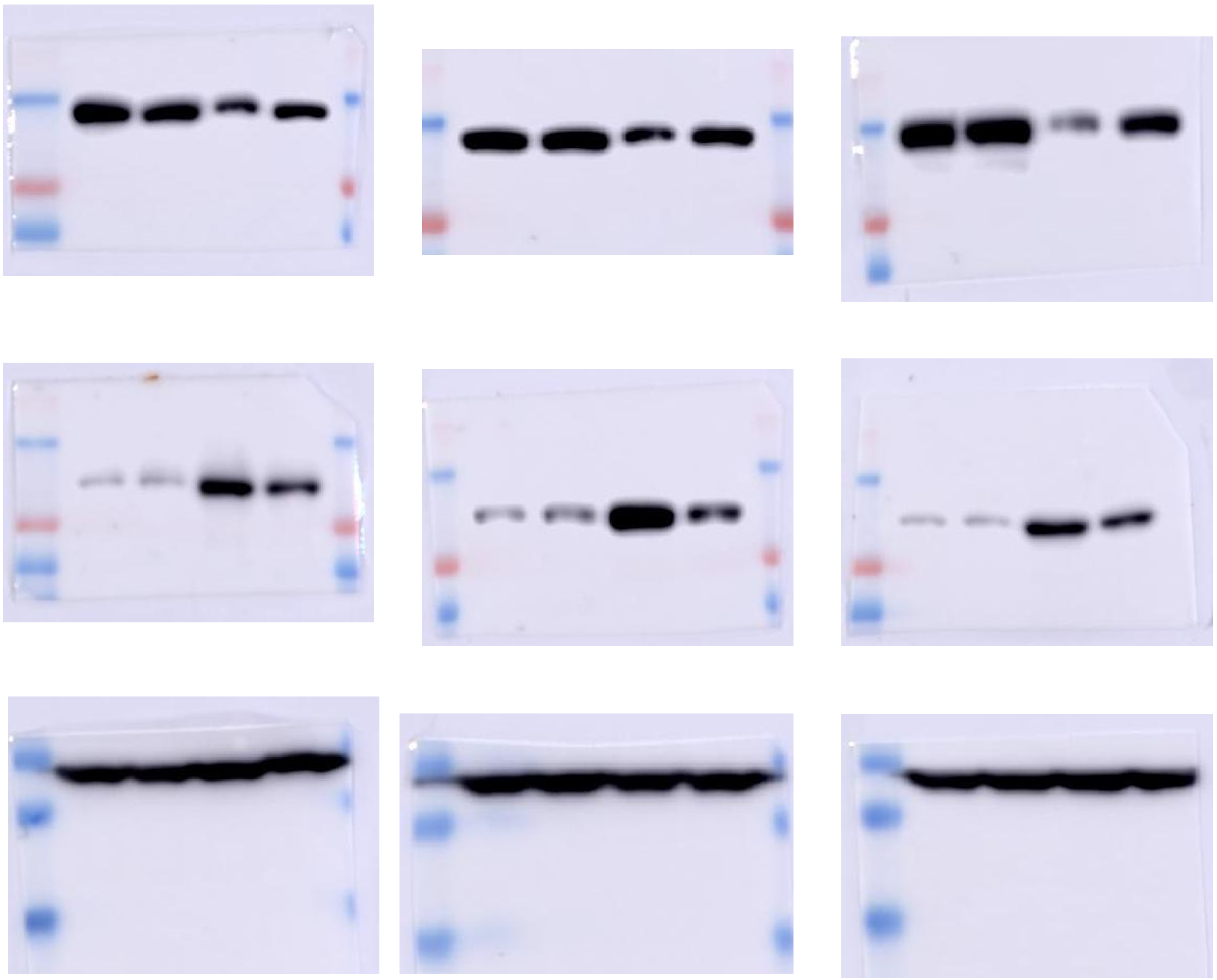

Figure 7H

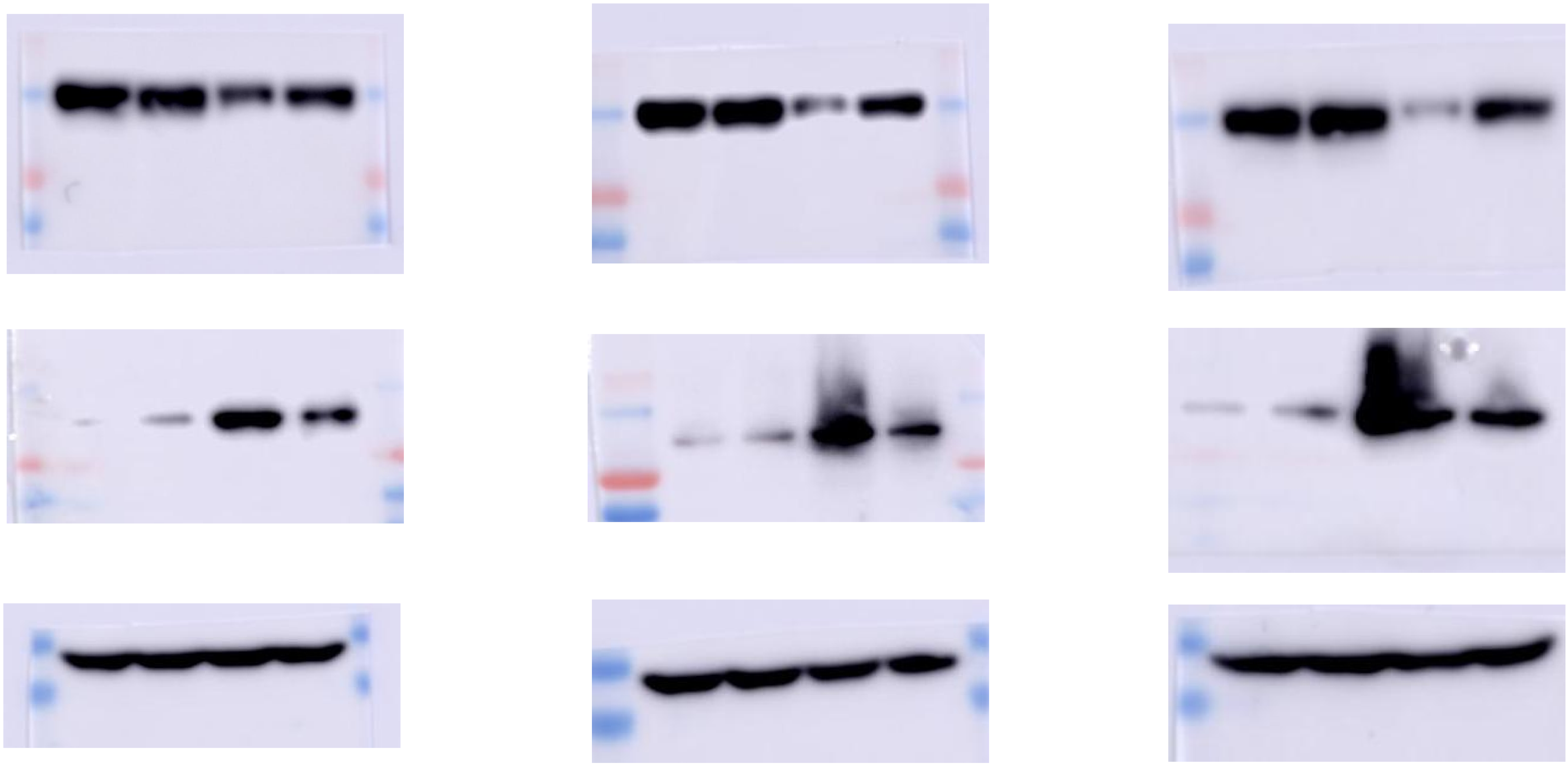

Figure 7J

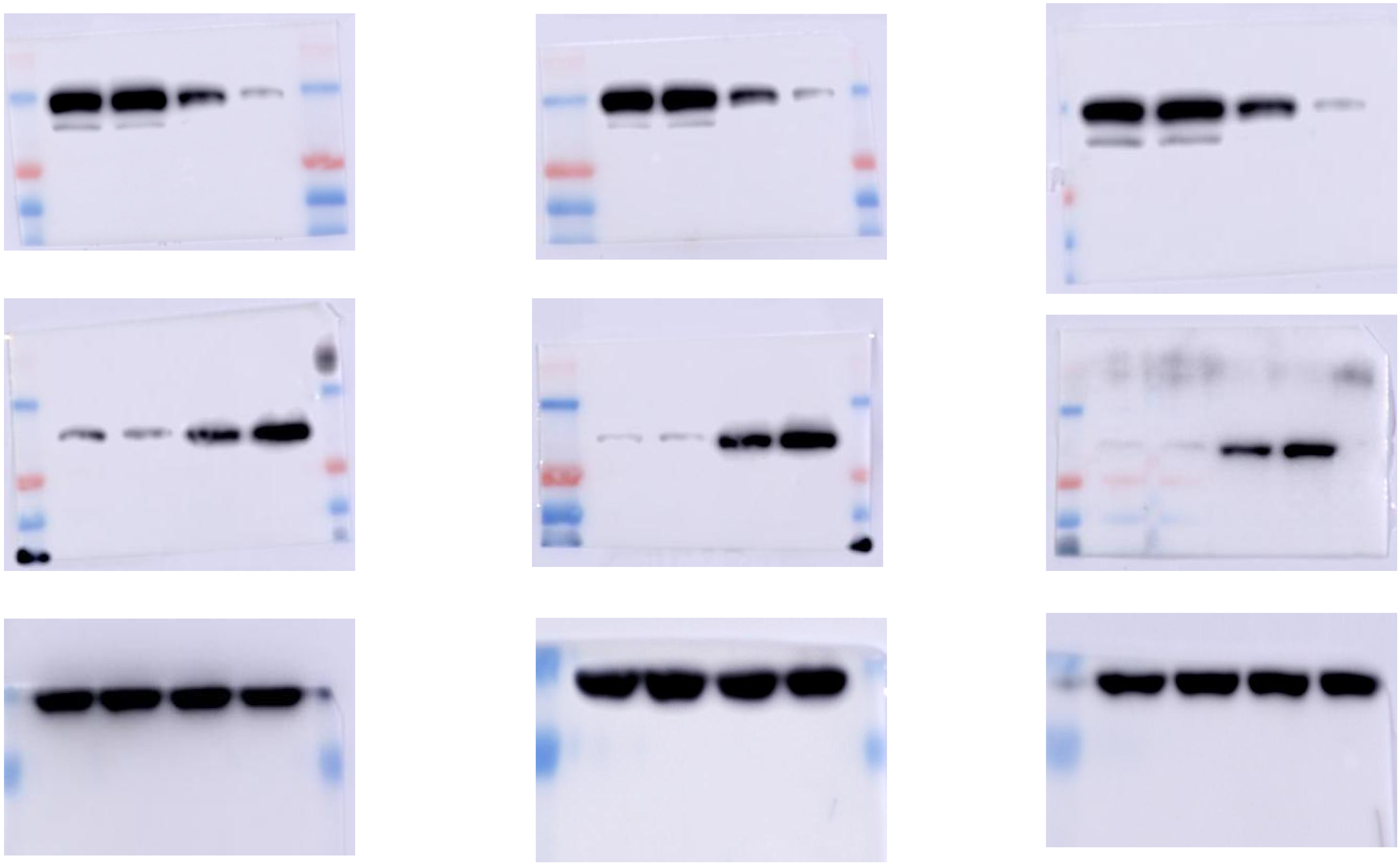

Supplementary Figure

Figure S1A

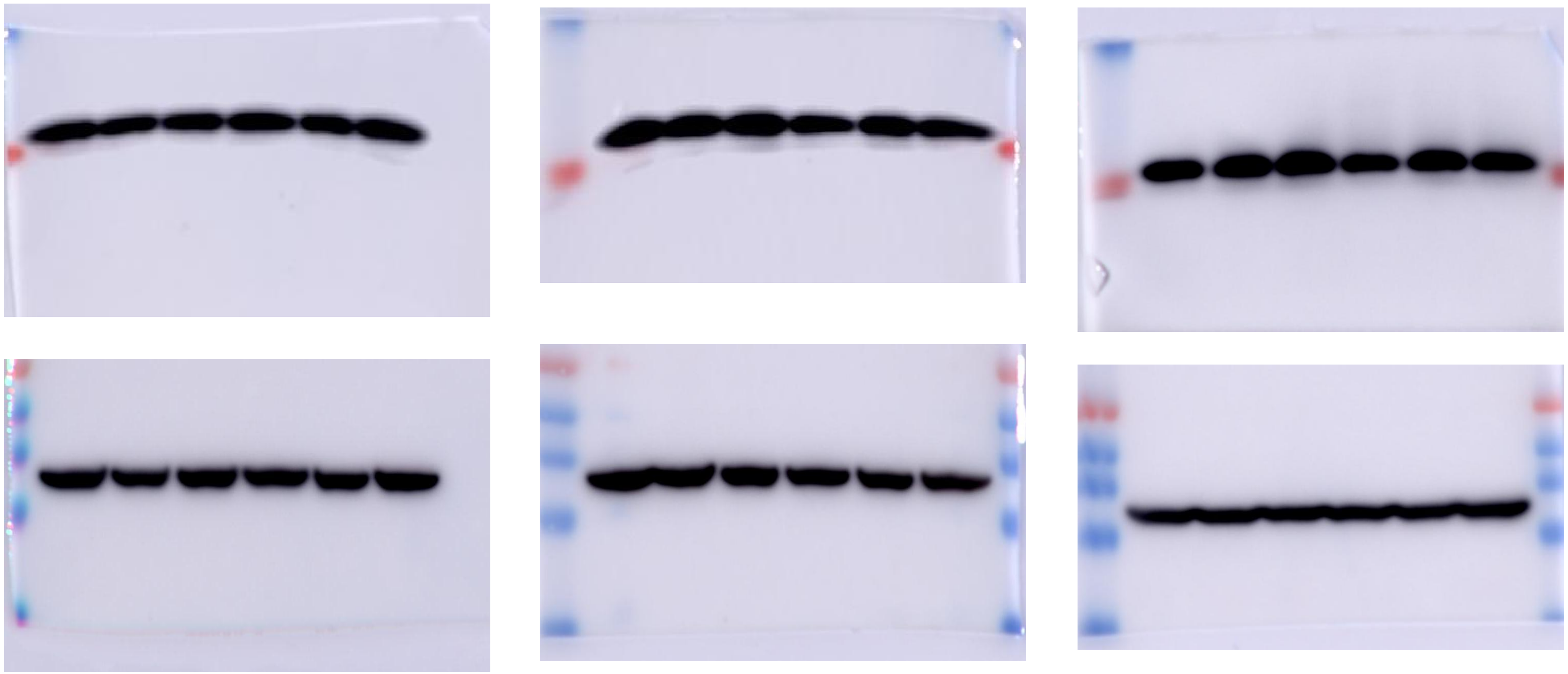

Figure S1C

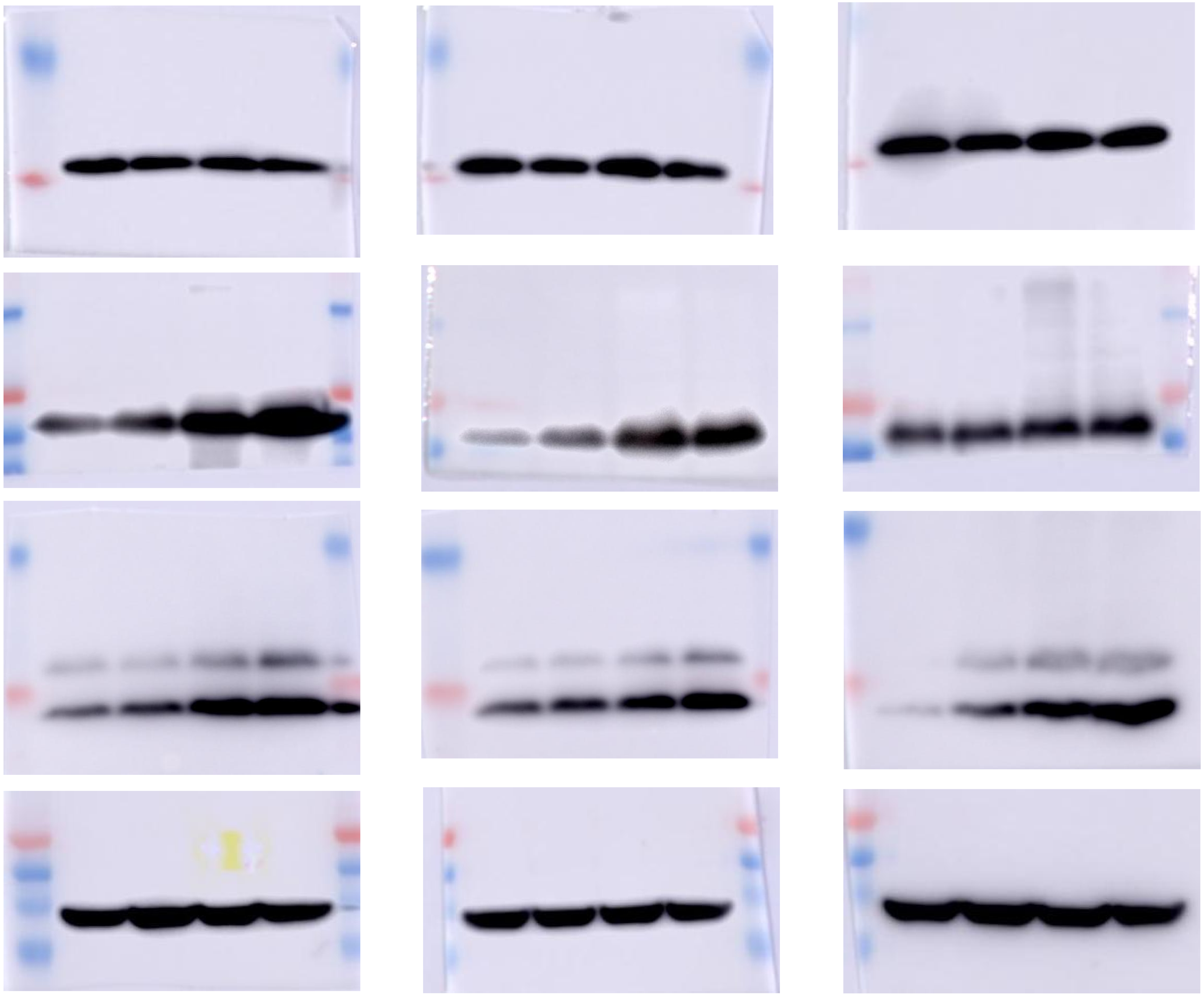

Figure S2F

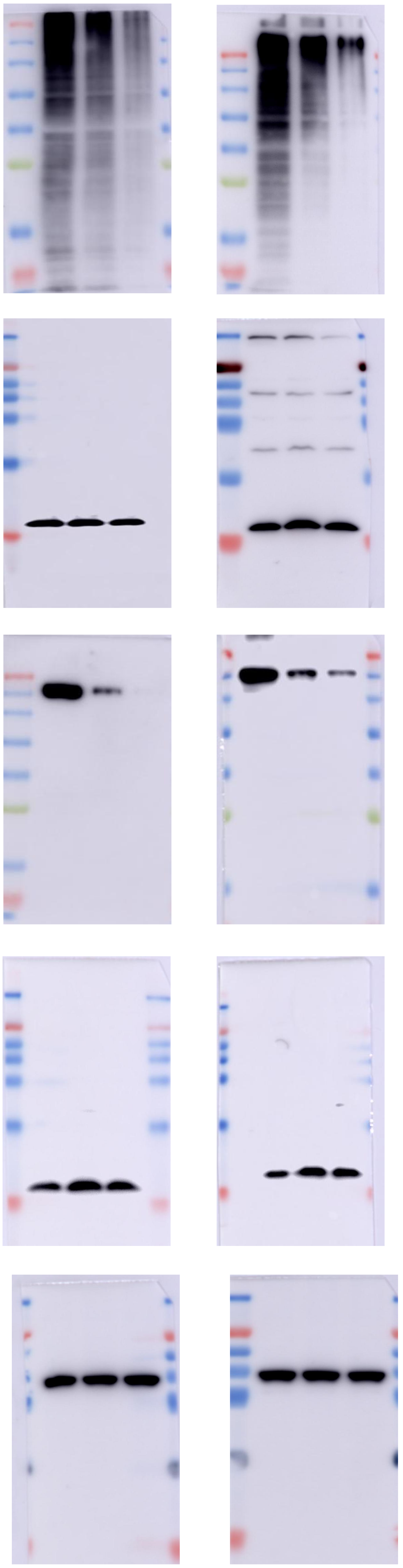

Figure S2G

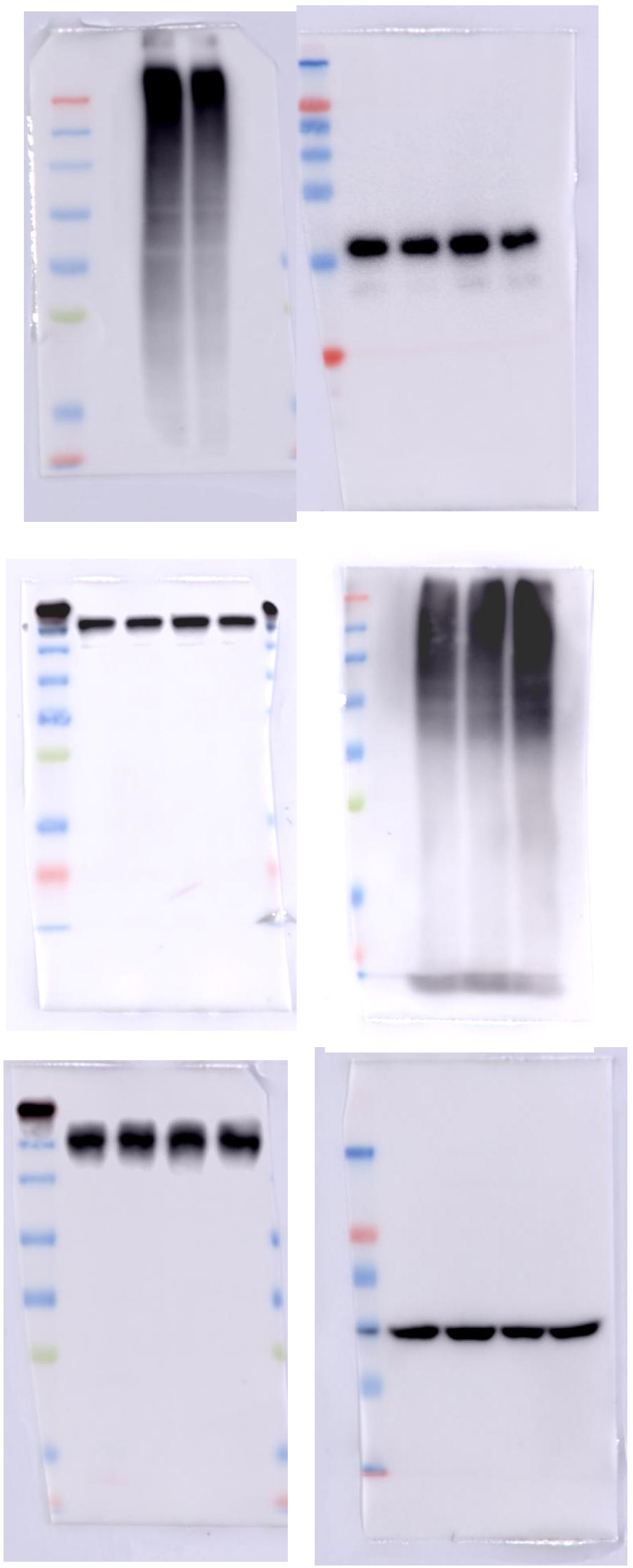

Figure S2H

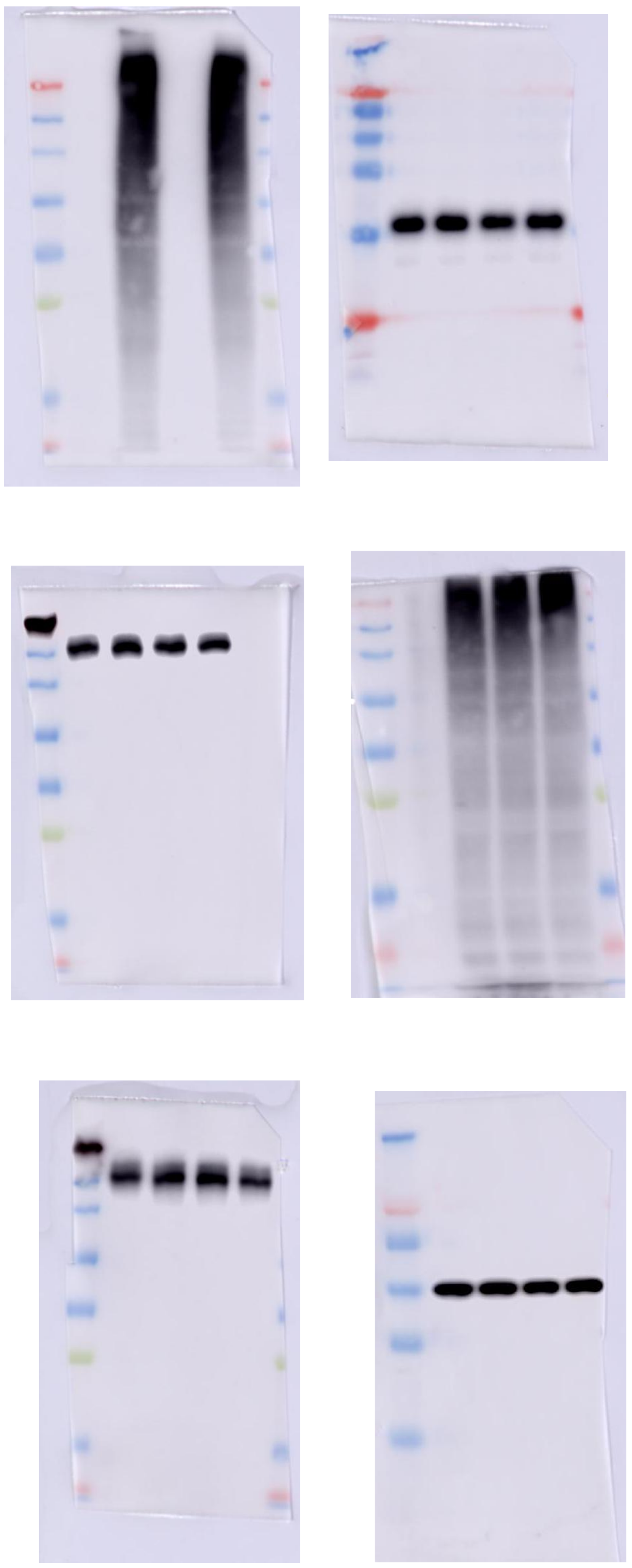

Figure S3A

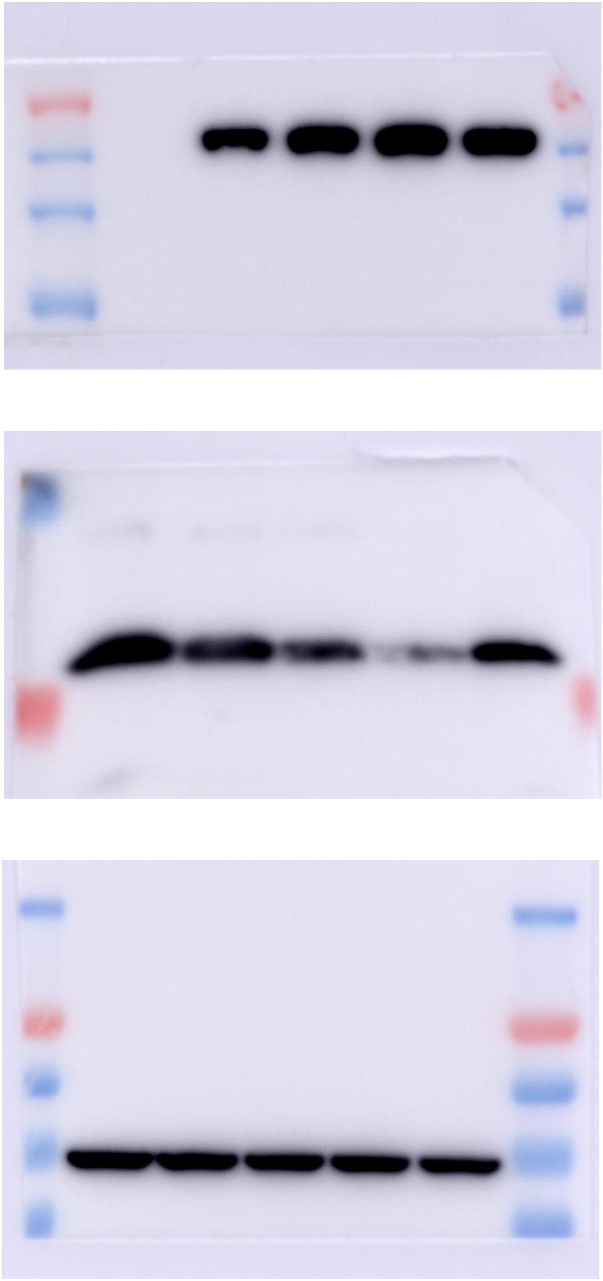

Figure S3B

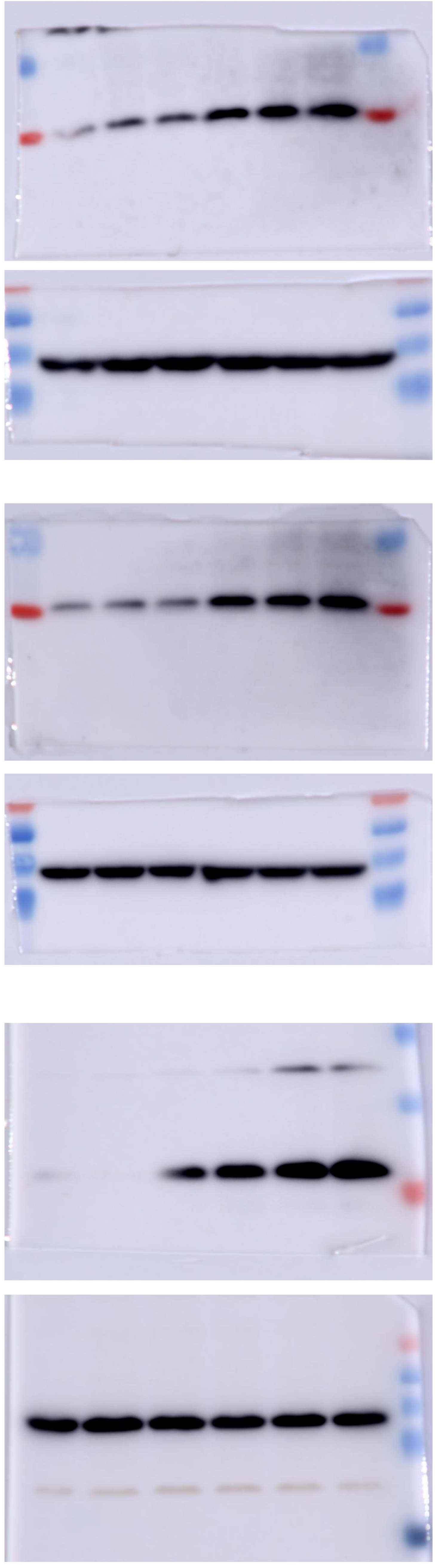

Figure S3C

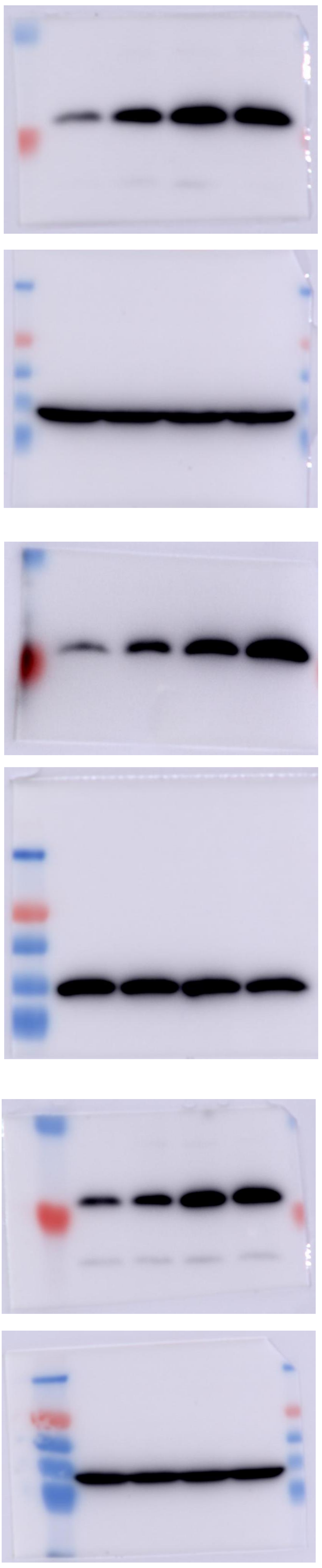

Figure S3D

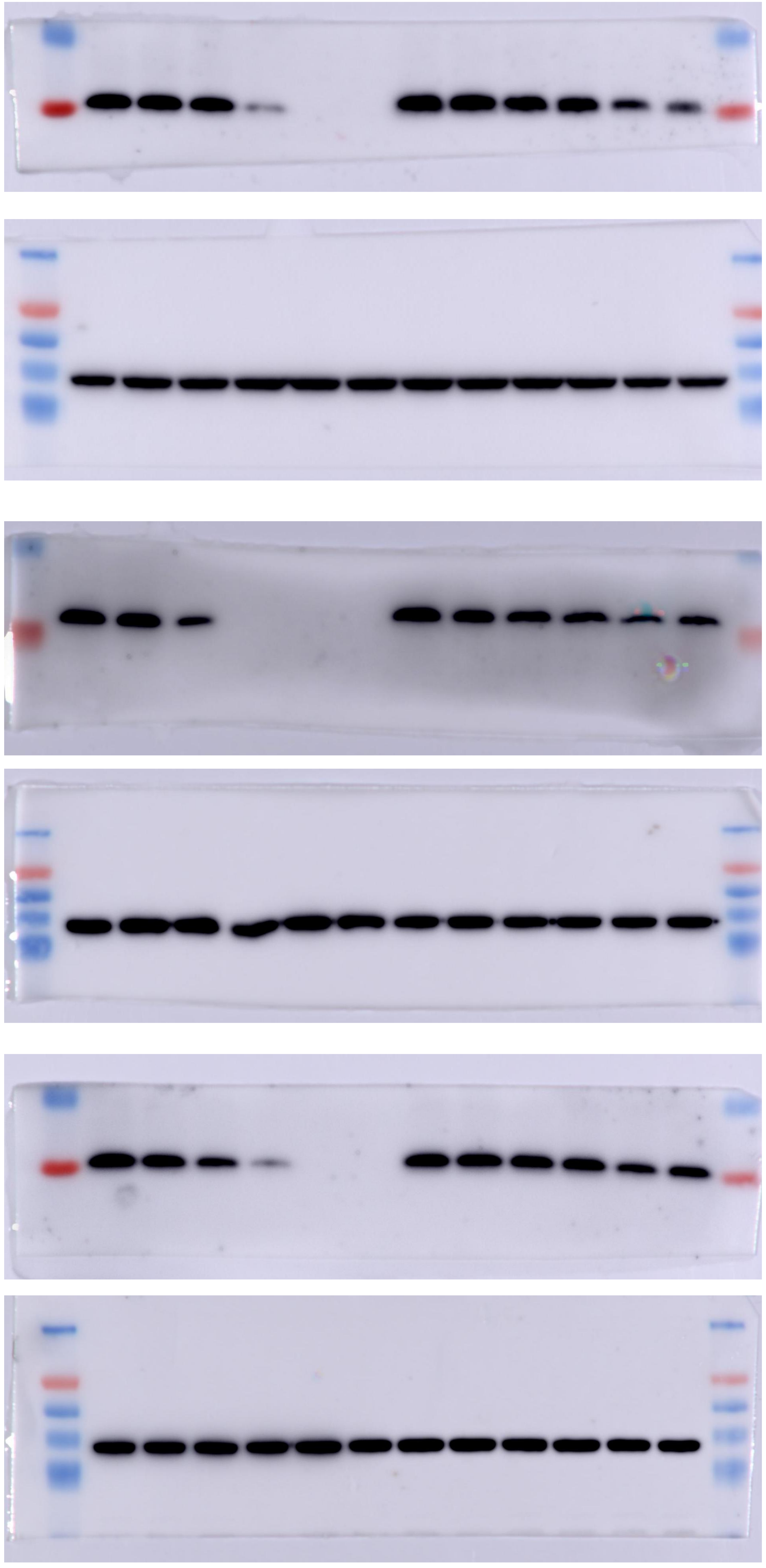

Figure S3E

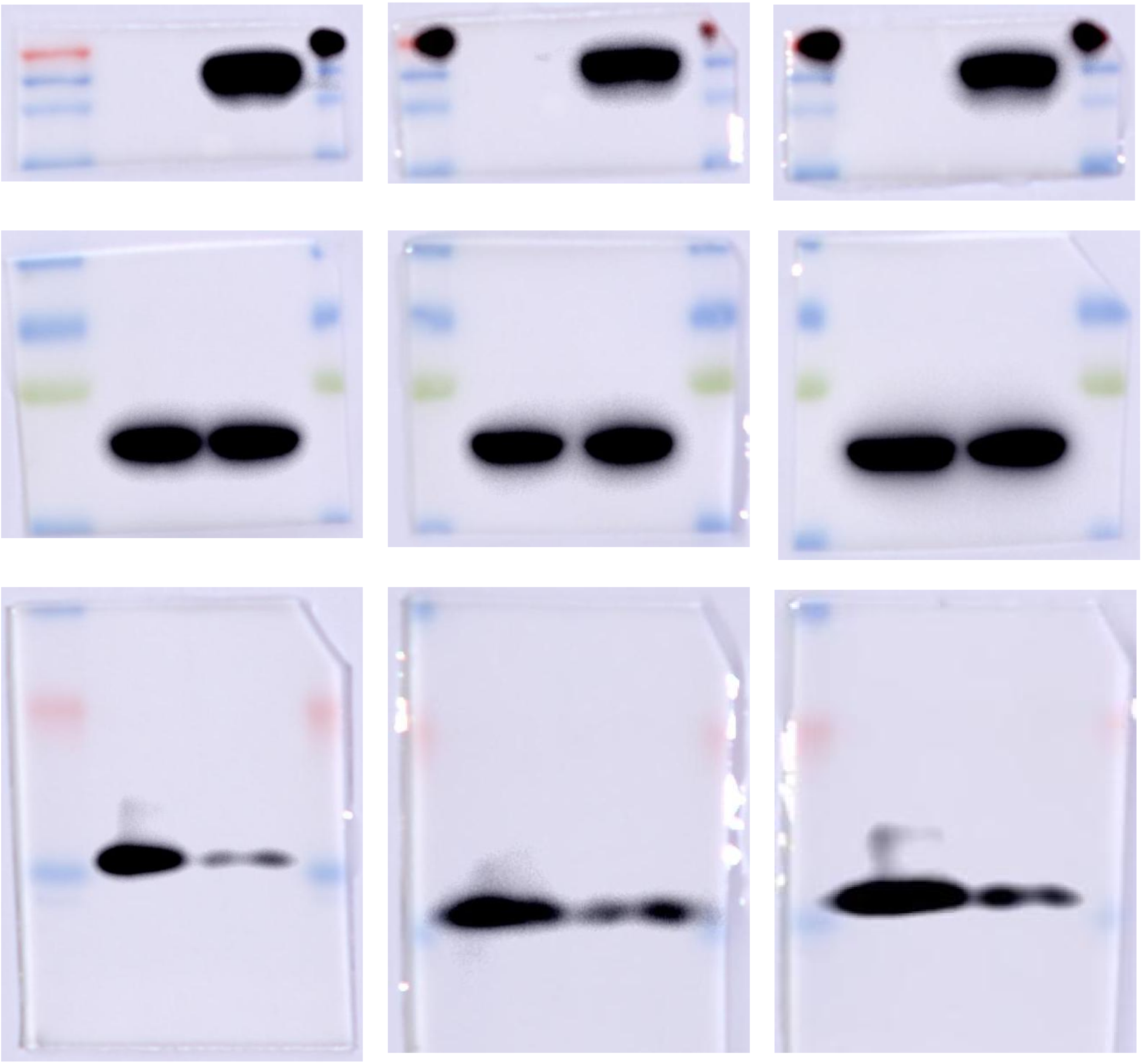

Figure S4A

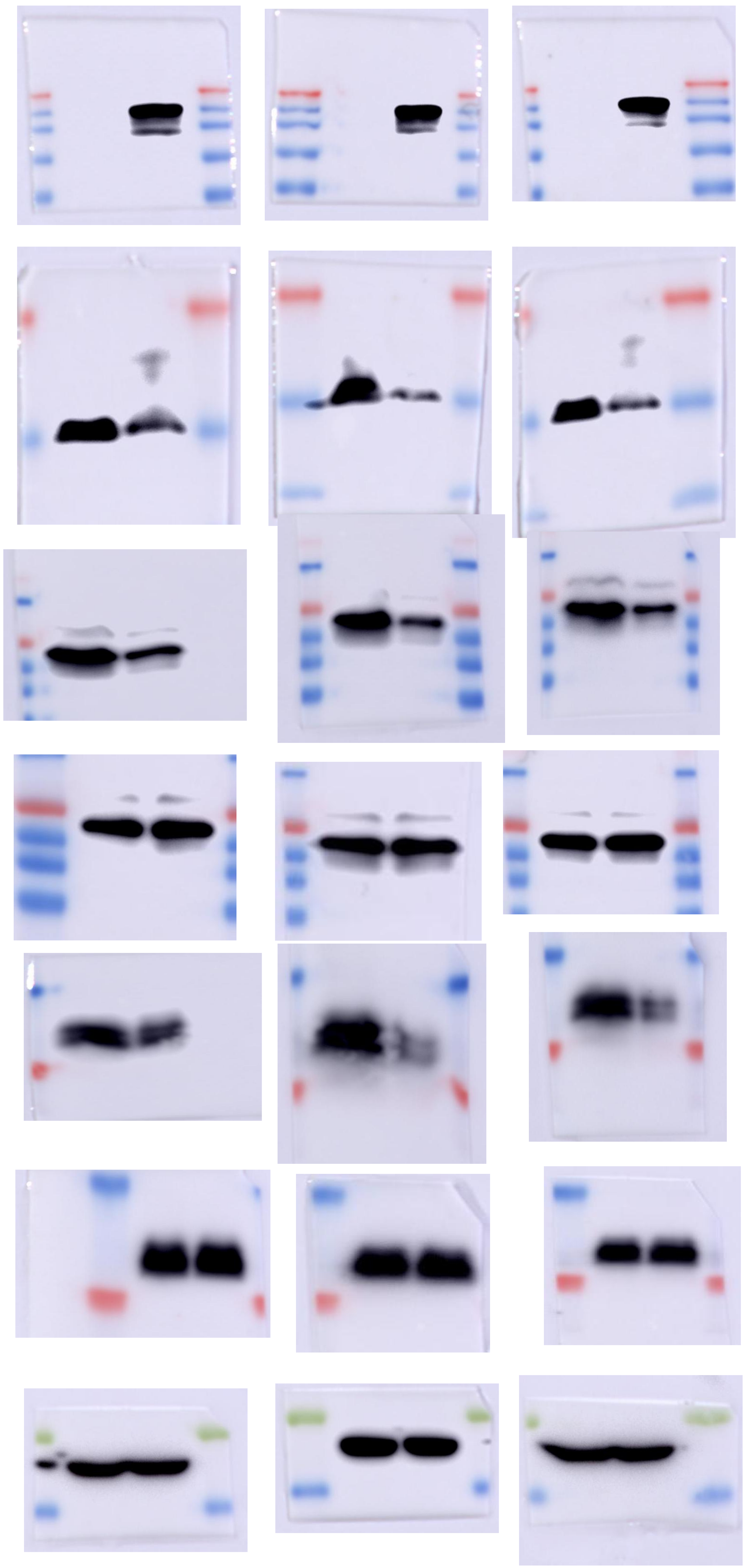

Figure S4B

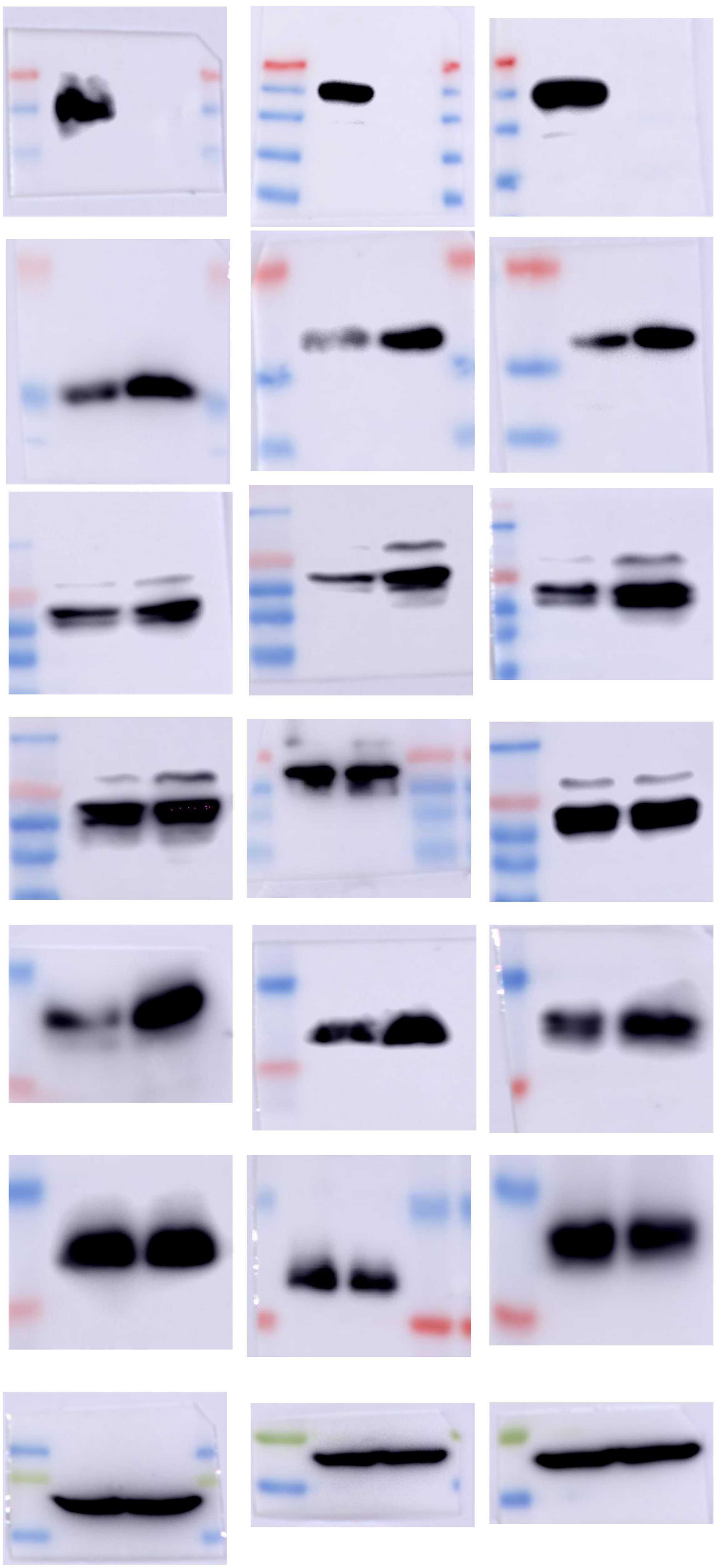

Figure S4C

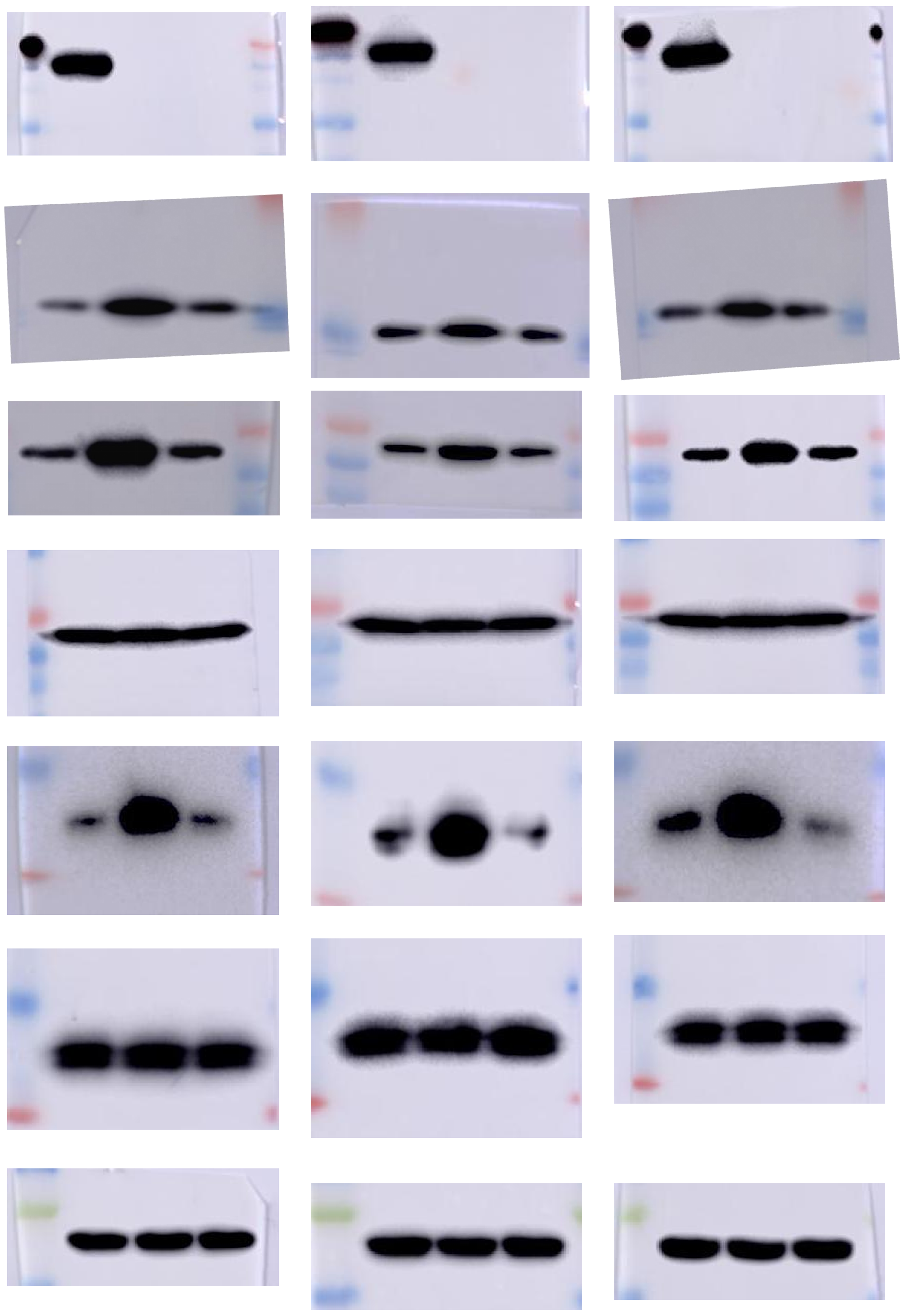

Figure S4D

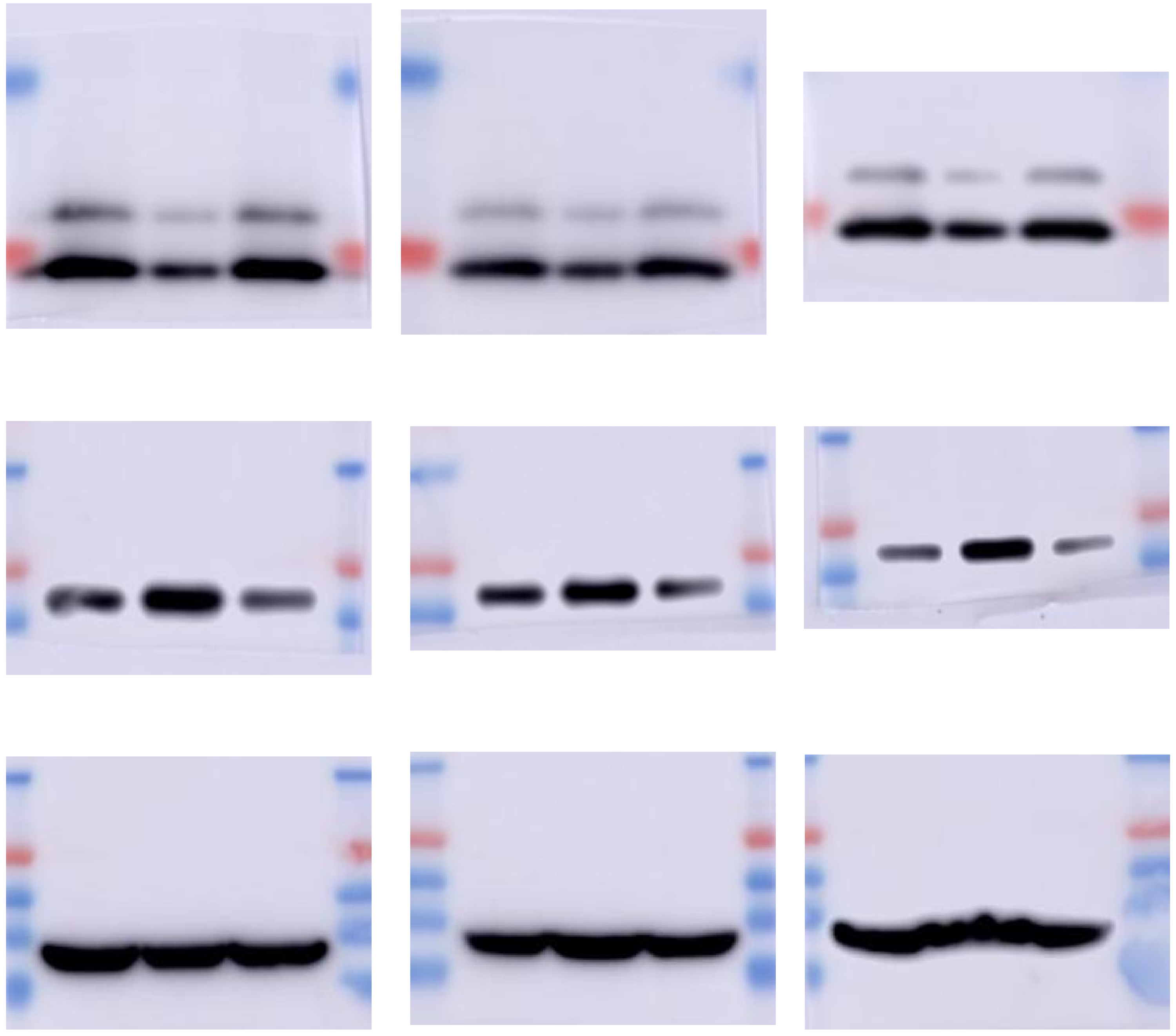

Figure S5A

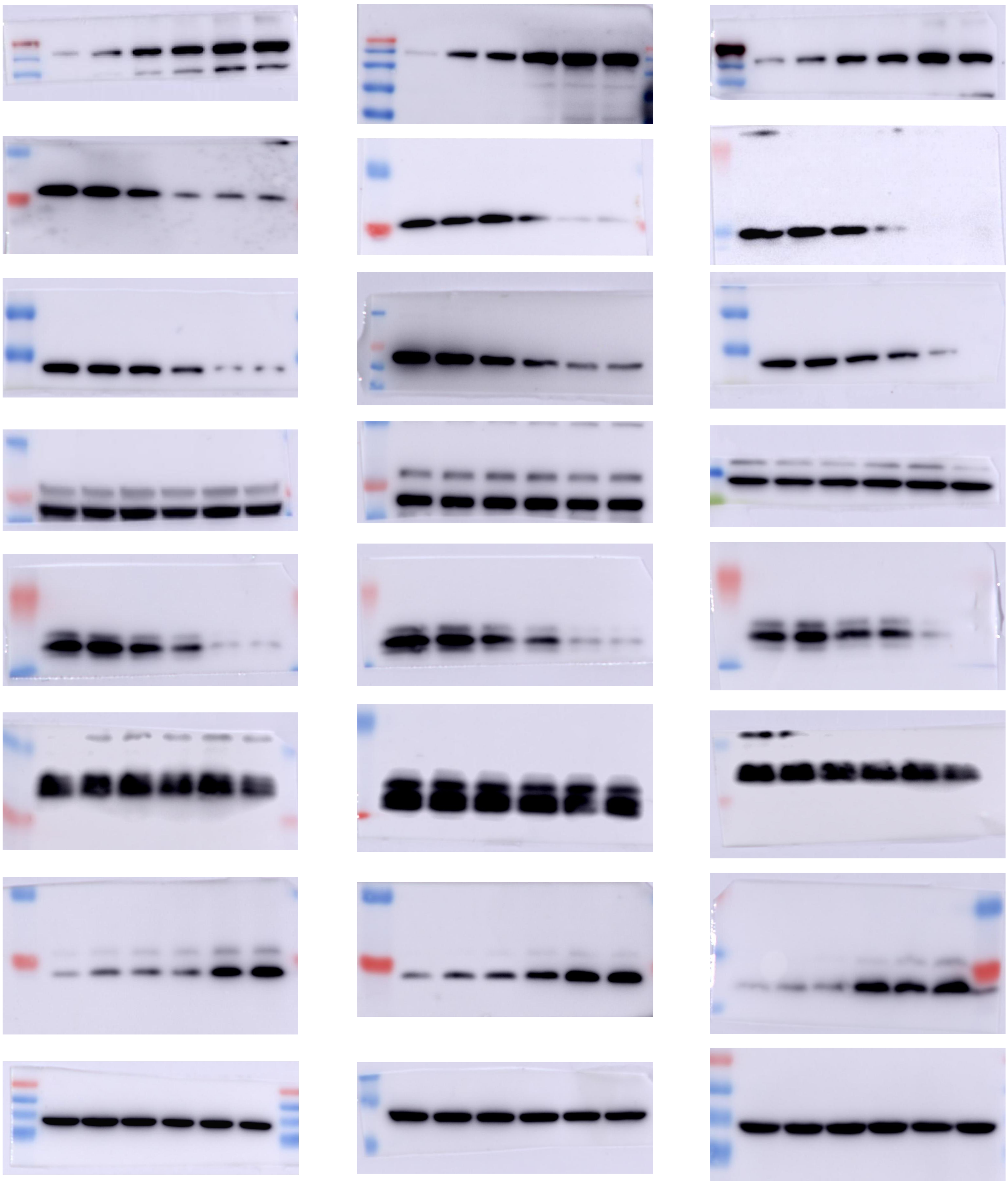

Figure S5B

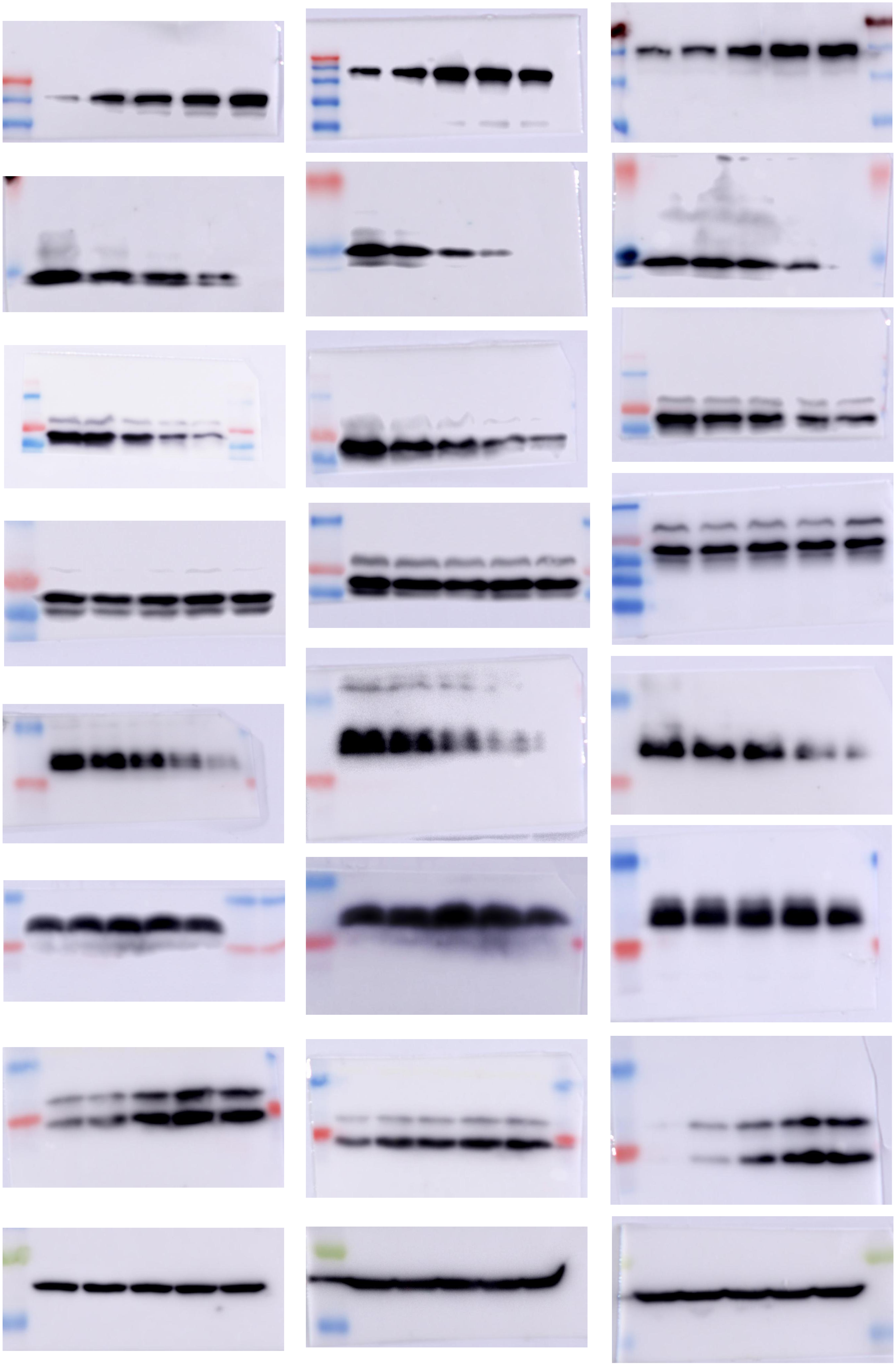

Figure S5C

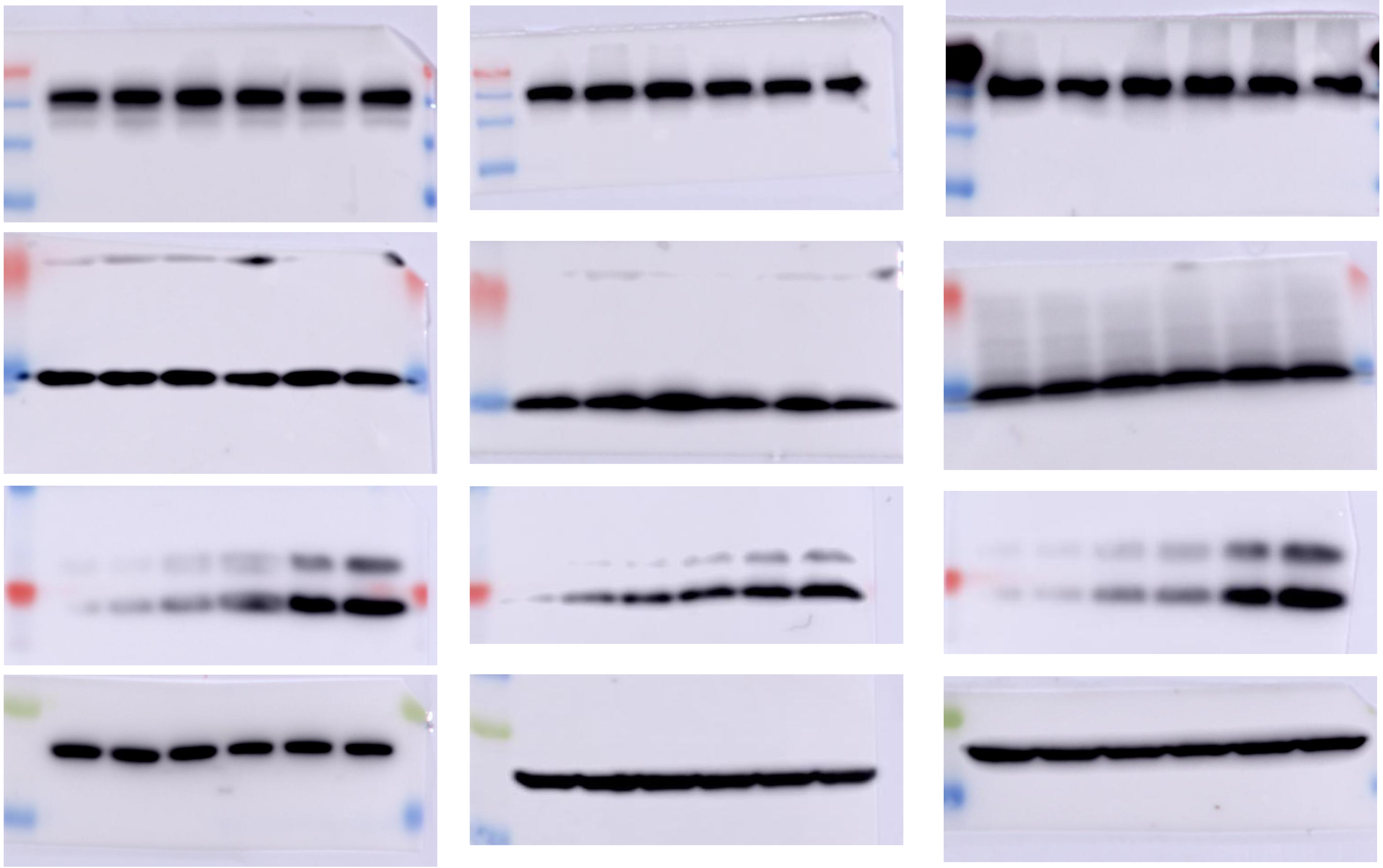

Figure S5D

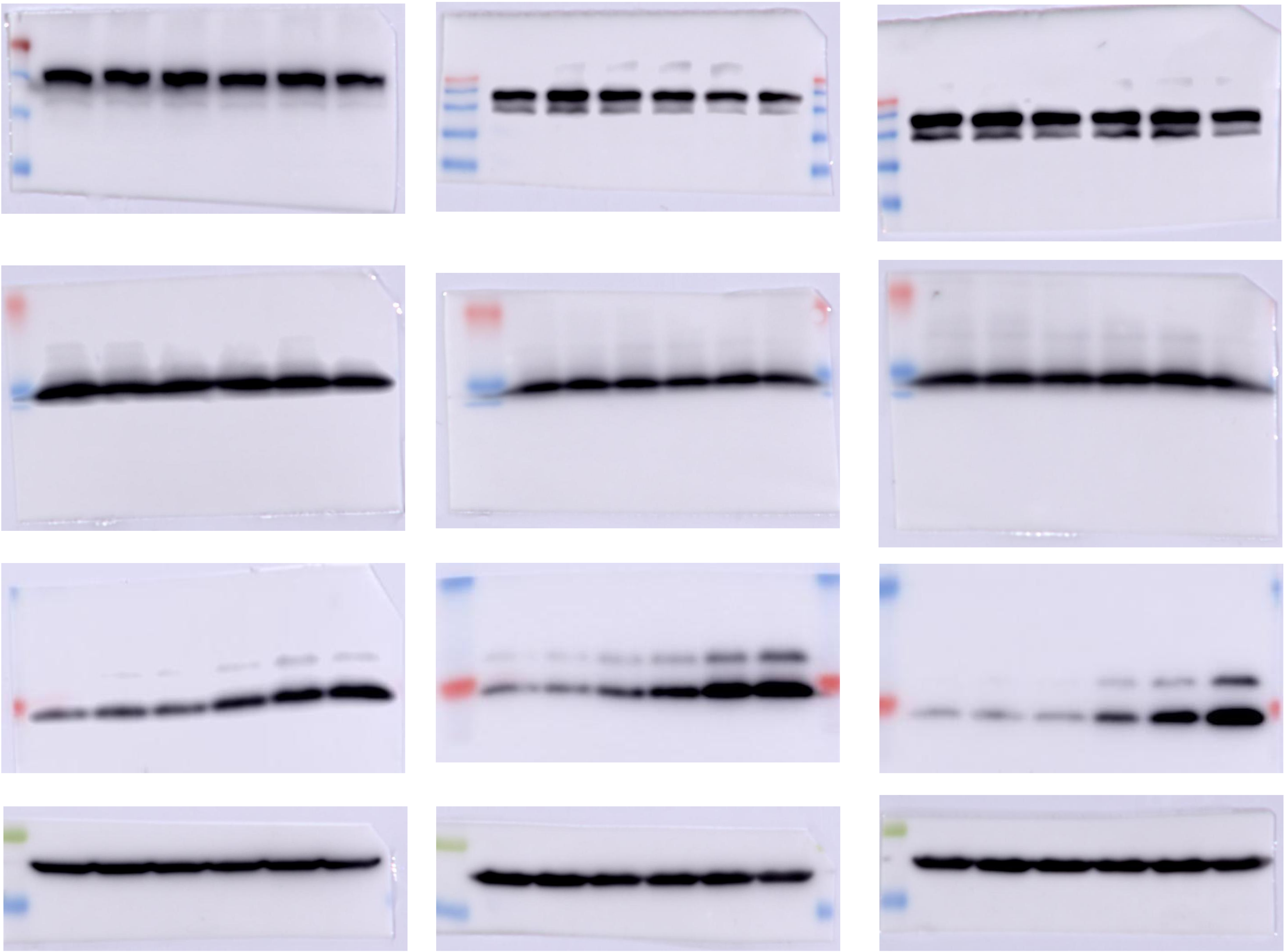

Figure S5F

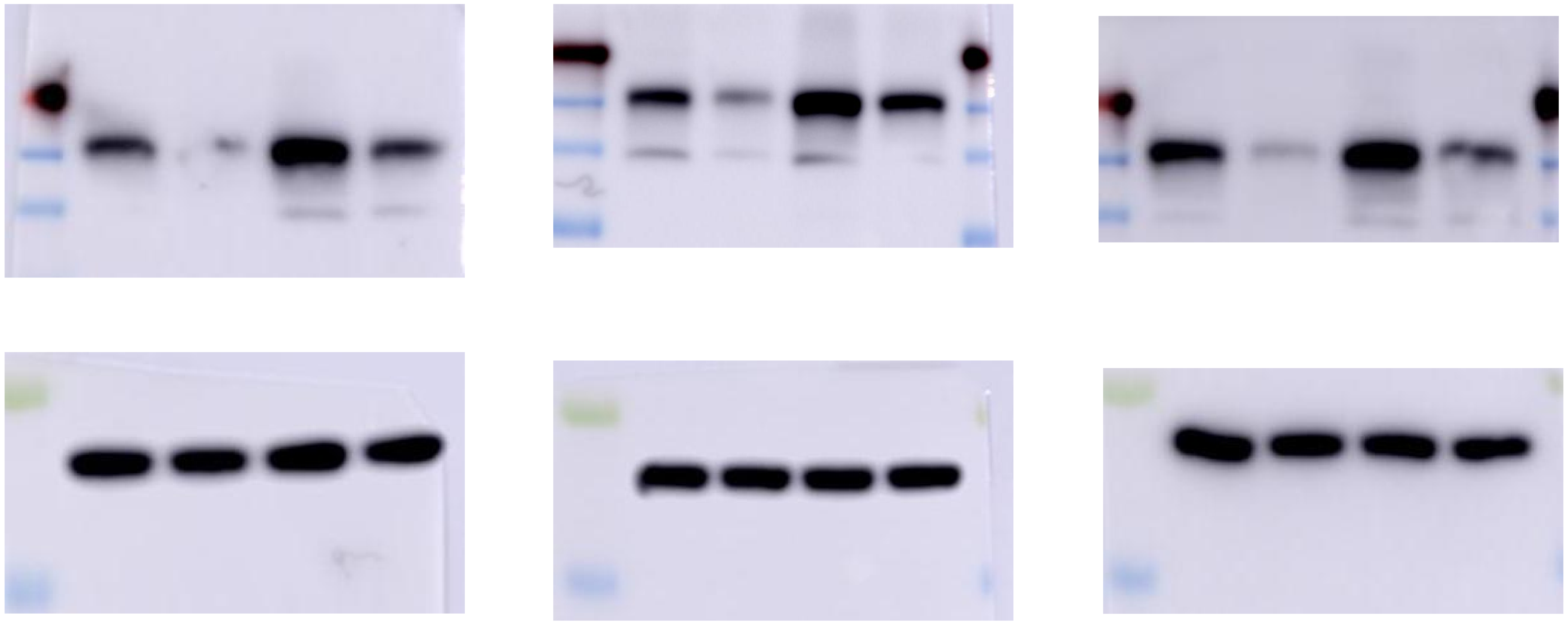

Figure S5G

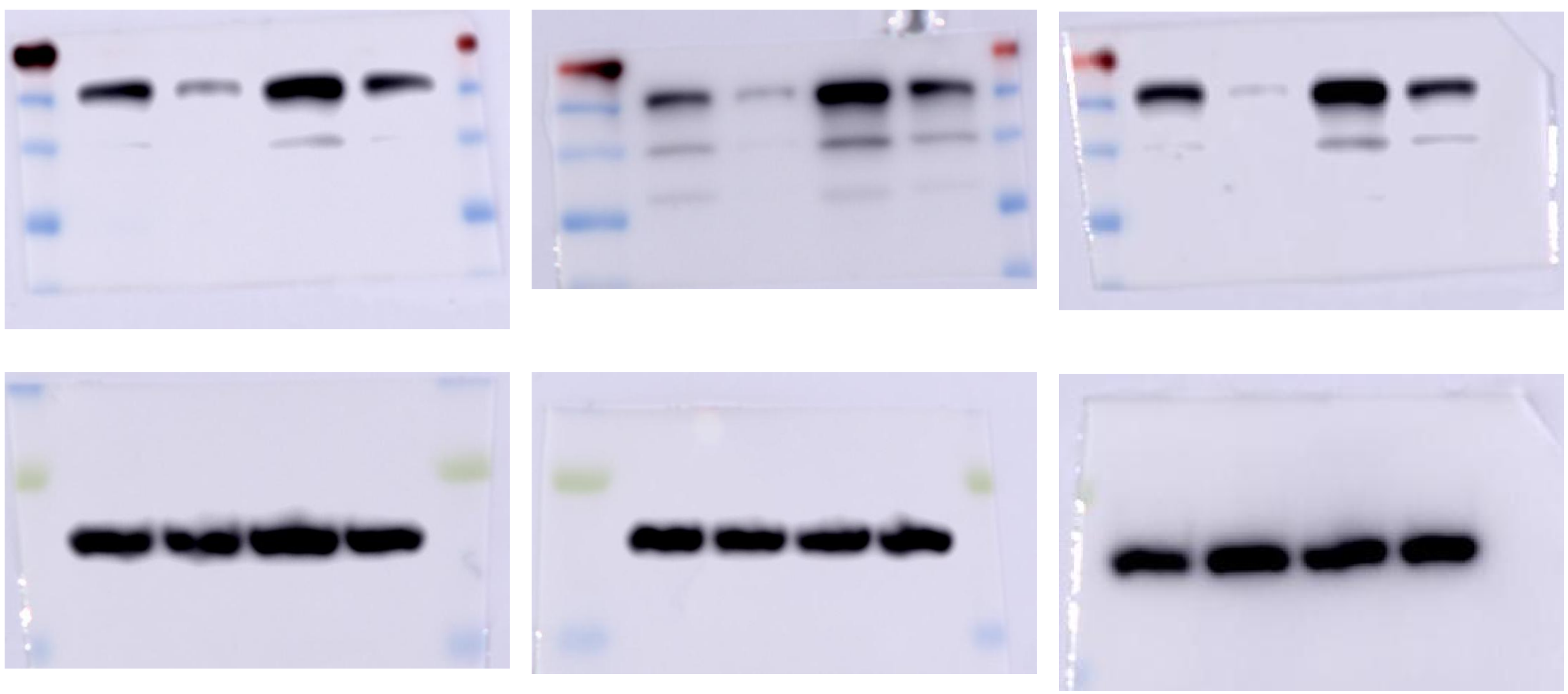

Figure S5H

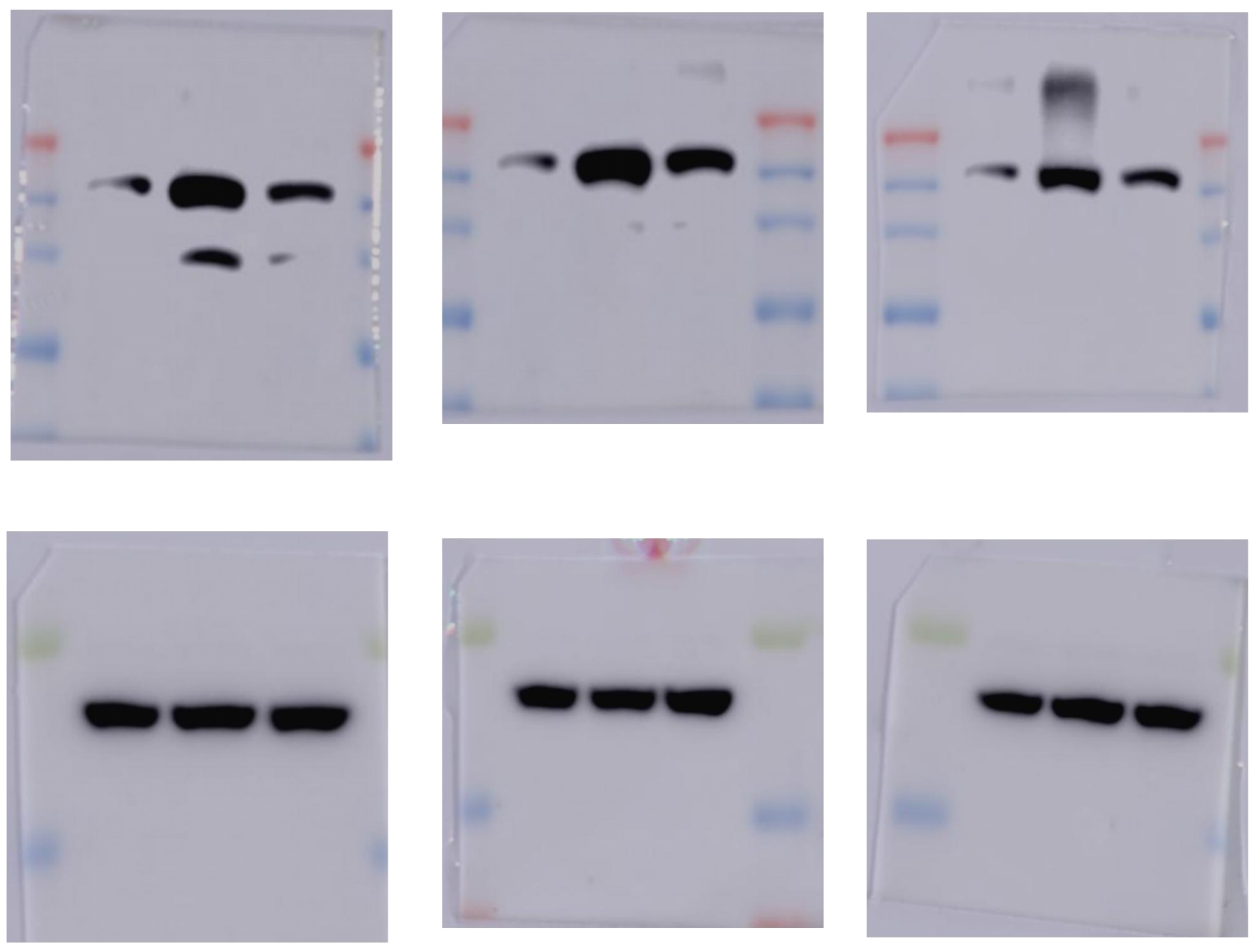

Figure 6SA

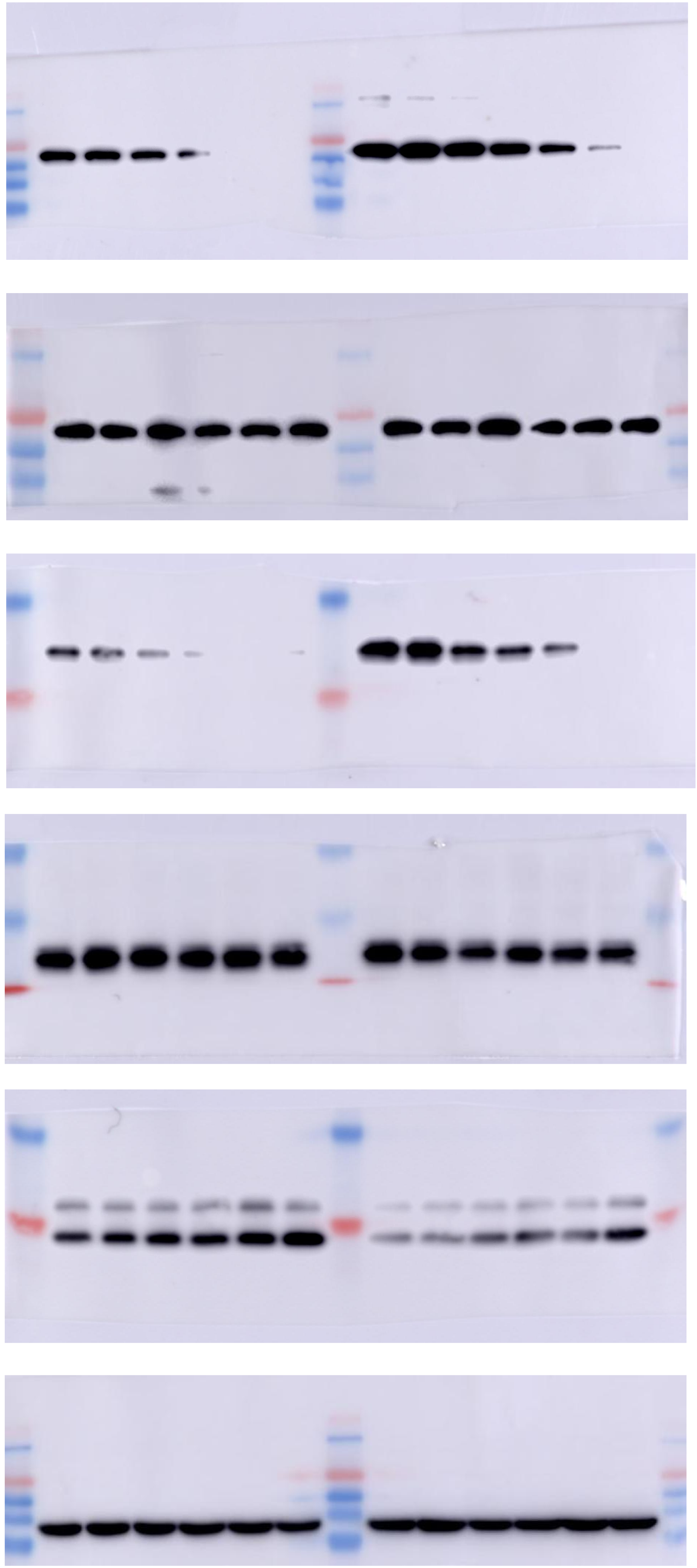

Figure 7SE

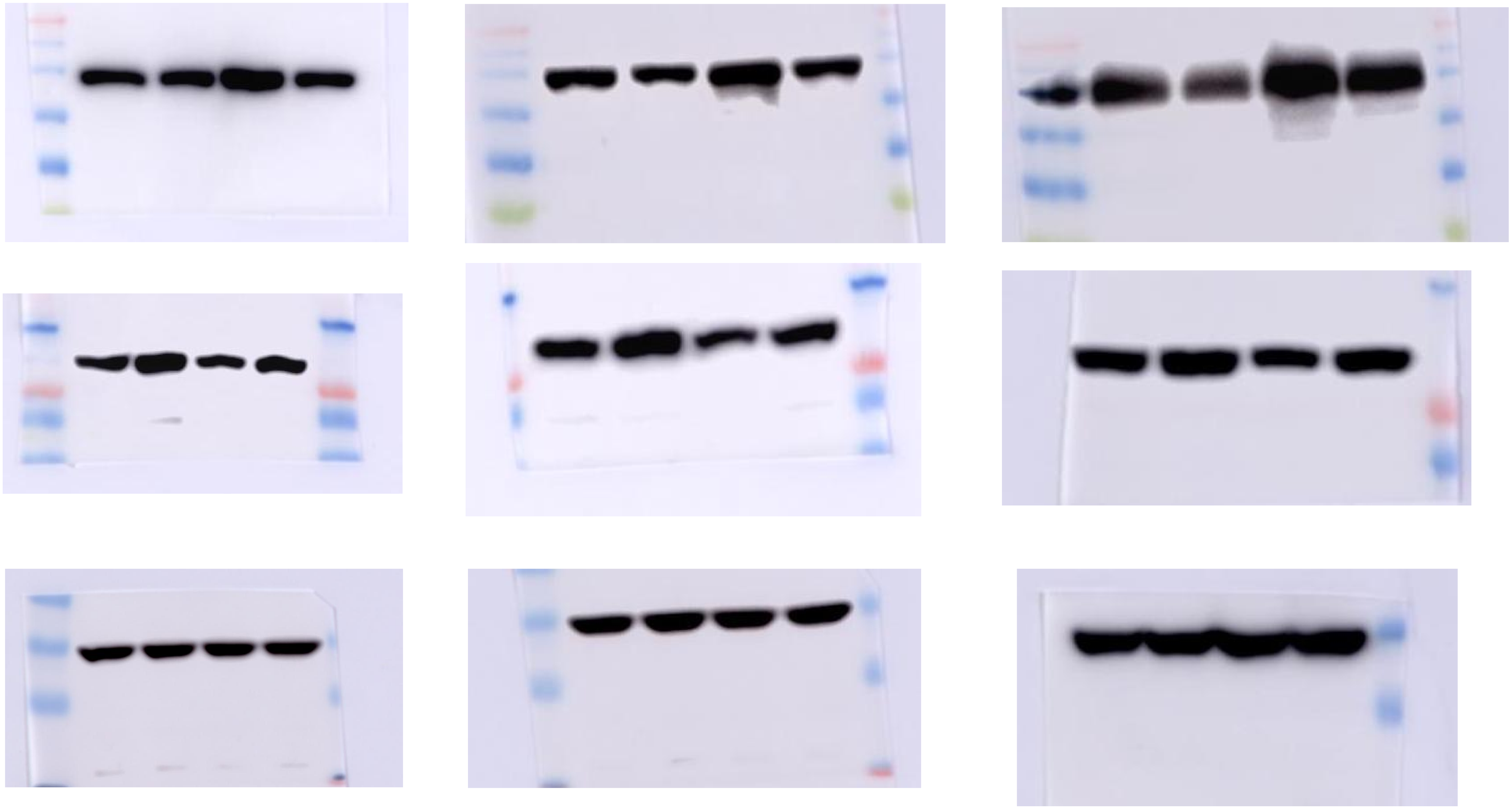

Supplement: Supplementary file 2 — Original Data [file 41419_2024_6808_MOESM2_ESM.pdf]
